# Human Amniotic Epithelial Stem Cell-Derived Retinal Pigment Epithelium Cells Repair Retinal Degeneration

**DOI:** 10.3389/fcell.2021.737242

**Published:** 2021-09-28

**Authors:** Jinying Li, Chen Qiu, Yang Wei, Weixin Yuan, Jia Liu, Wenyu Cui, Jiayi Zhou, Cong Qiu, Lihe Guo, Liquan Huang, Zhen Ge, Luyang Yu

**Affiliations:** ^1^Key Laboratory of Cardiovascular Intervention and Regenerative Medicine of Zhejiang Province of Sir Run Run Shaw Hospital, MOE Laboratory of Biosystems Homeostasis & Protection of College of Life Sciences, Zhejiang University, Hangzhou, China; ^2^College of Life Sciences-iCell Biotechnology Regenerative Biomedicine Laboratory, Joint Research Centre for Engineering Biology, Zhejiang University-University of Edinburgh Institute, Zhejiang University, Haining, China; ^3^School of Pharmaceutical Sciences, Hangzhou Medical College, Hangzhou, China; ^4^Institute of Biochemistry and Cell Biology, Shanghai Institutes for Biological Sciences, Chinese Academy of Sciences (CAS), Shanghai, China

**Keywords:** immune privilege, retinal pigment epithelium, retinal degeneration, human amniotic epithelial stem cells, cell therapy

## Abstract

Age-related macular degeneration (AMD), featured with dysfunction and loss of retinal pigment epithelium (RPE), is lacking efficient therapeutic approaches. According to our previous studies, human amniotic epithelial stem cells (hAESCs) may serve as a potential seed cell source of RPE cells for therapy because they have no ethical concerns, no tumorigenicity, and little immunogenicity. Herein, trichostatin A and nicotinamide can direct hAESCs differentiation into RPE like cells. The differentiated cells display the morphology, marker expression and cellular function of the native RPE cells, and noticeably express little MHC class II antigens and high level of HLA-G. Moreover, visual function and retinal structure of Royal College of Surgeon (RCS) rats, a classical animal model of retinal degeneration, were rescued after subretinal transplantation with the hAESCs-derived RPE like cells. Our study possibly makes some contribution to the resource of functional RPE cells for cell therapy. Subretinal transplantation of hAESCs-RPE could be an optional therapeutic strategy for retinal degeneration diseases.

## Introduction

Age-related macular degeneration (AMD), the typical retinal degeneration disease, is the major cause of irreversible vision loss among senior citizens. The projected number of people with AMD globally is approximately 200 million in 2020, and it is expected to increase to nearly 300 million in 2040 (Wong et al., 2014). Especially, it has been estimated that by 2050, 55.19 million people will be affected by AMD in China (Song et al., 2017). In AMD, the early events of retinal pigment epithelium (RPE) dysfunction usually lead to photoreceptor degeneration, resulting in progressive visual loss and blindness (Luthert, 2011). Patients with wet AMD lose vision because of the growth of abnormal blood vessels (choroidal neovascularization, CNV), while patients with dry AMD, which represents approximately 90% of AMD cases, suffer vision loss as a result of geographic atrophy (GA) of the PRs, RPE, and choriocapillaris in the macular area (Ambati and Fowler, 2012). Currently, antiangiogenic medicine has been well established for CNV. However, there are no effective treatments available for GA (Mitchell et al., 2018). With the development of stem cell technology, cell therapy is a promising therapeutic strategy for retinal degenerative diseases (Ramsden et al., 2013; Trounson and McDonald, 2015; Zarbin, 2016).

Recently, several sources of human pluripotent stem cells (hPSCs) have been tested for their ability to replace damaged or lost RPE cells with seed cells. Human embryonic stem cells (hESCs)- and human induced pluripotent stem cells (hiPSCs)-derived RPE cells have been reported to rescue visual function after subretinal transplantation into animal models, such as Royal College of Surgeon (RCS) rats (Idelson et al., 2009; Hazim et al., 2017; Sharma et al., 2019). Clinical trials that used hESCs- and hiPSCs-derived RPE demonstrated some preliminary, encouraging results (Schwartz et al., 2012, 2015). Both hESCs and hiPSCs possess considerable potential for clinical trials in the treatment of retinal degeneration diseases. However, they are currently limited by their immunogenicity and long-term safety, with chronic immune reaction, macular edema and even DNA aberrations (Mandai et al., 2017; da Cruz et al., 2018; Mehat et al., 2018).

Human amniotic epithelial stem cells (hAESCs), sharing the developmental origin of the pluripotent inner cell mass of blastocysts with hESCs, might be a more useful seed cell source for the replacement of RPE, according to our and other’s previous studies (Li et al., 2018; Miki, 2018; Tan et al., 2018; Yang et al., 2018). First, hAESCs can be isolated from discarded placental tissue without the ethical concerns normally associated with hESCs (Parolini et al., 2008). Second, hAESCs express stem cell surface markers, such as embryonic antigen-3 and -4 (SSEA-3, SSEA-4), tumor rejection antigen 1-60 (TRA1-60), TRA1-81 and molecular markers of pluripotent stem cells, including octamer-binding protein 4 (OCT-4), SRY-related HMG-box gene 2 (SOX-2) and NANOG. Therefore, hAESCs possess the ability to differentiate into all three germ layers, including neurons (ectoderm), cardiomyocytes (mesoderm), and hepatocytes (endoderm), as previously reported (Miki et al., 2005; Miki and Strom, 2006). Additionally, the low immunogenicity of hAESCs is enabled by their high expression of human leukocyte antigen G (HLA-G), a non-classic major histocompatibility complex (MHC) class I molecule, and low expression of MHC class II molecules HLA-DR and HLA-DQ (Strom and Gramignoli, 2016). Finally, hAESCs show no tumorigenicity upon transplantation into both volunteers and patients, which is the key obstacle for the safe clinical use of cell-based regenerative therapies (Akle et al., 1981). This unique property may be due to the lack of telomerase; and the global DNA methylation status of hAESCs is intermediated between hESCs and somatic cells, suggesting that they are genetically stable, in contrast to hESCs (Miki, 2018). These unique characteristics of hAESCs, including pluripotency, low immunogenicity and non-tumorigenicity, make them attractive for the clinical application of retinal diseases.

Here, we report for the first time the direct differentiation of hAESCs into functional RPE like cells, which could rescue retinal function after subretinal transplantation into an animal model of retinal degeneration. The current study might provide a novel cost-efficient and safe therapeutic strategy for the treatment of retinal degeneration diseases.

## Materials and Methods

### Separation of Human Amniotic Epithelial Stem Cells

Human placentas were obtained from healthy mothers who provided written informed consent after undergoing an uncomplicated elective cesarean section as described in our previous methods (Yang et al., 2018). The procedure was approved by the Institutional Patients and Ethics Committee of the International Peace Maternity and Child Health Hospital, Shanghai Jiao Tong University School of Medicine. All donors were negative for hepatitis A, B, C, and D as well as HIV-I and Treponema pallidum (TPAB) antibodies. Briefly, amniotic membranes were isolated and washed with fresh PBS, which was followed by incubation with 0.25% trypsin for 20 min at 37°C in a water bath. Then, hAESCs were centrifuged for 10 min at 300 × *g* and counted. hAESCs were cultured in complete culture medium F12/DMEM containing 10% KnockOut Serum Replacement (KSR), 2 mM L-glutamine, 1% non-essential amino acid, 55 μM 2-mercaptoethanol, 1 mM sodium pyruvate, 1% antibiotic-antimycotic (all from Thermo Scientific, Waltham, MA, United States) and 10 ng mL^–1^ human EGF (Peprotech, Rocky Hill, NJ, United States, Cat# AF-100-15) in a humidified atmosphere of 5.5% CO_2_ at 37°C for three to five days. When cells reached 80% – 90% confluence, they were harvested by incubation with 0.25% trypsin at 37°C for approximately 5 min.

### Differentiation of Retinal Pigment Epithelium Like Cells From Human Amniotic Epithelial Stem Cells

In our study, P0-P1 hAESCs were chosen for investigation. For identification RPE inducer, hAESCs were seeded in 24-well plates and cultured for 7 days with four different concentrations (0.5 μM, 1 μM, 2 μM, and 4 μM) of trichostatin A (TSA). Then, the expression levels of three RPE markers, microphthalmia-associated transcription factor (MITF), orthodenticle homeobox 2 (OTX2), and premelanosome protein (PMEL17), were measured by q-PCR. The transcription factors MITF and OTX2 are early RPE markers, while PMEL17 is a matrix protein present in the melanosome precursors of pigmenting cells. For RPE cell differentiation, hAESCs were seeded in 6-well plates and cultured in DMEM/F12, 15% KnockOut serum, 2 mM glutamine, 1 × non-essential amino acids, and 1 × antibiotic-antimycotic. And When cells reached 80% – 90% confluence, 10 mM NIC (Sigma-Aldrich, Cat# N3376) and 1 μM TSA (APExBIO, Cat# A8183) were added for approximately two weeks, while the medium was changed every day.

### Quantitative Real-Time PCR

Total RNA was extracted from undifferentiated, differentiating hAESCs with an E.N.Z.A. total RNA kit (Omega) according to the manufacturer’s instructions. Reverse transcription was performed using a ReverTra Ace qPCR RT kit (Toyobo). Quantitative real-time PCR was performed with the BioRad iCycler real-time PCR detection system (Bio-Rad) with the primers listed in Supplementary Table 1. PCRs were performed under the following conditions: 95°C for 10 min followed by 40 cycles at 95°C for 10 s, 60°C for 20 s, and 72°C for 15 s. To normalize expression levels, glyceraldehyde 3-phosphate dehydrogenase (GAPDH) was used as an internal control. Quantitative PCR analysis was performed on three biological replicates.

### Immunostaining

After fixation with 4% paraformaldehyde in PBS for 15 min, cells were permeabilized using 0.25% Triton X-100 in PBS for 5–10 min and were blocked for 60 min in 5% goat serum. Then, the cells were incubated for 60 min at room temperature with the following primary antibodies: anti-MITF antibody (Sangon, Cat# D120988, 1:100), anti-ZO-1 antibody (Thermo Fisher Scientific Cat# 40-2200, RRID:AB_2533456, 1:200), anti-Bestrophin antibody (Novus Cat# NB300-164, RRID:AB_10003019, 1:100), anti-RPE65 antibody (Abcam, Cat# ab78036, RRID:AB_1566691, 1:100), anti-PMEL-17 antibody (Abcam, Cat# ab137078, RRID:AB_2732921, 1:100), Cells were then incubated for 120 min at room temperature with the corresponding secondary antibodies: Alexa Fluor 594-conjugated donkey anti-rabbit IgG (Jackson ImmunoResearch, Cat# 711-586-152, RRID:AB_2340622), Alexa Fluor 594-conjugated donkey anti-mouse IgG (Jackson ImmunoResearch, Cat# 715-586-150, RRID:AB_2340857), Alexa Fluor 488-conjugated donkey anti-rabbit IgG (Jackson ImmunoResearch, Cat# 711-546-152, RRID:AB_2340619) and Alexa Fluor 488-conjugated donkey anti-mouse IgG (Jackson ImmunoResearch, Cat# 715-545-150, RRID:AB_2340846). Fluorescence images were acquired with a confocal microscope (Zeiss LSM 800, Carl Zeiss).

### Transmission Electron Microscopy

Cells were fixed with 2.5% glutaraldehyde in 0.1 M cacodylate buffer (pH 7.4) overnight. After washed twice with 0.1 M PBS, cells were fixed with 1% osmium tetroxide for 1 h and then were washed twice with ddH_2_O. Samples were stained with 2% uranyl acetate for 30 min, dehydrated with increasing concentrations of ethanol, and embedded in Agar 100 resin. Ultrathin sections were cut using a diamond knife (Diatome, United States), and sections were stained with 2% uranyl acetate and 2% lead citrate. Micrographs were taken with a JEM-1400 Plus (JEOL) electron microscope.

### Digital Gene Expression Profile

Total RNA was extracted from hfRPE-1, hfRPE-2, and hfRPE-3 cells using TRIzol (Invitrogen) and was provided as a kind gift from Army Medical University (Chongqing, China); and RNA was similarly extracted from hAESCs-RPE-1, hAESCs-RPE-2, and hAESCs-RPE-3 cells. Sequencing libraries were generated using a NEBNext^®^ Ultra^TM^ RNA Library Prep kit for Illumina^®^ (NEB) following the manufacturer’s recommendations, and the quality of libraries was assessed on an Agilent 2100 Bioanalyzer system and ABI StepOnePlus Real-Time PCR System. After cluster generation, the library preparations were sequenced on an Illumina HiSeqTM 4000 platform, and 150 bp single-end reads were generated. Reads per kb per million reads (RPKM) were used to estimate gene expression levels.

### Flow Cytometry

To analyze the expression levels of MHC class II antigens (HLA-DR, BioLegend, Cat# 307603, RRID:AB_314681 and HLA-DQ, BioLegend, Cat#318104, RRID:AB_604128) and HLA-G (BioLegend, Cat# 335909, RRID:AB_10900805), ARPE19 cells (ATCC, RRID:CVCL_0145), human umbilical cord mesenchymal stem cells (hUMSCs, Cat# SHTBA0009C1BAC23 obtained from iCell Biological Technology Co., Ltd), hAESCs, hAESCs-RPE like cells were collected after incubation with 10 ng mL^–1^ IFN-γ (Peprotech, Cat# 300-02-100) for 72 h. Cells were stained with FITC-anti-HLA-DR (isotype control was IgG2a, BioLegend, Cat# 400207, RRID:AB_2884007), FITC-anti-HLA-DQ (isotype control was IgG1, BioLegend, Cat# 400109, RRID:AB_2861401) and APC-anti-HLA-G (isotype control was IgG2a, BioLegend, Cat# 400220, RRID:AB_326468) according to the manufacturer’s instructions, and then they were analyzed by flow cytometry (FACSCalibur; BD Biosciences, Franklin Lakes, NJ). Analyses were performed on five biological replicates.

### Phagocytosis of Photoreceptor Outer Segment

Photoreceptor outer segments (POSs) phagocytosis were performed as described in a previous study (Subrizi et al., 2012). Briefly, the POSs of non-dystrophic RCS rats at 8 weeks of age were detached under dim red light. To assess the specificity of POS phagocytosis, hAESCs-RPE like cells were incubated with POS explants for 6–8 h at 37°C and 5% CO_2_. The internalization of rat POSs by RPE cells was immunostained using an anti-Rhodopsin antibody (Abcam, Cat# ab5417, RRID:AB_304874, 1:200), while ZO-1 (Thermo Fisher Scientific Cat# 40-2200, RRID:AB_2533456, 1:200) was detected to demonstrate the cell morphology. In the Z-stack model, we used a Zeiss LSM 800 confocal microscope to show the location of internalized POS. Five independent experiments were conducted.

### Enzyme-Linked Immunosorbent Assay

hAESCs and hUMSCs (2 × 10^6^) were seeded onto 10 cm dish and were cultured for 72 h. Then the culture medium were collected and telomerase concentration was detected by enzyme-linked immunosorbent assay (ELISA) kit (R&D Systems). hAESCs-RPE like cells were plated at 250,000 cells per cm^2^ and were grown on Transwell membranes (Corning). Cell culture media from the apical and basal sides were collected after 48 h of cell culture. Measurements were performed using five biological replicates. A standard ELISA protocol was followed using vascular endothelial growth factor (VEGF) and pigment epithelium-derived factor (PEDF) ELISA kits (R&D Systems).

### Animals

Royal College of Surgeon (RCS) rats (RRID:RGD_1358258, 3 week old at the time of testing, regardless of sex),a known model of retinal disease, were obtained from the Experimental Animal Center of Army Medical University (Chongqing, China), and they were housed under pathogen-free conditions with a 12 h day-night cycle (lights on at 08:00AM). The animals had access to food and water *ad libitum*, except during test phases. This animal model was chosen because the primary dysfunction of the RPE, a result of a mutation in the receptor tyrosine kinase gene Mertk, leads to secondary degeneration and loss of photoreceptors, which is similar to the progression of human retinal degeneration diseases (D’Cruz et al., 2000). Animals were randomly assigned to the experimental groups. Data collection and evaluation of all experiments were performed blindly. All procedures involving rats were approved by the Laboratory Animal Care and the Use Committee of Zhejiang University (approval number, ZJU20190038). All efforts were made to minimize animal suffering and to reduce the number of animals.

### Transplantation of Human Amniotic Epithelial Stem Cells-Retinal Pigment Epithelium Like Cells in Royal College of Surgeon Rats

After 14 days differentiation, hAESCs-RPE like cells were transplanted into 3-week-old RCS rats. In some experiments, hAESCs-RPE like cells were infected with GFP adenovirus for the convenience of detection. Rats were anesthetized by intraperitoneal injection with a mixture of ketamine (Sigma-Aldrich, 70 mg kg^–1^) and xylazine (Sigma-Aldrich, 6 mg kg^–1^). Local anesthetic drops (benoxinate HCl 0.4%; Fischer Pharmaceuticals, Israel) were administered. To reduce the efflux of cells, the cornea was punctured with a 30-gauge sterile needle (BD). Cell suspensions (1.5 × 10^5^ cells in 2 μL of PBS) were injected into the subretinal space through a small scleral incision with a glass pipette (34-gauge, Hamilton). A sham group was injected with medium alone.

### Electroretinograms

Full-field ERGs were recorded after overnight (> 12 h) dark adaptation as in previously reported (Huo et al., 2012). In brief, rats were anesthetized in dim red light, and the pupils were dilated with compound tropicamide eye drops. The corneal electrodes were placed on each eye after ophthalmic topical anesthesia, with a subdermal reference electrode and a ground electrode placed in the cheek and tail, respectively. A computerized ERG system (Q450, ROLAND CONSMLT, Germany) was used to record retinal responses to full-field stimuli. Dim white flashes (−40, −25, −10, 0 and + 5 db) under scotopic conditions were used to elicit mixed cone-rod responses (a largely rod-driven response), and signal averaging was used.

### Histological and Immunohistochemical Evaluation of Transplanted Eyes

The eyeballs were fixed in 4% formaldehyde for 24 h, dehydrated with 70% alcohol, embedded in paraffin and serially cut to produce 3 μm-thick sections. Slides were stained with hematoxylin and eosin (H&E) according to a standard protocol. For immunostaining, eyecups were directly frozen in OCT (Tissue-Tek) and were cut to generate 3 μm-thick sections. Sections were fixed in acetone for 10 min at −20°C and then were washed with PBS, which was followed by incubation with blocking buffer (1% BSA and 5% HBS in PBS) for one h at room temperature. After blocking, sections were incubated for 1 h in a humidified chamber with the following primary antibodies: anti-CRALBP (Abcam, Cat# ab15051, RRID:AB_2269474, 1:100), anti-RPE65 (Abcam, Cat# ab78036, RRID:AB_1566691, 1:100), Then, the sections were incubated for 14 h with secondary antibodies. Nuclei were counterstained with DAPI (DAKO). Fluorescence images were acquired with a confocal microscope (Zeiss LSM 800, Carl Zeiss).

### Quantification of Retinal Thickness

The full length of the retina was scanned from high-resolution microscopic images of H&E-stained sections with NDP.view2 software. Total retinal and ONL thicknesses were measured in proximity to subretinal injection site and corresponding opposite side of the retina via the ImageJ (NIH). In each area, 3 equally spaced measurements were taken.

### Statistical Analysis

Studies were designed to generate groups of almost equal size by using randomization and blinded analysis. The statistical analysis was undertaken only for studies where each group size was at least *n* = 3. All experimental animals were treated with independent values without technical replicates. Statistical analysis was performed using GraphPad Prism 6 (GraphPad, RRID:SCR_002798). Data are presented as the mean ± SEM. Comparisons were performed using unpaired *t* tests, one-way ANOVA or two-way ANOVA followed by Tukey’s multiple comparisons test. *Post hoc* tests were conducted only if *F* in ANOVA achieved *p* < 0.05. The significance level for all tests was set at ^∗^*p* < 0.05.

## Results

### The Characterization of Human Amniotic Epithelial Stem Cells

**FIGURE 1:**
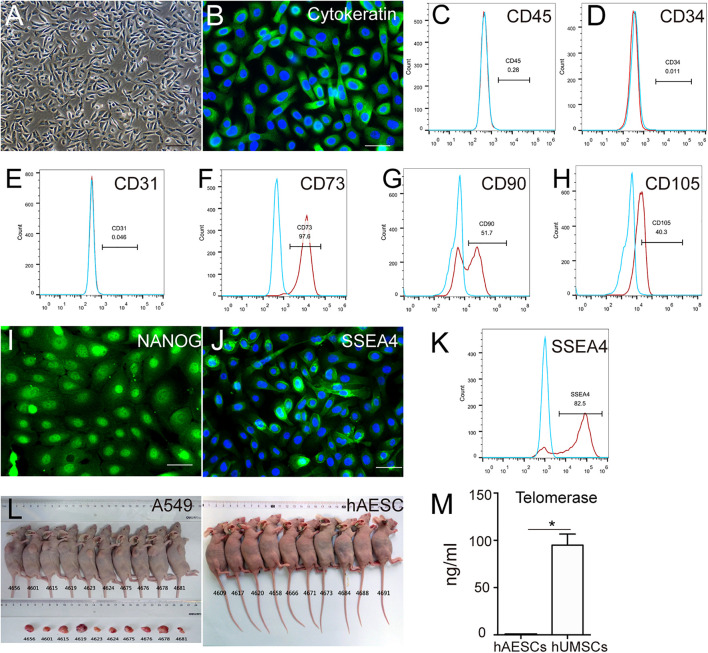
Characteristics of hAESCs. **(A)** Phase-contrast microscope image showed the isolated hAESCs as a homogeneous population with cobblestone appearance. **(B–E)** Nearly all cells demonstrated persistent expression of representative epithelial marker cytokeratin as determined by immunofluorescence microscopy and negative for hematopoietic lineage marker CD45 and CD34, endothelial cells markers CD31 as determined by flow cytometry. **(F–H)** MSC markers CD73, CD90, CD105 were detected by flow cytometry. **(I–K)** Strong expression of pluripotency markers NANOG and SSEA4 were determined by immunofluorescence microscopy and flow cytometry. **(L)** No tumor formation was observed in Balb/c nude mice with subcutaneous injection of hAESCs for 55 days, compared with obvious tumor formation in positive control mice receiving non-small lung cancer A549 cells. **(M)** ELISA analysis showed the low expression of telomerase compared with mesenchymal stem cells (MSCs). Score bars, 100 μm in **(A)**, 50 μm in **(B)** and **(I–J)**, error bars represent mean ± SEM of three biological replicates (*n* = 3), *p < 0.05, unpaired *t*-test.

According to our previous studies (Li et al., 2018; Tan et al., 2018), we first confirmed the purity, pluripotency and non-tumorigenicity of isolated hAESCs, and we cultured them in a serum-free system for further study. The cultured hAESCs showed the typical appearance of epithelial cells and high expression of representative epithelial marker pan-cytokeratin (Figures 1A,B). Meanwhile, negative expression of the hematopoietic lineage markers CD45, CD34 and endothelial marker CD31 were detected by flow cytometry, indicating the purity of hAESCs without contamination (Figures 1C–E). As reported, hAESCs were partly positive for the mesenchymal stem cells (MSCs) markers CD73, CD90 and CD105 (Figures 1F–H). The expression of pluripotent markers NANOG and SSEA4 proved the plasticity of hAESCs as seed cells (Figures 1I–K). Moreover, no tumor formation was observed in Balb/c nude mice with subcutaneous injection of hAESCs for 55 days, compared with obvious tumor formation in positive control mice receiving non-small lung cancer A549 cells (Figure 1L). The low expression of telomerase compared with MSCs may explain the non-tumorigenicity (Figure 1M). In summary, these results indicate that hAESCs are a novel type of epithelial stem cells without tumorigenicity.

### Trichostatin A Plus Nicotinamide Were Determined as Appropriate Chemical Cocktail for Human Amniotic Epithelial Stem Cells Differentiation Toward Retinal Pigment Epithelium Fate

**FIGURE 2:**
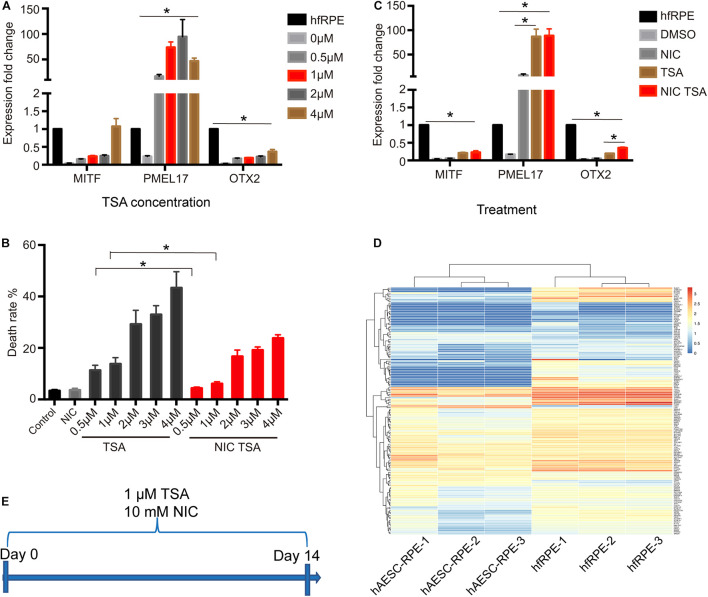
Identification of TSA as a potent promoter of RPE differentiation. **(A)** RPE markers expression in hAESCs at day 7 following TSA treatment, as measured by quantitative PCR. Expression levels of TSA treated hAESCs were normalized by expression levels of hfRPE. **(B)** The cell death rates of hAESCs in the presence of NIC with different concentrations of TSA at day 7 following treatment. **(C)** RPE markers expression in hAESCs at day 7 following 10 mM NIC and 1 μM TSA treatment alone or combined, as measured by quantitative PCR. Expression levels of treated hAESCs were normalized by expression levels of hfRPE. **(D)** Heat map of 149 RPE signature genes in hAESCs-RPE and native human fetal RPE (hfRPE). Normalized expression levels are shown in a blue-white-red gradient. **(E)** Schematic of hAESCs differentiation into hAESCs-RPE using a chemical cocktail of NIC and TSA. Error bars represent mean ± SEM of three biological replicates (*n* = 3). MITF, microphthalmia-associated transcription factor; PMEL17, premelanosome protein; OTX2, orthodenticle homeobox 2; *p < 0.05, compared with control group; unpaired *t*-test, one-way ANOVA followed by Tukey’s multiple comparisons test.

To determine the scheme for RPE differentiation, hAESCs were cultured for 7 days with four different concentrations of compounds. One compound, trichostatin A (TSA), was identified as an inducer of RPE differentiation from hAESCs, according to the expression levels of three RPE markers, microphthalmia-associated transcription factor (MITF), orthodenticle homeobox 2 (OTX2), and premelanosome protein (PMEL17) as described in the Methods and Materials. Upon the administration of TSA, all three RPE markers in treated hAESCs were upregulated consistently in a dose-dependent pattern. The expression levels of MITF and OTX2 were upregulated in the presence of TSA alone, while the expression of PMEL17 was even strikingly upregulated (Figure 2A). Meanwhile, high concentrations of TSA caused obvious cell death. Considering the induction efficiency and cell death rates, 1 μM was determined as the optimum concentration of TSA for RPE differentiation. Given the role of nicotinamide (NIC) in protecting hPSCs from cell death during neuroectoderm differentiation and the positive effect on RPE differentiation (Idelson et al., 2009; Maruotti et al., 2015), we tested the effect of NIC on the differentiation of hAESCs. As expected, NIC could prevent excessive cell death and augment the expression of OTX2 during TSA-directed RPE differentiation (Figures 2B,C). Altogether, these data suggest that during NIC and TSA differentiation, RPE markers are strongly up-regulated without excessive cell death.

To determine the optimal time period of the combined treatment, the expression of key markers during RPE differentiation was further assessed in the presence of NIC and TSA at sequential time points. Most RPE markers were upregulated, while dopachrome tautomerase (DCT) was downregulated after 2 weeks of differentiation (Supplementary Figures 2A–E). To comprehensively investigate hAESCs differentiation, their transcriptomes were examined after 2 weeks of induction. According to previous reports (Strunnikova et al., 2010), the levels of 149 representative genes during RPE differentiation were analyzed in differentiated hAESCs, and they were compared with native human fetal RPE (hfRPE) cells. A heat map of normalized expression levels of each group showed a similar expression pattern between hAESCs-RPE cells and hfRPE cells (Figure 2D). To investigate the efficiency of the differentiation of hAESCs into RPE, RPE related markers were detected by flow cytometry. Results showed that more than 90% differentiated cells were positive for MITF, PMEL17, Bestrophin and RPE65 (Supplementary Figures 3A–D). In summary, the optimal scheme for inducing hAESCs differentiation into RPE cells was determined and is shown in schematic diagram (Figure 2E).

### Human Amniotic Epithelial Stem Cells-Derived Retinal Pigment Epithelium Like Cells Demonstrated Typical Retinal Pigment Epithelium Cellular Characteristics and Functions *in vitro*

**FIGURE 3:**
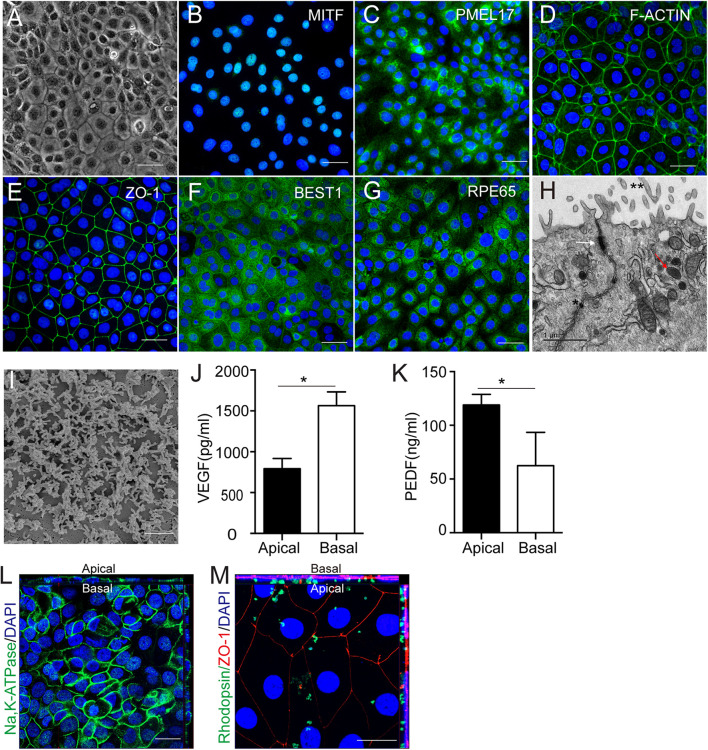
Cellular characteristics and functions of hAESCs-RPE like cells obtained by combined induction of NIC and TSA. **(A)** Morphology of hAESCs-RPE cells after 2 weeks induction as shown in phase-contrast microscope image, with pigmented and polygonal pattern. **(B–C)** The expression of signature early RPE markers MITF, PMEL17 in hAESCs-RPE after 1 week induction were demonstrated by immunofluorescence microscopy. **(D–E)** Phalloidine staining indicated distribution of F-actin adjacent to the cell membrane and the expression of mature RPE marker ZO-1 indicated the tight junction formation in hAESCs-RPE after 10 days induction. **(F–G)** The expression of signature mature RPE markers BEST1, RPE65 in hAESCs-RPE like cells after 2 weeks induction were demonstrated by immunofluorescence microscopy. **(H–I)** Detailed feature characteristic of RPE in hAESCs-RPE after 2 weeks induction were shown by electron microscopy: apical microvilli (double asterisk), melanin granules (red arrowhead) and tight junctions (asterisk and white arrowhead) by transmission electron microscopy, and the apical microvilli by scanning electron microscopy. **(J–K)** Polarized secretion of VEGF-A and PEDF from the apical and basal sides of hAESCs-RPE after 2 weeks induction were shown by ELISA analysis. **(L)** Confocal Z-stack fluorescent image with cross-section side views showing the typical apical localization of Na-K ATPase in hAESCs-RPE after 2 weeks induction. **(M)** Confocal Z-stack fluorescent image with cross-section side views showing phagocytosis of photoreceptor outer segment purified from non-dystrophic RCS rats (green-rhodopsin) by hAESCs-RPE after 2 weeks induction. Nuclei were counterstained with DAPI (blue) in panel **(C,D,E,G,L,M)**. Score bars, 50 μm in **(A–G,L–M)**,1 μm in **(H)**, 2 μm in **(I)**, error bars represent mean ± SEM of three biological replicates (*n* = 3), *p < 0.05, unpaired *t*-test.

After 2 weeks of culture with NIC and TSA, the induced hAESCs developed an RPE like morphology that was pigmented and polygonal (Figure 3A). Immunostaining indicated the strong expression of signature early RPE markers such as MITF and PMEL17 in differentiated cells on day 7 after the combined treatment (Figures 3B,C and Supplementary Figure 2F). Staining also demonstrated abundant F-actin distribution adjacent to the cell membrane and high expression of the tight junction protein ZO-1 among differentiated cells on day 10 after combined treatment, which are data that are similar to those of naturally developing RPE cells (Figures 3D,E). At the late stage of differentiation (after 2 weeks of combined treatment), strong expression of the mature RPE markers of chloride channel-related protein Bestrophin 1 (BEST1) and retinal pigment epithelium-specific protein of 65 kids (RPE65) was detected in the differentiated cells (Figures 3F–G and Supplementary Figure 2F). Additionally, RPE ultrastructure’s, including the presence of apical microvilli, pigment granules and tight junctions and well-developed apical microvilli, were revealed by transmission electron microscopys (TEM) and scanning electron microscopy (SEM), respectively (Figures 3H,I).

The RPE functions of the differentiated cells were further examined *in vitro*. As a polar monolayer between the neural retina and the choriocapillaris, RPE cells play crucial roles by secreting nutrition factors, phagocytosing the photoreceptor outer segment (POS) and forming the blood-retinal barrier. According to a previous report, vascular endothelial growth factor (VEGF) and pigment epithelium-derived factor (PEDF) were secreted preferentially to the basal and apical sides, respectively, by native RPE cells (Maruotti et al., 2015). Consistently, ELISA results verified similar VEGF and PEDF expression patterns in differentiated cells (Figures 3J,K). Z-stack images of confocal microscopy showed that Na/K ATPase is largely apical *in situ* in differentiated cells, indicating polarity that is similar to that of RPE cells (Lehmann et al., 2014) (Figure 3L). To determine the phagocytosis function, the differentiated cells were incubated with rat POSs and were then examined by confocal microscopy. The Z-stack images showed that ZO-1-labeled differentiated cells could phagocytose rhodopsin-labeled POSs (Figure 3M). Herein, these results demonstrate that hAESCs progress into a polarized and functional monolayer of RPE like cells following small molecule induction for about14 days.

### The Immune Privilege of Human Amniotic Epithelial Stem Cells-Derived Retinal Pigment Epithelium Like Cells

**FIGURE 4:**
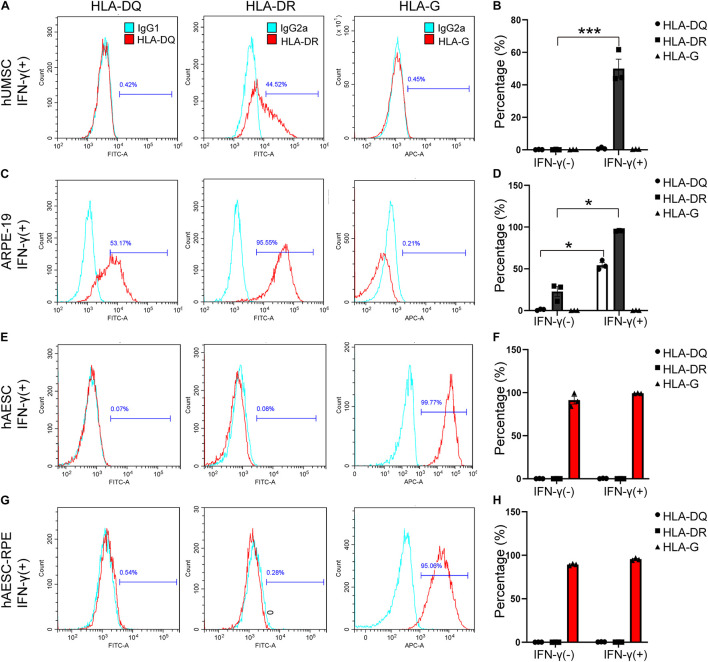
Consistent low immunogenicity in hAESCs and their derived cells under proinflammatory condition. Flow cytometry analysis of HLA-DQ, HLA-DR, and HLA-G in human umbilical cord mesenchymal stem cells (hUMSC), human retinal epithelial cell line ARPE-19, hAESCs, hAESCs-RPE like cells with 10 ng/mL IFN-γ treatment for 72 h **(A,C,E,G)**, indicating consistent low immunogenicity in hAESCs and their derived cells under proinflammatory condition. Representative histograms of HLA-DQ, HLA-DR, and HLA-G are shown in red and the isotype controls are shown in blue. Under normal and proinflammatory conditions, the quantitative results were shown in **(B,D,F,H)**. Error bars represent mean ± SEM of three biological replicates (*n* = 3). *p < 0.05, ***p < 0.001, unpaired *t*-test.

Additionally, the immunogenicity of the differentiated cells was examined before subretinal transplantation. The flow cytometry results showed very low levels of HLA-DR and HLA-DQ and high expression of HLA-G (that counteracts NK cell function) in hAESCs-RPE like cells, similar to what were observed in hAESCs with or without IFN-γ treatment (Figures 4E–H and Supplementary Figures 4C,D). However, significant expression of HLA-DR and HLA-DQ was detected in the human retinal epithelial cell line ARPE-19, and significant expression of HLA-DR was detected in IFN-γ-treated human umbilical cord mesenchymal stem cells (hUMSCs), while very low level of HLA-G were detected in both ARPE-19 and hUMSCs (Figures 4A–D and Supplementary Figures 4A,B).

### Subretinal Transplantation of Human Amniotic Epithelial Stem Cells-Retinal Pigment Epithelium Rescued Retinal Structure and Visual Function *in vivo*

**FIGURE 5:**
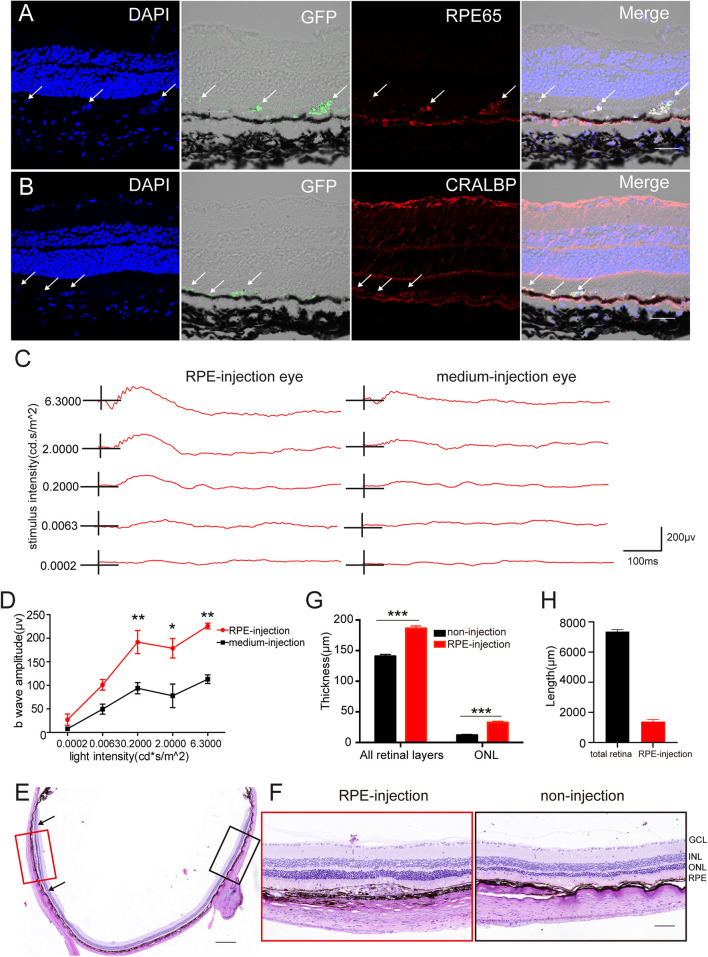
Subretinal transplantation of hAESCs-RPE like cells rescued retinal structure and visual function in RCS rats. Retinal structure and function were examined 4 weeks after subretinal transplantation of hAESCs-RPE. **(A–B)** Immunofluorescence microscopy showed GFP-labeled transplanted hAESCs-RPE coexpressing RPE markers RPE65 and CRALBP. Nuclei were counterstained with DAPI (blue). Note differential interference contrast combined with fluorescent image indicating the integration of GFP + hAESCs-RPE within the host RPE layer; arrows showing transplanted hAESCs-RPE. **(C)** Representative ERG responses to a series of white flashes of increasing intensity in the dark-adapted state in a transplantation eye and its fellow un-treatment eye. **(D)** Mean b-wave amplitudes in response to white flashes of increasing intensity, showing significantly higher read in transplanted eyes (red line) as compared to groups of control eyes. **(E–G)** Representative images of H&E stained retina sections with histological quantifications, showing preservation of ONL and thicker whole retina in subretinal transplantation region (indicated by red frame in E and higher-magnification image in the left panel of **F**, with quantification in **G**) as compared with thinner ONL and whole retina in the region distant from graft in the same eye (indicated by black frame in E with higher-magnification image in the right panel of **F**, with quantification in **G**). **(H)** The length of the retina affected by the cell transplantation was quantificated by ImageJ (located between black arrowheads). Score bars, 50 μm in **(A–B)**, 500 μm in **(E)** and 100 μm in **(F)**, error bars represent mean ± SEM of three biological replicates, *p < 0.05, **p < 0.01, ***p < 0.001; two-way ANOVA followed by Tukey’s multiple comparisons test **(D)**; unpaired *t*-test **(G)**; *n* = 3 rats per group.

To further determine whether hAESCs-RPE like cells had a therapeutic effect on retinal degeneration *in vivo*, hAESCs-RPE like cells, differentiated for 14 days *in vitro*, were transplanted into the subretinal space of RCS rats, which are an animal model of retinal degeneration in which the defect of RPE to phagocytize POSs leads to further degeneration and progressive loss of PRs (D’Cruz et al., 2000). Immunohistochemistry demonstrated the subretinal localization of the GFP-labeled engrafted cells, which expressed RPE-specific markers RPE65 and CRALBP (Figures 5A,B). In addition, color fundus imaging illustrated that the transplantation of hAESCs-RPE cells mitigated the retinal disorder comparing to the pale retina with unnormal pigment in control eyes (Supplementary Figure 5). Of note, the differential interference contrast combined with the fluorescent image indicated the presence of the transplanted hAESCs-RPE within the host RPE layer.

Importantly, an electroretinographic (ERG) response assay at 4 weeks after transplant revealed a significant preservation of visual function in cell-grafted eyes compared with medium-injected eyes (Figure 5C). There were significantly greater b-wave amplitudes detected at a series of luminance levels in the dark-adapted state of cell-grafted eyes than there were in the control groups (Figure 5D).

Furthermore, histological analysis demonstrated that organized retina structure, especially the outer nuclear layer (ONL), was extensively preserved in a region adjacent to the cell injection site compared with obvious ONL loss in opposite non-transplantation regions (Figures 5E,F). This was confirmed by the quantification of the ONL thickness in both groups (Figure 5G) and retinal length affected by the cell transplantation was quantificated by ImageJ (Figure 5H). Around 20% of the retina is affected by the hAESCs-RPE transplantation.

To assess the long-term therapeutic effects of hAESCs-RPE cells and to substantiate the efficacy and safety of the cell transplantation, cell-grafted and matching control groups were followed up to 8 weeks after subretinal transplantation. Immunostaining and histological analysis indicated the survival of transplanted RPE cells and showed their protective effect in retinal degeneration (Supplementary Figures 6A–E).

## Discussion

Cell transplantation of RPE cells is one of the most promising therapeutic strategies for incurable retinal degenerative diseases (Jones et al., 2017; Stern et al., 2018). Our study indicated that hAESCs might serve as an optional source for retinal cells, and they could be induced into RPE like cells under defined culture conditions. Human amniotic epithelial stem cells-derived RPE like cells exhibit the morphology and marker expression of native retinal cells and can partially improve retinal structure and function after subretinal transplantation into a classical animal model of retinal degeneration. But we need to be aware that the induced cells are not comparable to the native RPE cells, especially lower expression of some of the RPE signature markers. Therefore, the induced cells are termed as “RPE like cells.” Longer differentiation time, 3D culture carrier and additional screening of small molecules aiming the low expression markers may improve the quality of the induced cells, which will be conducted in our future studies. According to our previous studies, hAESCs possessed special properties as seed cells for cell therapy, which mainly include no ethical concerns related to their harvest and clinical application, their immunomodulatory properties, and their absence of tumorigenicity because of lack of telomerase (Li et al., 2018; Yang et al., 2018).

One of the major concerns for cell therapy is graft rejection. Employing autologous stem cells seems to be a feasible way to overcome this obstacle, but in some cases, patients with dry AMD were reported to lose light perception caused by retinal detachment after receiving an intravitreal transplantation of autologous human adipose-derived stem cells (hADSCs) (Kuriyan et al., 2017). Moreover, there are currently no reliable endogenous differentiation strategies for retinal cells, and their collection requires invasive extraction (Moviglia et al., 2012; Rezanejad et al., 2014). Additionally, their immunogenicity induction abilities are unpredictable and there is even debate regarding their tumorigenicity in pathological microenvironments in some cases (Barkholt et al., 2013; Casiraghi et al., 2013; Veceric-Haler et al., 2017). Thus, allogeneic stem cells, especially hiPSCs and hESCs, are considered the main choice as seed cells for retinal disease therapy. Along with research and development, retinal cells derived from hiPSCs and hESCs, however, cannot avoid confronting an increased risk of rejection and the challenge of tumorigenicity. Clinical trials have reported that immunosuppression is always required after hES-RPE transplantation (da Cruz et al., 2018; Mehat et al., 2018). Moreover, macular edema and even DNA aberrations occurred in the hiPSCs-RPE recipient (Mandai et al., 2017). In the present study, we also paid attention to the immunogenicity of hAESCs-derived retinal cells. Our work and others’ demonstrated the immune tolerance capacity of hAESCs due to high expression of a non-classic MHC class I molecule HLA-G that impairs NK cell recognition and killing, and the weak expression of MHC class II antigens (Strom and Gramignoli, 2016; Tan et al., 2018; Yang et al., 2018). To our surprise, the differentiated cells still kept low levels of HLA-DR, DQ and high expression of HLA-G that were similar to the levels in undifferentiated hAESCs, in spite of proinflammatory stimulation. However, obvious immunogenicity was observed in the human ARPE19 cell line and IFN-γ treated human MSCs in the present study, suggesting potential graft rejection and correlated immune reactions during cell therapy. These result to not administer immunosuppressive agents to the host rats of cell transplantation. Moderate survival of engrafted cells and their effect on retinal function restoration were observed even 2 months later, while human cell rejection was observed in animal models to some extent. Coupling with the non-tumorigenicity property of hAESCs that were mentioned, our results may indicate the low immunogenicity and potential safety of hAESCs-derived RPE like cells. The success of long-term xenotransplantation may herein lead to the expectation of a better performance of hAESCs-derived RPE in clinical use. Moreover, we infer that the combined transplantation of non-differentiated hAESCs with hAESCs-RPE may achieve a better therapeutic effect, because non-differentiated hAESCs could improve the microenvironment based on their immunomodulatory properties. It is worthy to investigate the co-transplantation strategy in our future studies.

In the present study, we identified TSA, a histone deacetylase inhibitor, as an inducer of RPE differentiation. On the other hand, during hAESCs-RPE like cells differentiation, the key RPE markers were upregulated by TSA in a dose-dependent pattern (0.5 μM to 4 μM), indicating that a high concentration of TSA promoted differentiation efficiency. Nevertheless, a high ratio of cell death was observed in that circumstance. To solve this problem, NIC was introduced in the culture medium, as NIC has been reported to protect hPSCs from cell death during neuroectoderm differentiation through PARP1 inhibition (Idelson et al., 2009). Indeed, the cotreatment of TSA and NIC seem to facilitate both efficiency and viability of hAESCs-RPE like cell differentiation. The attractive findings in the current study are the preservation of vision function and retinal structure by hAESCs-derived RPE like cells. These rescue abilities are closely correlated with the f ate of differentiated cells within the host RPE layer *in vivo*.

In summary, we may contribute to expansion of RPE cell resources for replacement therapy. The hAESCs-RPE differentiation and subretinal transplantation could be a potential therapeutic strategy for treating retinal diseases such as AMD. Further preclinical studies are required to determine the effect of hAESCs-RPE cells on different types of retinal diseases such as AMD, retinitis pigmentosa (RP) and stargardt disease (SD). Although these retinal diseases have different causes and demographics, they share common pathology of RPE degeneration at their end-stage. Thus, it is worthy to investigate the therapeutic effect of the hAESCs-RPE in a wider range of animal models that correspond to retinal degeneration disease in clinic. On the other hand, more details of cell delivery need to be determined to pave the road for the fulfillment of clinical treatment in future. In this category, the comparisons may focus on the cellular product forms as cell suspension, cell preparations on a biocompatible scaffold or cell preparation in a sheet without a subjacent scaffold, the administration approaches by intravitreal injection or subretinal injection, as well as the optimal doses for single or multiple injections.

### Limitation

Several limitations of the present study need to be noticed. First, the detailed working mechanism of TSA need to be dissected, in the direction of epigenetic regulation. Second, a systemic safety evaluation of the hAESCs-RPE is required for the aim of clinical application, although their proper homogeneity and low immunogenecity has been identified. Third, the time points for observation *in vivo* is limited, especially lack of longer time points more than 3 months, which is important for defining the survival duration of the transplanted cells. Fourth, further investigation is required to determine the details of the transplanted cells such as their integration within the host RPE layer and their interaction with host cells.

## Data Availability Statement

The datasets presented in this study can be found in online repositories. The names of the repository/repositories and accession number(s) can be found below: Gene Expression Omnibus, accession number: GSE180616.

## Ethics Statement

The studies involving human participants were reviewed and approved by the Institutional Patients and Ethics Committee of the International Peace Maternity and Child Health Hospital, Shanghai Jiao Tong University School of Medicine. The patients/participants provided their written informed consent to participate in this study. The animal study was reviewed and approved by the Laboratory Animal Care and the Use Committee of Zhejiang University (approval number, ZJU20190038).

## Author Contributions

LY and ZG: study design, review and edited the manuscript. JYL and ChQ: wrote the manuscript, data generation, and analysis. YW, WY, JL, WC, CoQ, LG, and LH: data interpretation and review the manuscript. All authors approved the final version of the manuscript.

## Conflict of Interest

JYL, ChQ, WY, JL, and LY are coinventors on the patent application related to the use of hAESCs for RPE differentiation (China Patent Serial No. ZL201910310260.1). The remaining authors declare that the research was conducted in the absence of any commercial or financial relationships that could be construed as a potential conflict of interest.

## Publisher’s Note

All claims expressed in this article are solely those of the authors and do not necessarily represent those of their affiliated organizations, or those of the publisher, the editors and the reviewers. Any product that may be evaluated in this article, or claim that may be made by its manufacturer, is not guaranteed or endorsed by the publisher.
