# Supplementary material for: Human Amniotic Epithelial Stem Cell-Derived Retinal Pigment Epithelium Cells Repair Retinal Degeneration
Source: Front Cell Dev Biol. 2021 Sep 28;9:737242. doi: 10.3389/fcell.2021.737242 (PMC8505778; doi:10.3389/fcell.2021.737242)
Supplement: Supplementary file 1 [file Table_1.docx]

Supplementary Material

**Supplementary Figures**


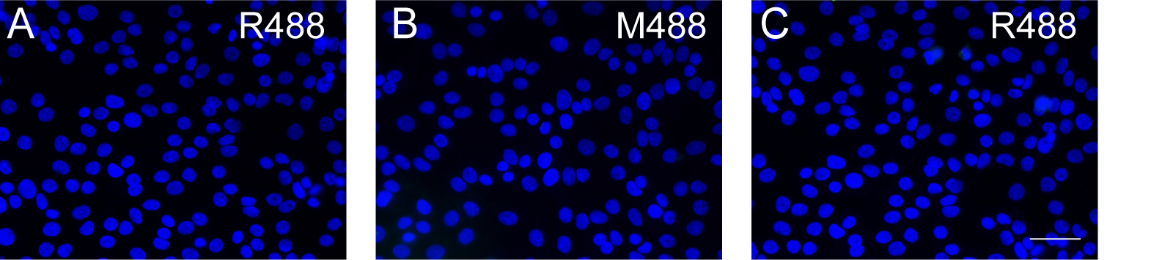


**Supplementary Figure 1.** (A-C) The negtive controls of immunostaining in figure1(B, I, J). R488: rabbit 488, M488: mouse 488. Scale bars: (A-C) 50 μm.


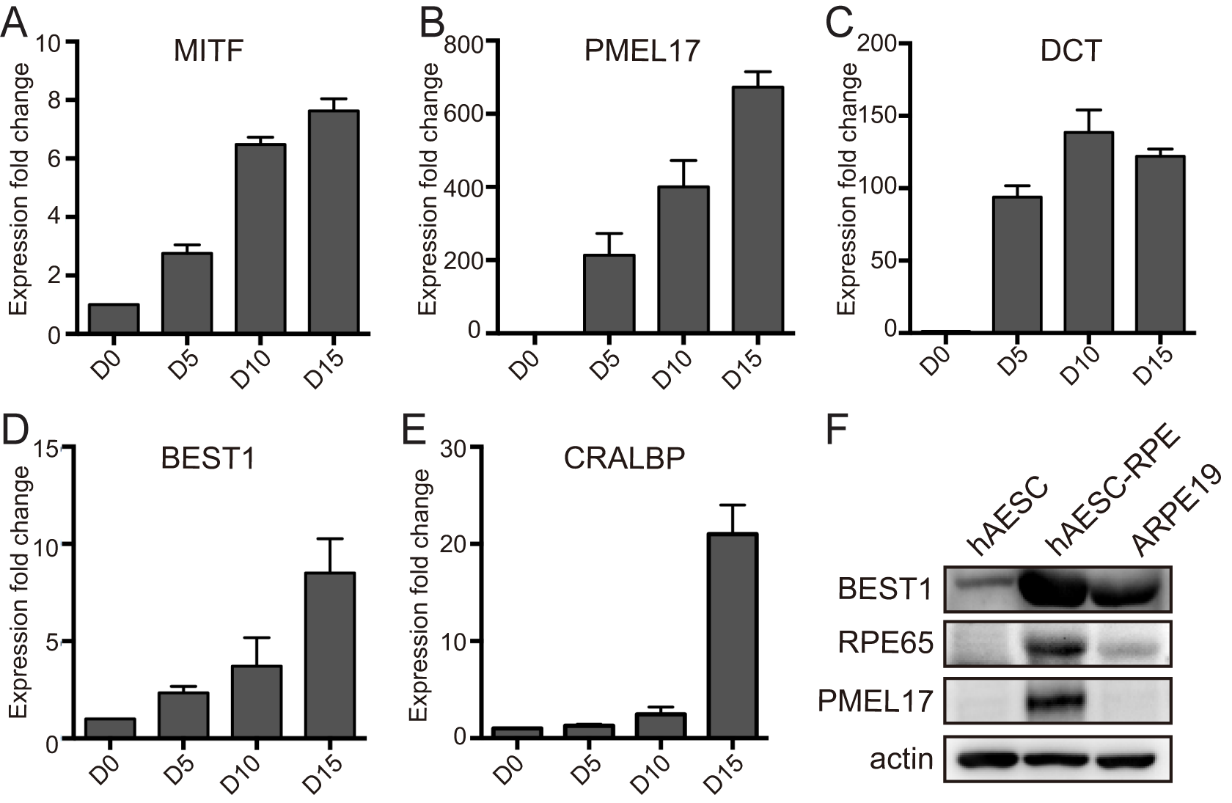


**Supplementary Figure 2. Expression of signature RPE markers during hAESCs-RPE differentiation.** hAESCs were induced for hAESCs-RPE as indicated in Figure1E and the expression of signature RPE markers at sequential time points were determined by quantitative PCR. Expression levels of induced hAESCs were normalized by expression levels of the non-treated hAESCs (D0). (**A**) early RPE marker: MITF. (**B-C**) key RPE markers for melanin synthsis: PMEL17 and DCT. **(D-E**) mature RPE markers: BEST1 and CRALBP. (**F**) Protein levels of key RPE markers (BEST1, RPE65 and PMEL17) were detected by Western blotting at the end of differentiation. Error bars were represented SEM of three biological replicates. DCT, dopachrome tautomerase; BEST1, bestrophin 1; CRALBP, retinaldehyde binding protein 1; ARPE19, Adult Retinal Pigment Epithelial cell line-19.


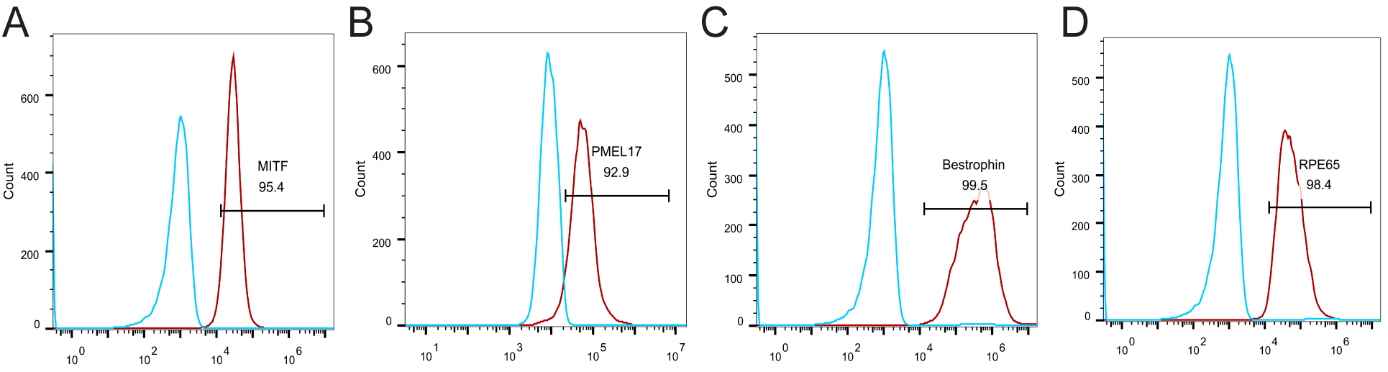


**Supplementary Figure 3. The efficiency of the differentiation of hAESCs into RPE.** RPE related markers were detected by flow cytometry. Results showed that more than 90% differentiated cells were postive for MITF (A), PMEL17 (B), Bestrophin (C) and RPE65 (D). Representative histograms of MITF, PMEL17, Bestrophin and RPE65 are shown in red and the isotype controls are shown in blue.


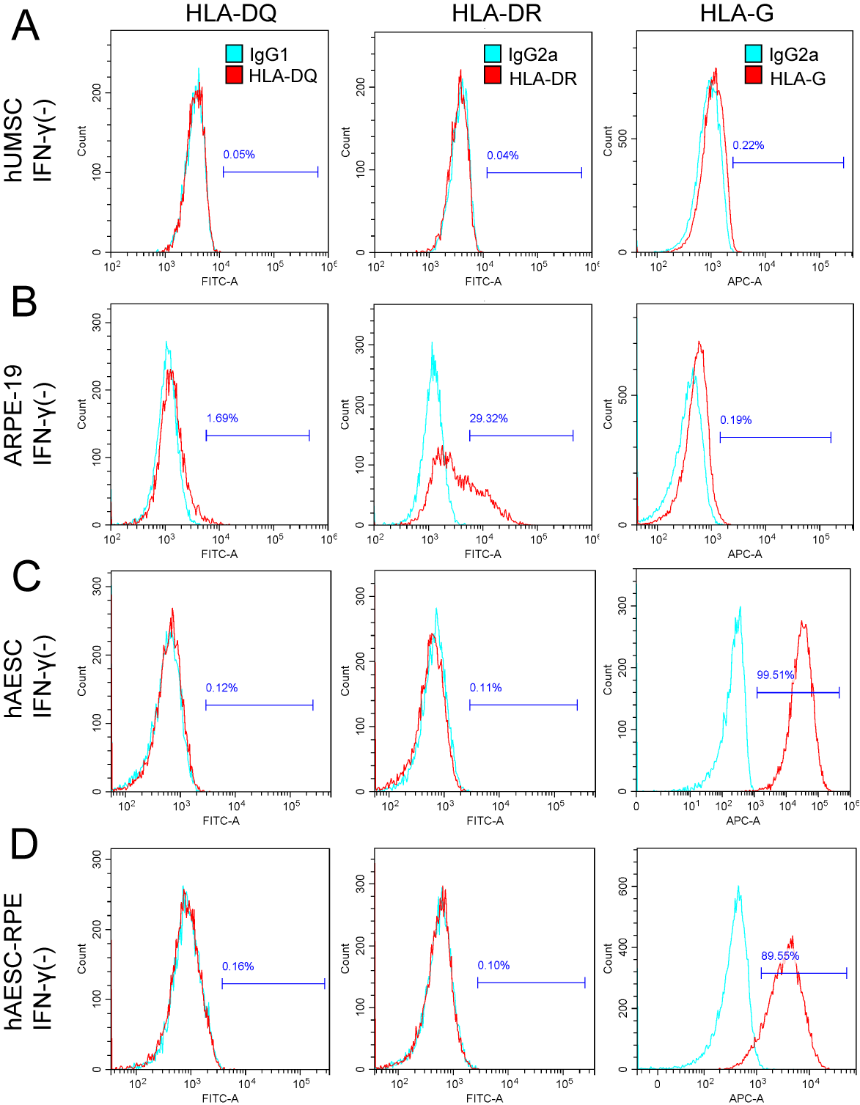


**Supplementary Figure 4. Consistent low immunogenicity in hAESCs and their derived cells under normal condition.** Flow cytometry analysis of HLA-DQ, HLA-DR and HLA-G in human umbilical cord mesenchymal stem cells (hUMSC), human retinal epithelial cell line ARPE-19, hAESCs, hAESCs-RPE like cells in normal culture medium. Representative histograms of HLA-DQ, HLA-DR and HLA-G are shown in red and the isotype controls are shown in blue.


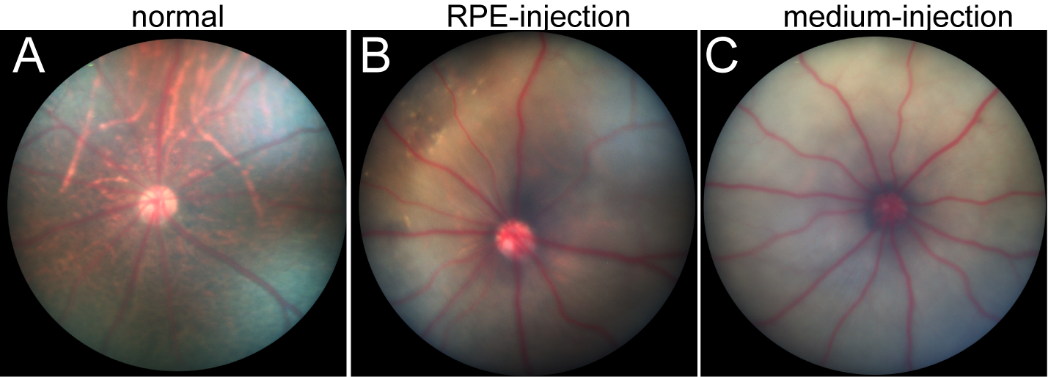


**Supplementary Figure5.** Representative color fundus images of normal rat eye (**A**), hAESCs-RPE injection eye of RCS rat (**B**), and medium-injection eye of RCS rat (**C**). Note the transplantation of hAESCs-RPE cells mitigated the retinal disorder comparing to the pale retina with unnormal pigment in control eyes.


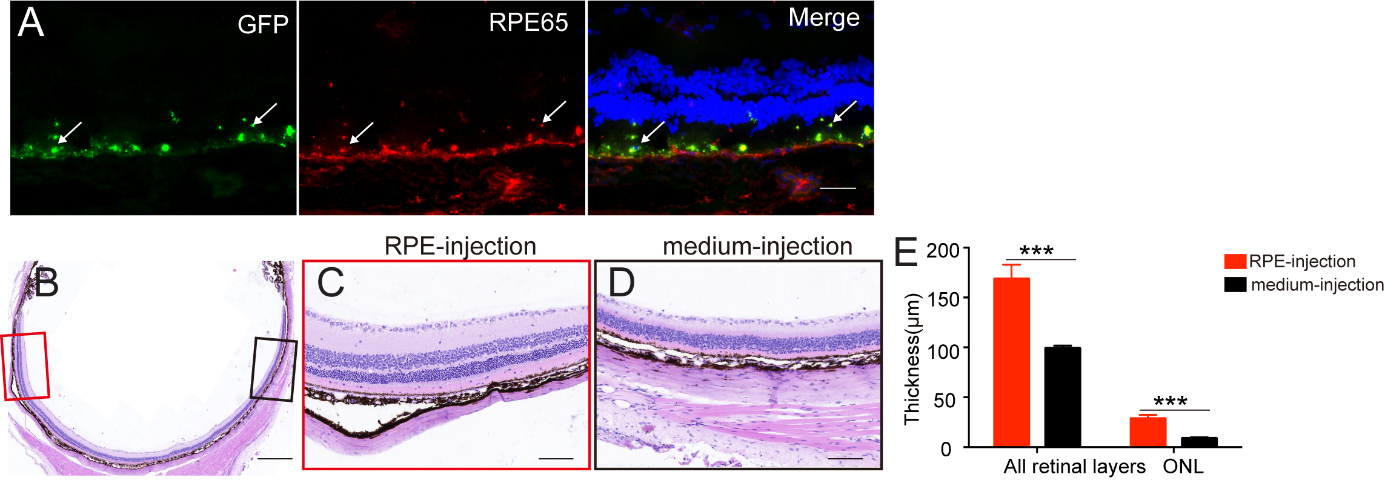


**Supplementary Figure 6. hAESCs-RPE like cells survive and provide long-term retinal preservation in RCS rats after subretinal transplantation.** (**A**) Immunoﬂuorescence microscopy showed GFP-labeled transplanted hAESCs-RPE coexpressing RPE markers RPE65. Nuclei were counterstained with DAPI (blue). Note the integration of some GFP^+^ hAESCs-RPE within the host RPE layer; arrows showing transplanted hAESCs-RPE. (**B-E**) Representative images of H&E stained retina sections with histological quantifications, showing preservation of ONL and thicker whole retina in subretinal transplantation region (indicated by red frame in B and higher-magnification image in C, with quantification in E) as compared with thinner ONL and whole retina in the region distant from graft in the same eye (indicated by black frame in B with higher-magnification image in D, with quantification in E). Scale bars: (A) 50 μm, (B) 500 μm, (C-D) 100 μm. Data are presented as the mean ± SEM of three biological replicates. ****p*<0.001, two-way ANOVA followed by Bonferroni post-test.

**Supplementary Tables**

**Supplementary Table 1. Primers Used in q-PCR.**

| **Primer** | **Sequence** |
| --- | --- |
| PMEL-F | ATAGGTGCTTTGCTGGCTGT |
| PMEL-R | CTTGACCACCTCTCCAGCA |
| MITF-F | TTGCAACGAGAACAGC/lACG |
| MITF-R | GAGGTCTTGGCTGCAGTTCT |
| BEST1-F | TCTGAGCCTACCCTTCCTCC |
| BEST1-R | TCCTTCACCTGGTCCCAAGA |
| CRALBP-F | CCCCGCCACACCTTGCAGAA |
| CRALBP-R | TTCCGTGCGCGGATGAAGCG |
| DCT-F | TGGGAGGAACGAGTGT |
| DCT-R | CCAGGTGGTTGTAGTCA |
| OTX2-F | CAAAGTGAGACCTGCCAAAAAGA |
| OTX2-R | TGGACAAGGGATCTGACAGTG |
