# Supplementary material for: Human Amniotic Epithelial Stem Cell-Derived Retinal Pigment Epithelium Cells Repair Retinal Degeneration
Source: Front Cell Dev Biol. 2021 Sep 28;9:737242. doi: 10.3389/fcell.2021.737242 (PMC8505778; doi:10.3389/fcell.2021.737242)
Supplement: Supplementary file 2 [file Table_2.DOCX]

https://www.jianguoyun.com/p/DX45xfMQi6bjCRiN94kE
