# Supplementary figures and images for: Human Amniotic Epithelial Stem Cell-Derived Retinal Pigment Epithelium Cells Repair Retinal Degeneration
Source: Front Cell Dev Biol. 2021 Sep 28;9:737242. doi: 10.3389/fcell.2021.737242 (PMC8505778; doi:10.3389/fcell.2021.737242)

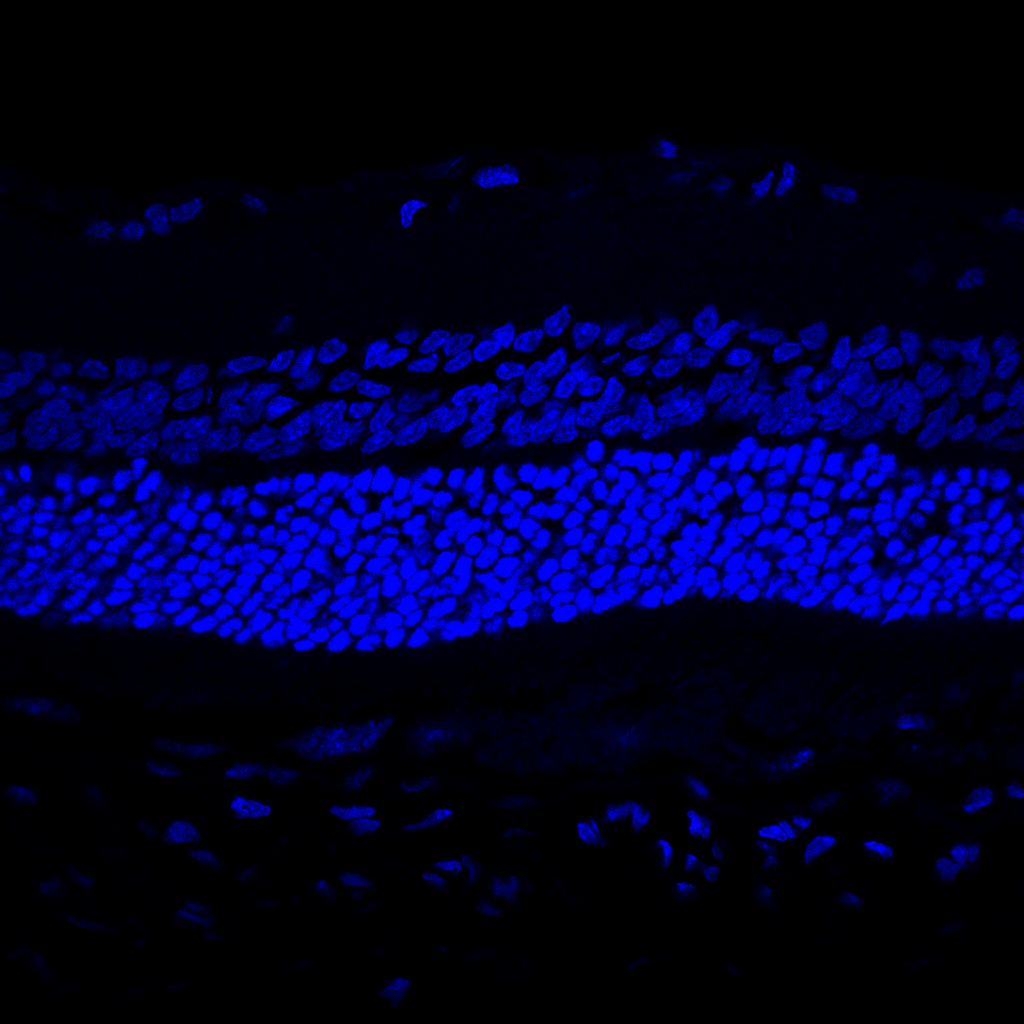

Supplement: Supplementary file 3 [file Data_Sheet_1.ZIP › raw data 2/figure5A B.tif]

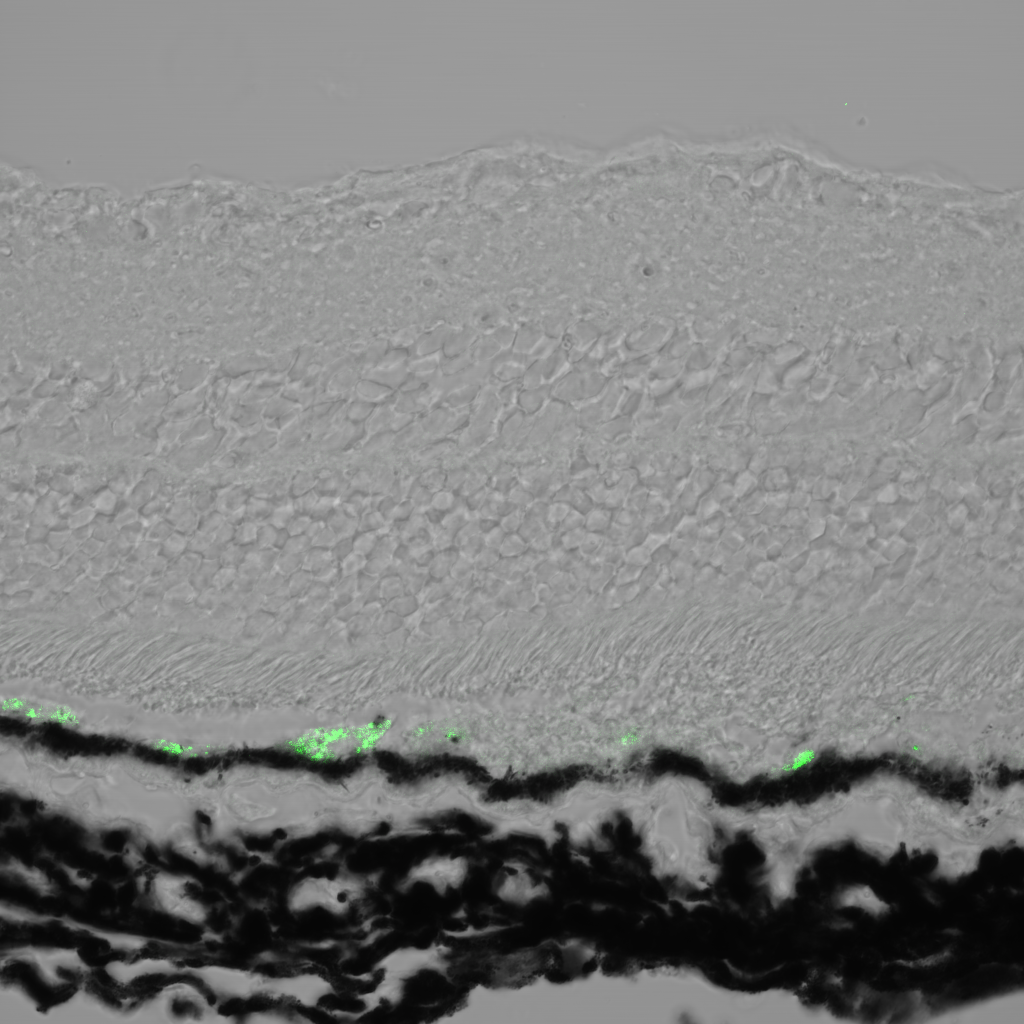

Supplement: Supplementary file 3 [file Data_Sheet_1.ZIP › raw data 2/figure5A G.tif]

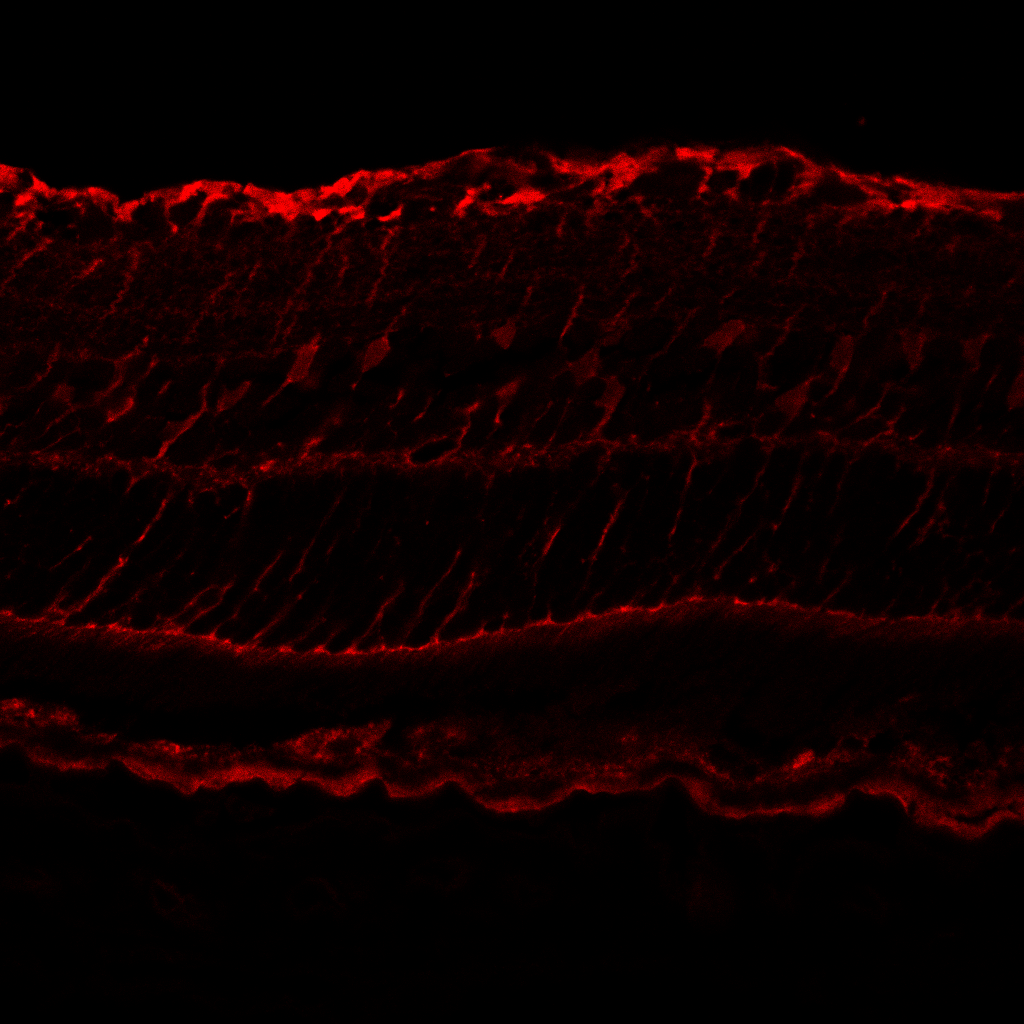

Supplement: Supplementary file 3 [file Data_Sheet_1.ZIP › raw data 2/figure5A R.tif]

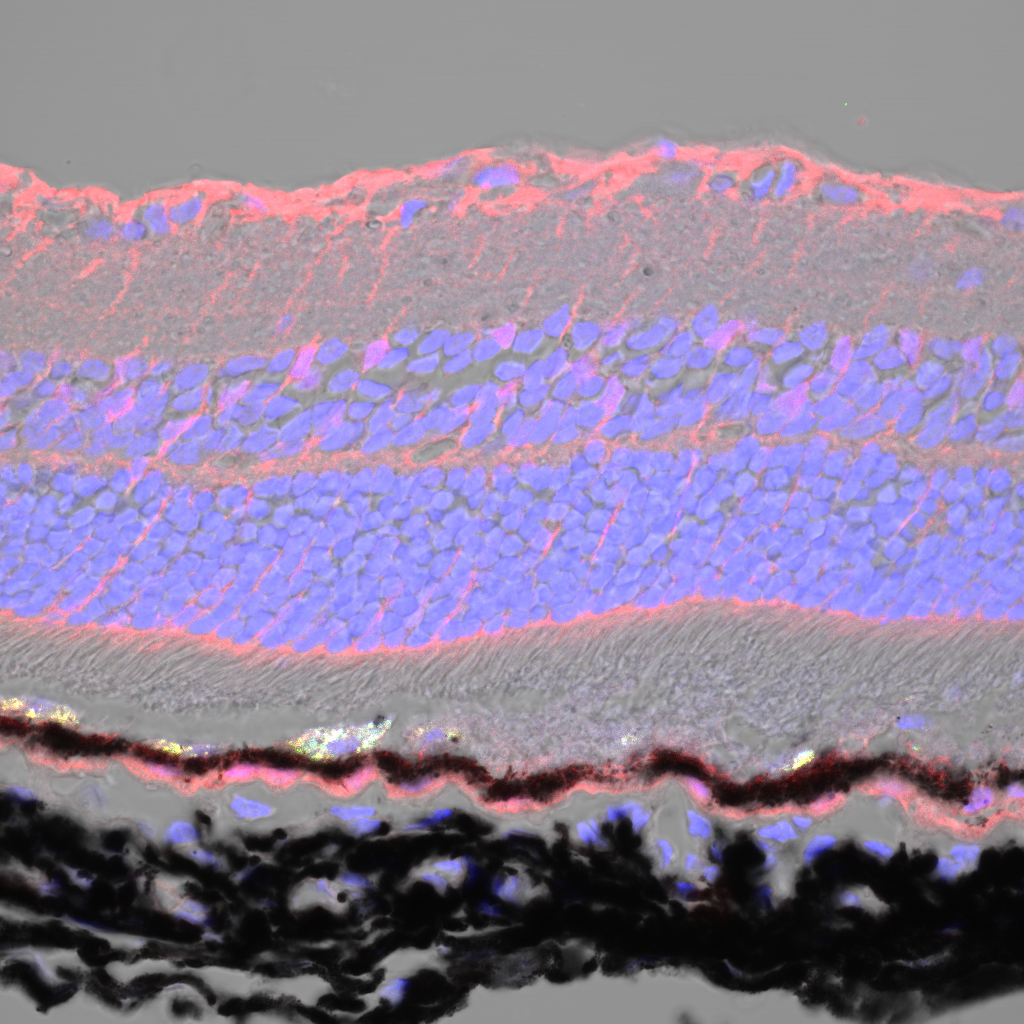

Supplement: Supplementary file 3 [file Data_Sheet_1.ZIP › raw data 2/figure5A.tif]

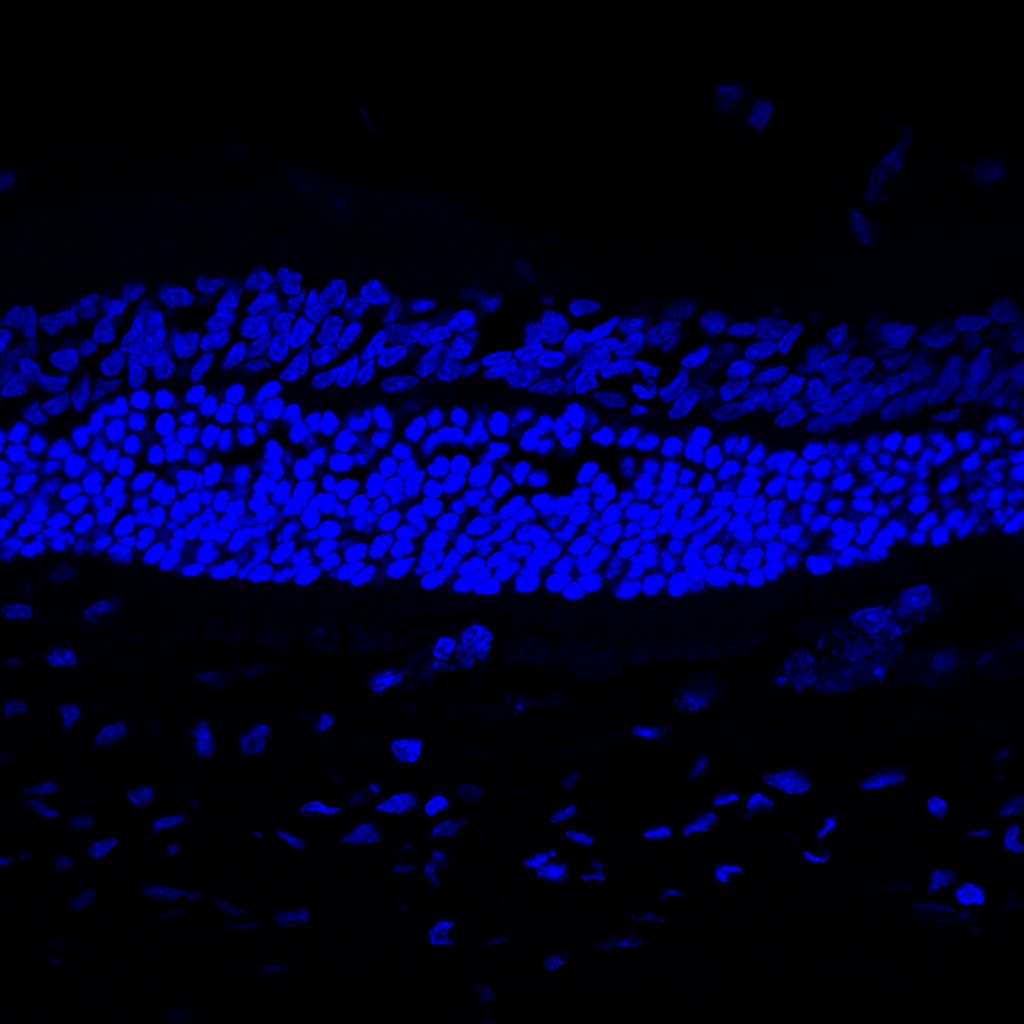

Supplement: Supplementary file 3 [file Data_Sheet_1.ZIP › raw data 2/figure5B B.tif]

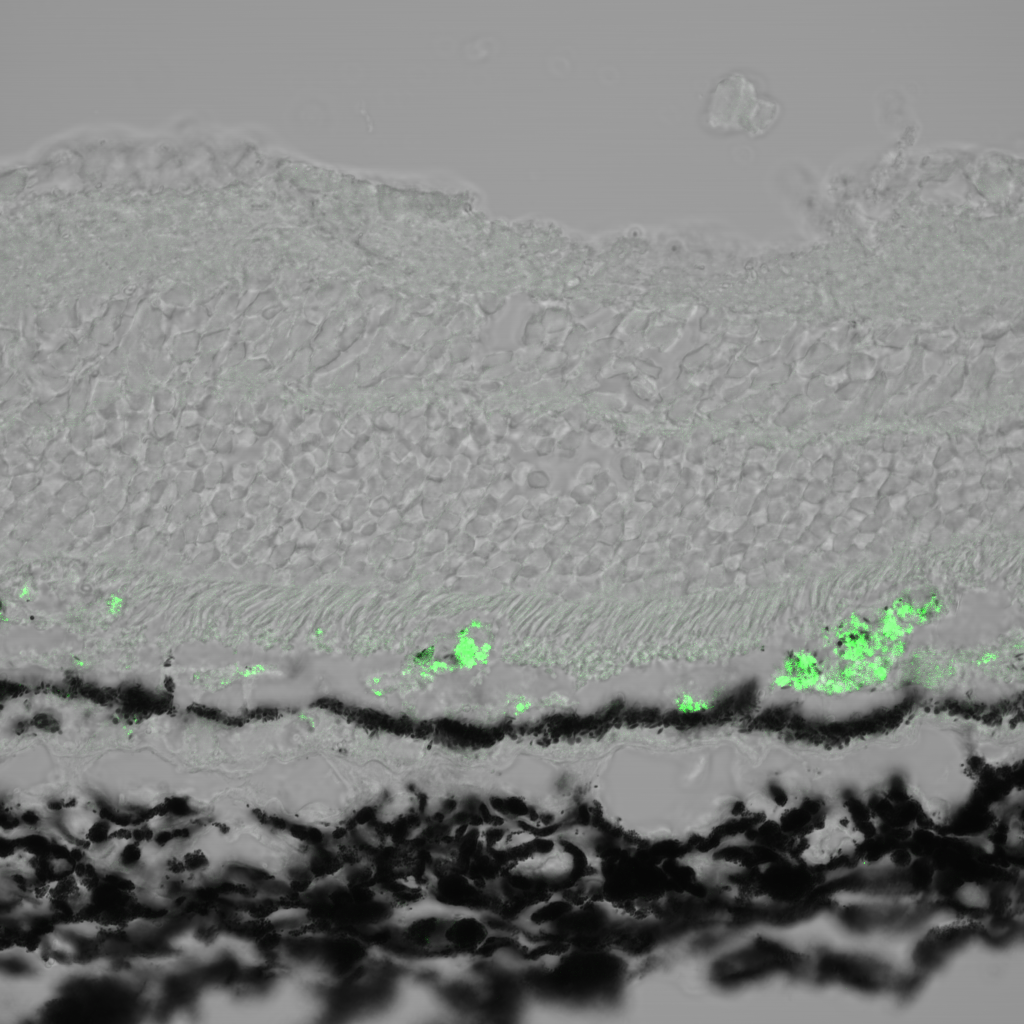

Supplement: Supplementary file 3 [file Data_Sheet_1.ZIP › raw data 2/figure5B G.tif]

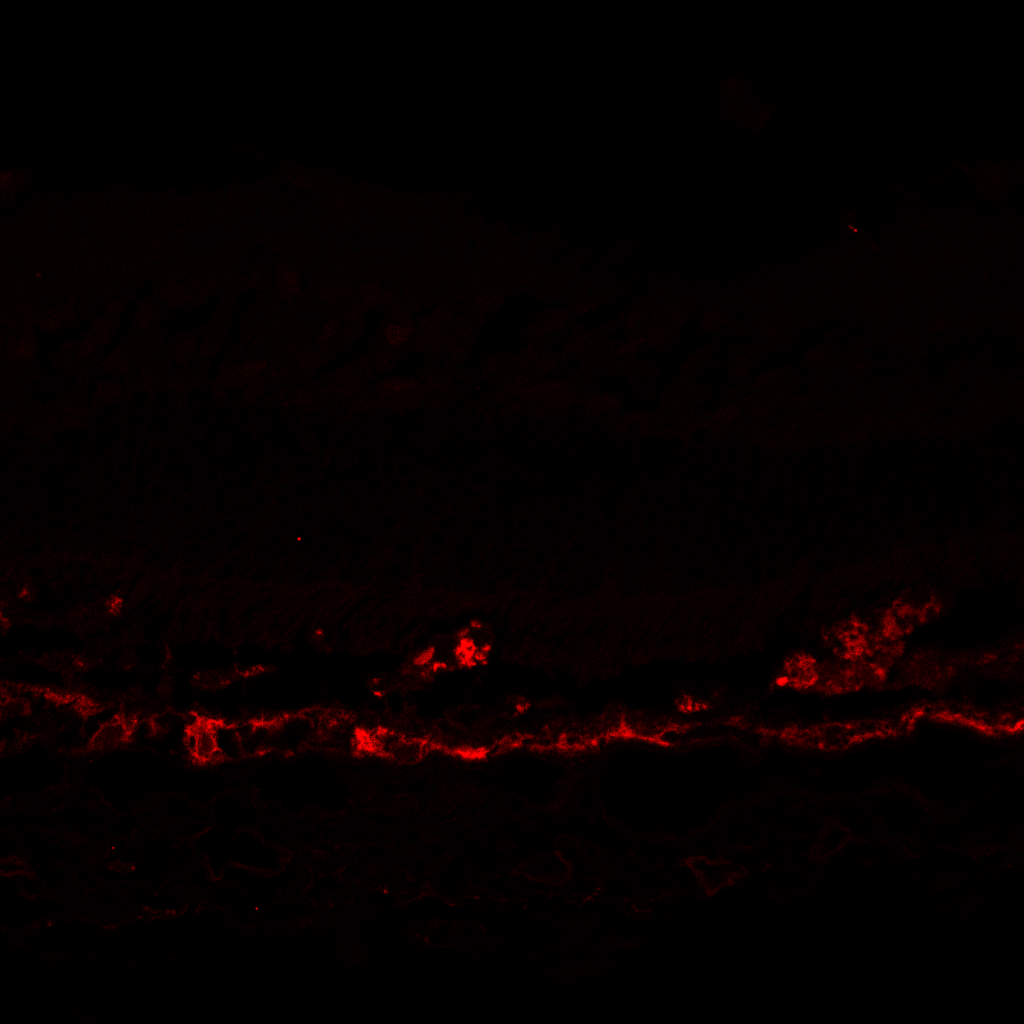

Supplement: Supplementary file 3 [file Data_Sheet_1.ZIP › raw data 2/figure5B R.tif]

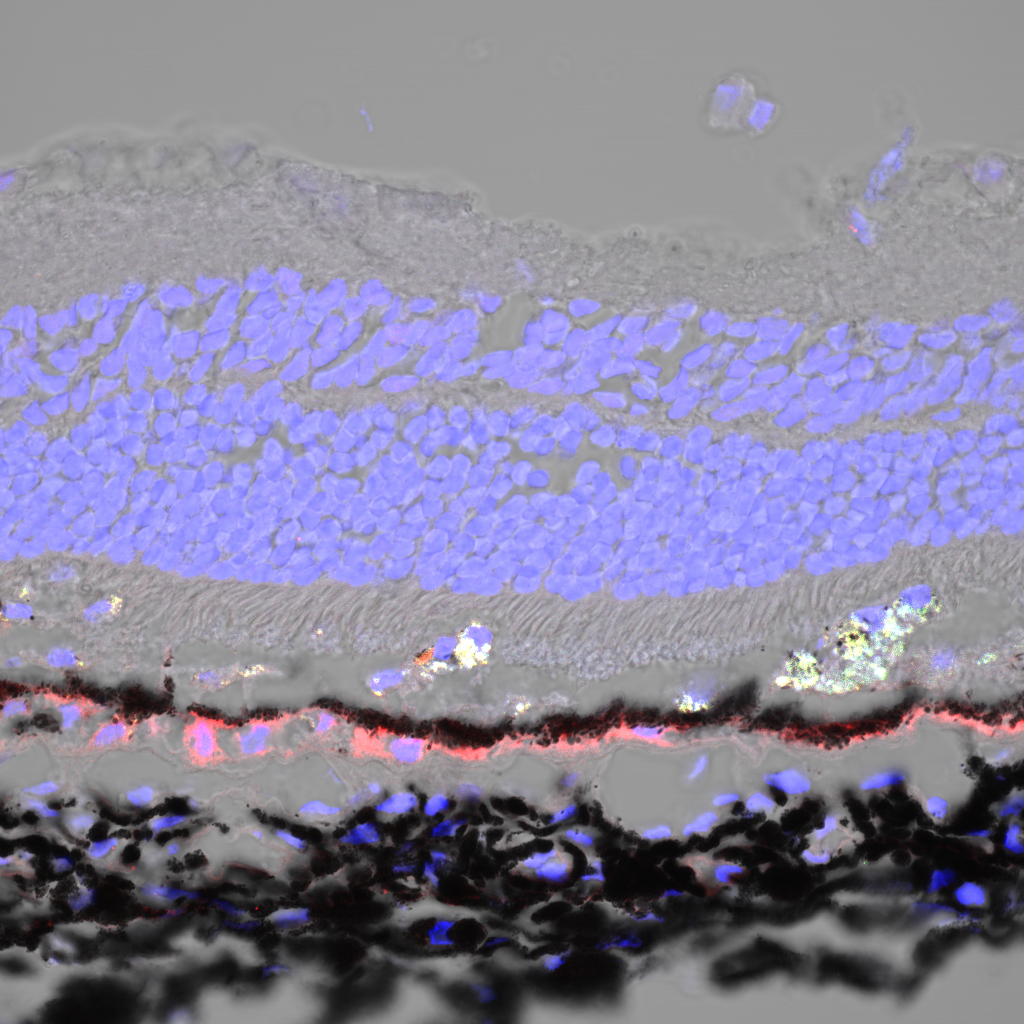

Supplement: Supplementary file 3 [file Data_Sheet_1.ZIP › raw data 2/figure5B.tif]

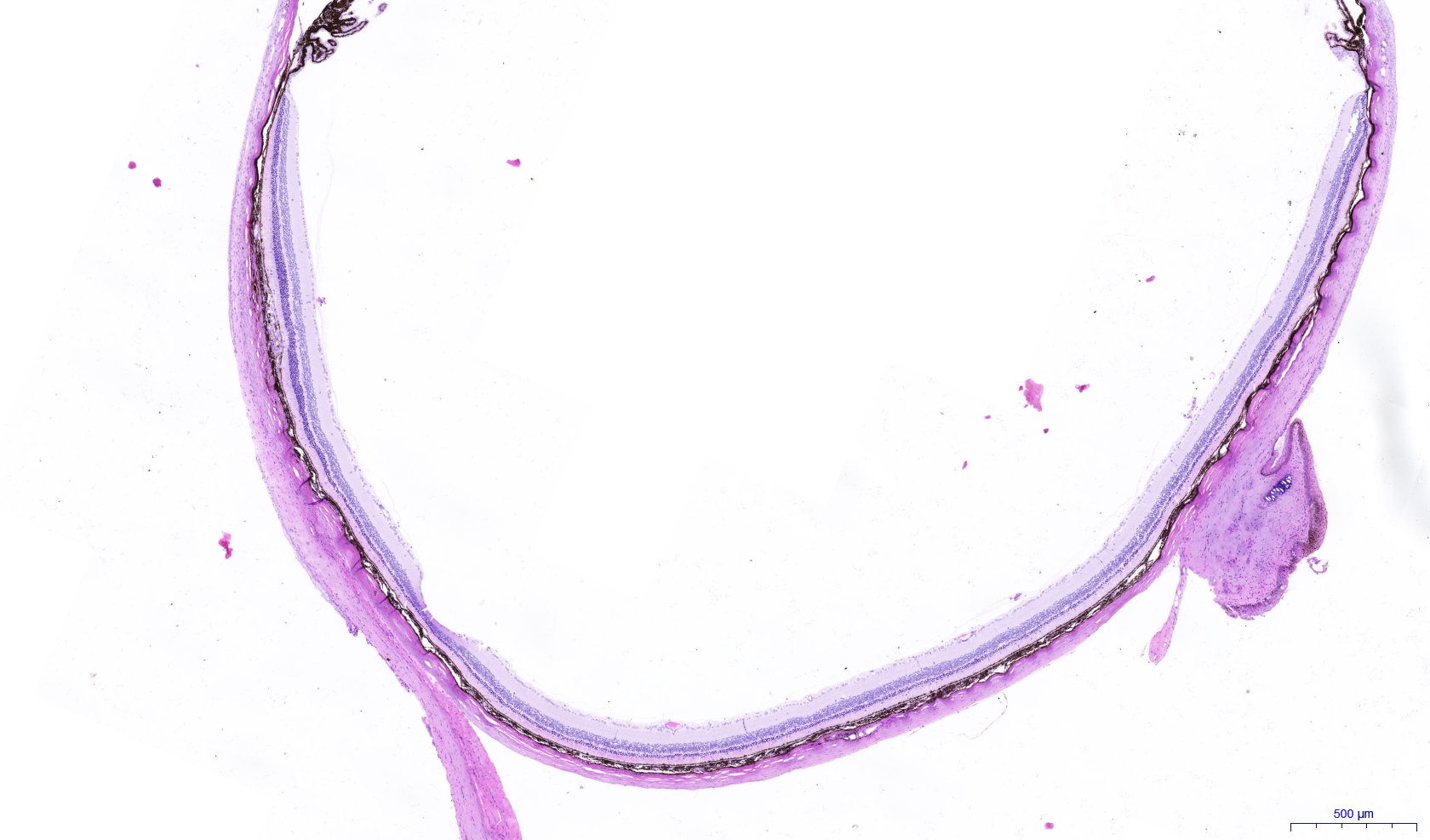

Supplement: Supplementary file 3 [file Data_Sheet_1.ZIP › raw data 2/figure5E.jpg]

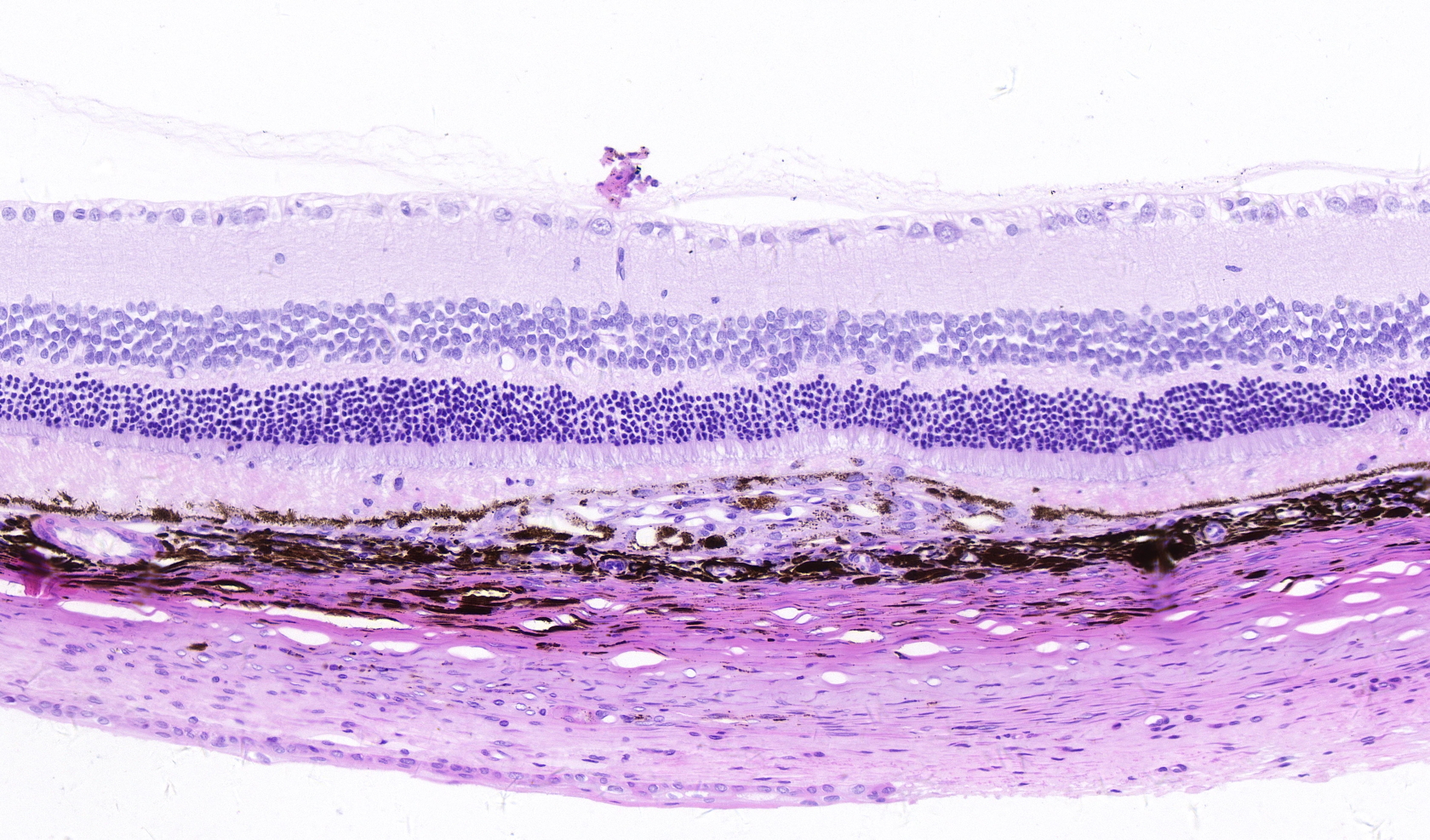

Supplement: Supplementary file 3 [file Data_Sheet_1.ZIP › raw data 2/figure5F-1.jpg]

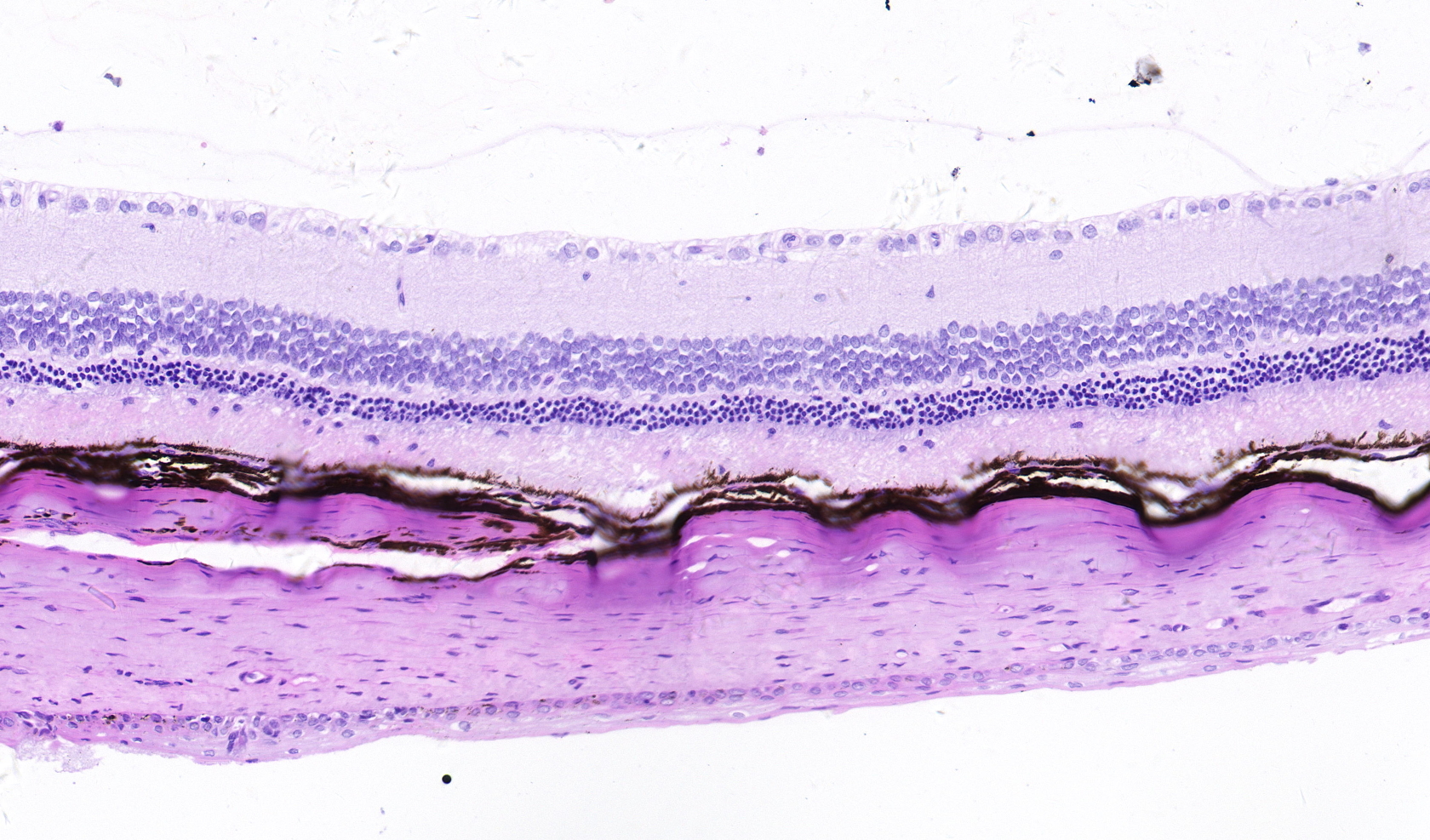

Supplement: Supplementary file 3 [file Data_Sheet_1.ZIP › raw data 2/figure5F-2.jpg]

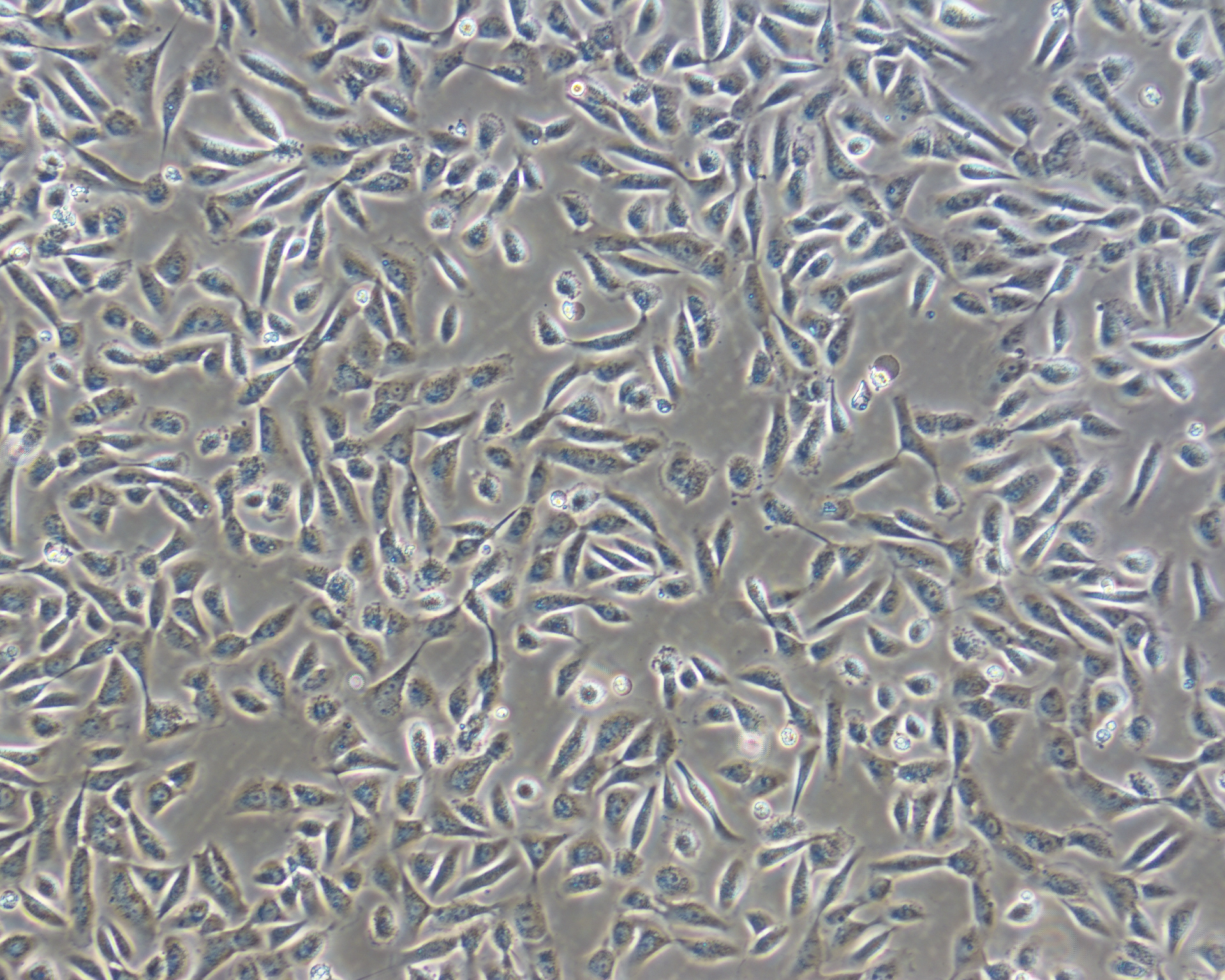

Supplement: Supplementary file 4 [file Data_Sheet_2.ZIP › raw data 1/figure1A.jpg]

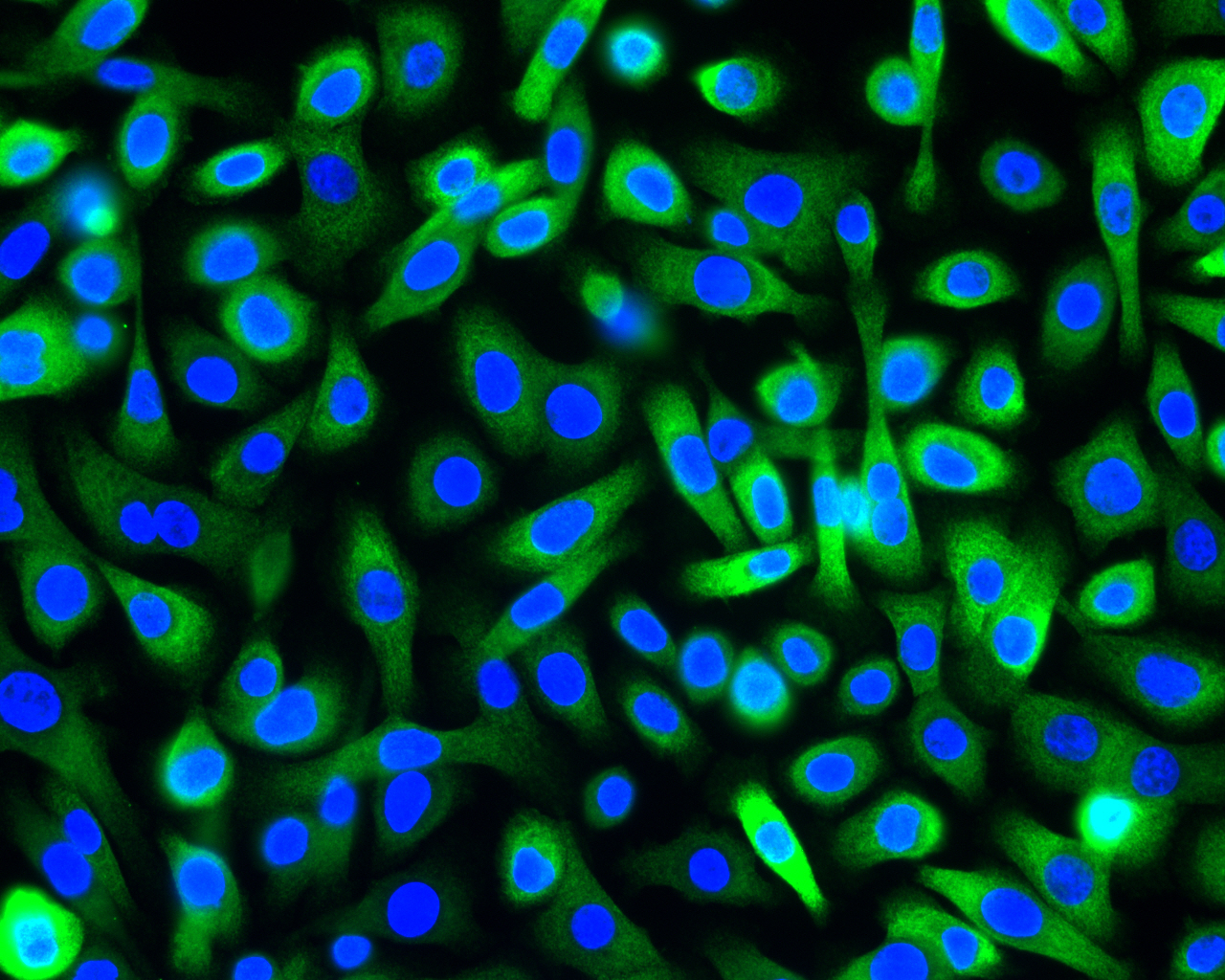

Supplement: Supplementary file 4 [file Data_Sheet_2.ZIP › raw data 1/figure1B.jpg]

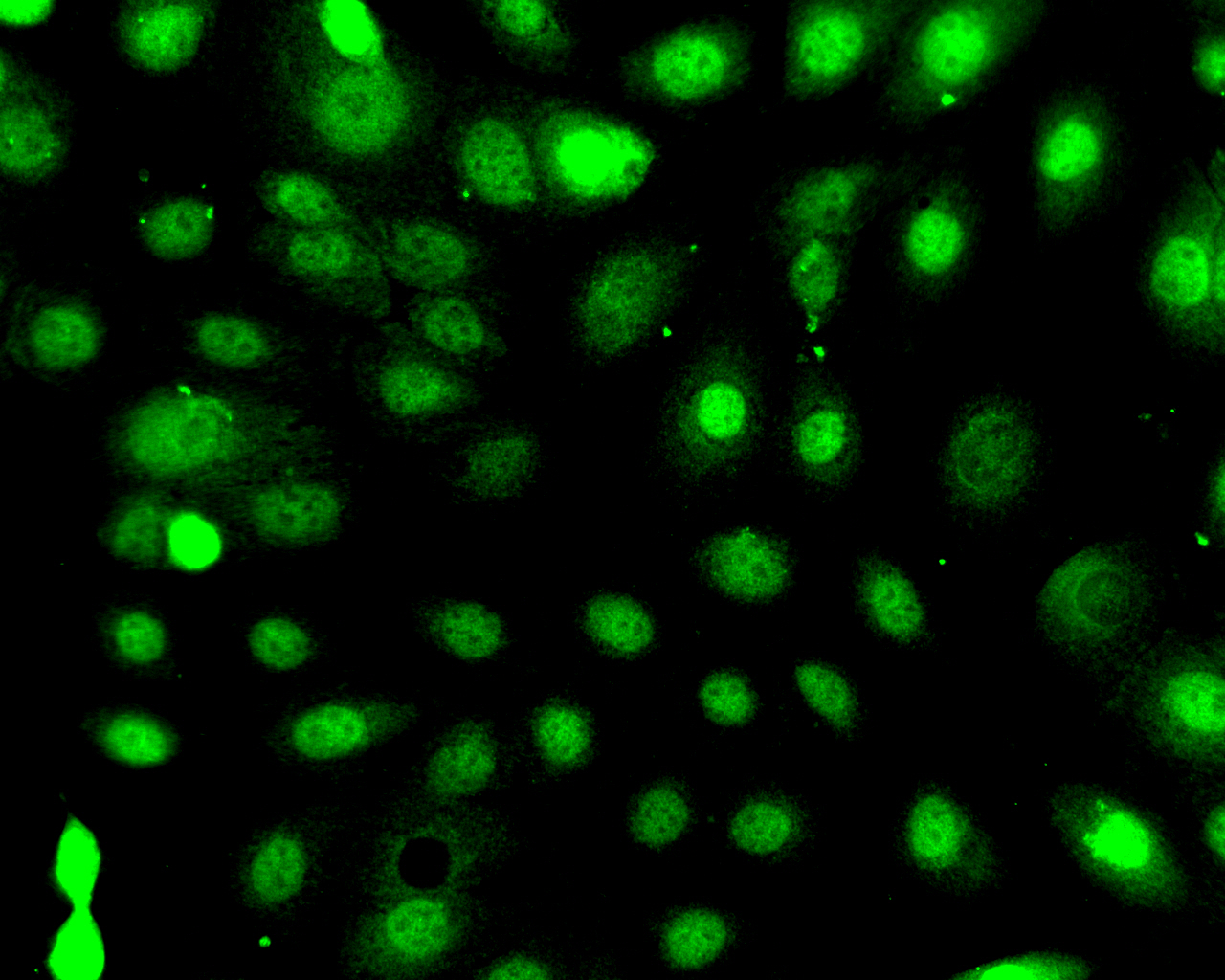

Supplement: Supplementary file 4 [file Data_Sheet_2.ZIP › raw data 1/figure1I.jpg]

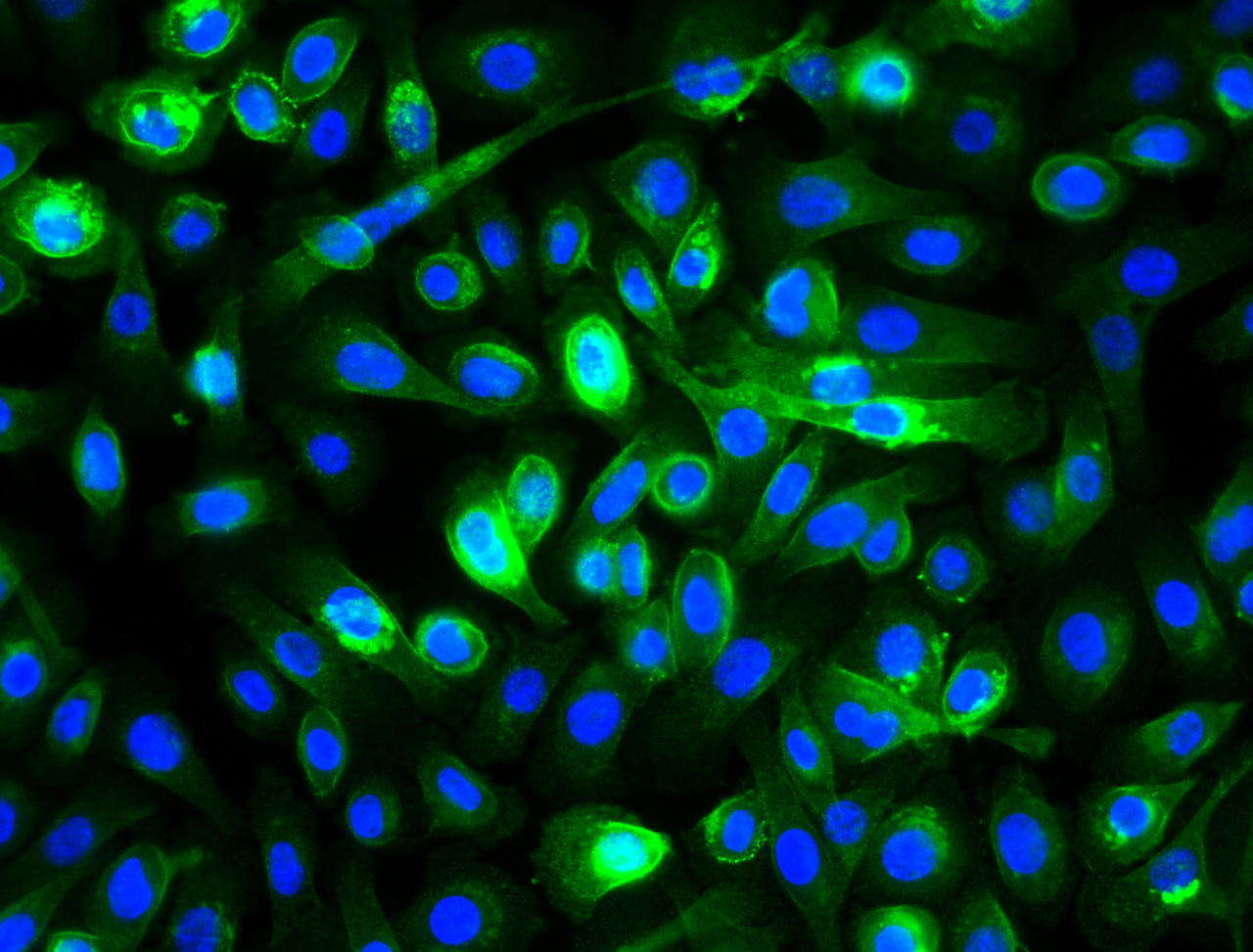

Supplement: Supplementary file 4 [file Data_Sheet_2.ZIP › raw data 1/figure1J.jpg]

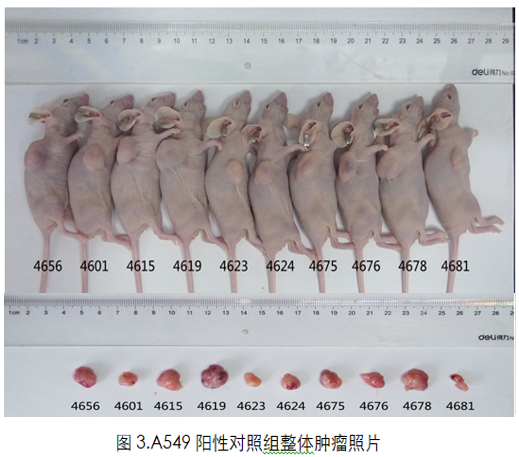

Supplement: Supplementary file 4 [file Data_Sheet_2.ZIP › raw data 1/figure1L-2.png]

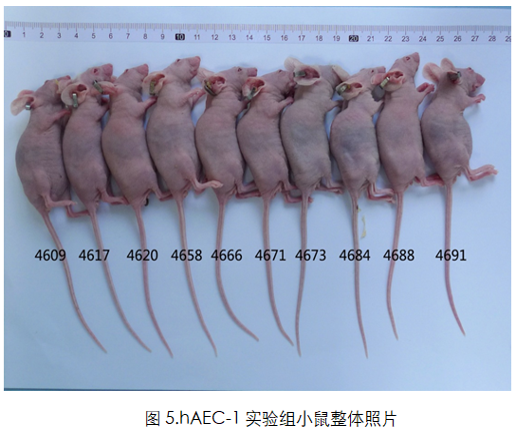

Supplement: Supplementary file 4 [file Data_Sheet_2.ZIP › raw data 1/figure1L.png]

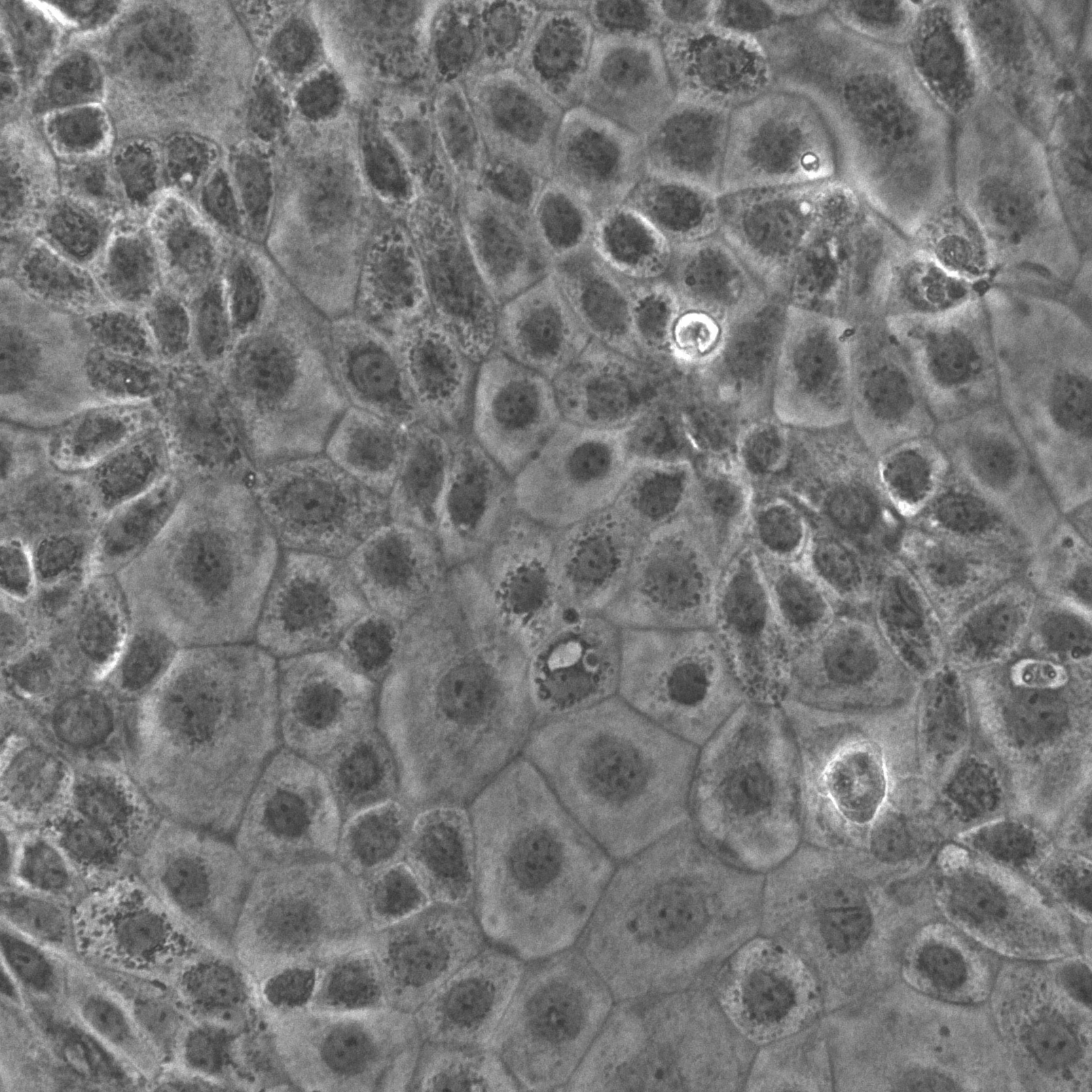

Supplement: Supplementary file 4 [file Data_Sheet_2.ZIP › raw data 1/figure3A.tif]

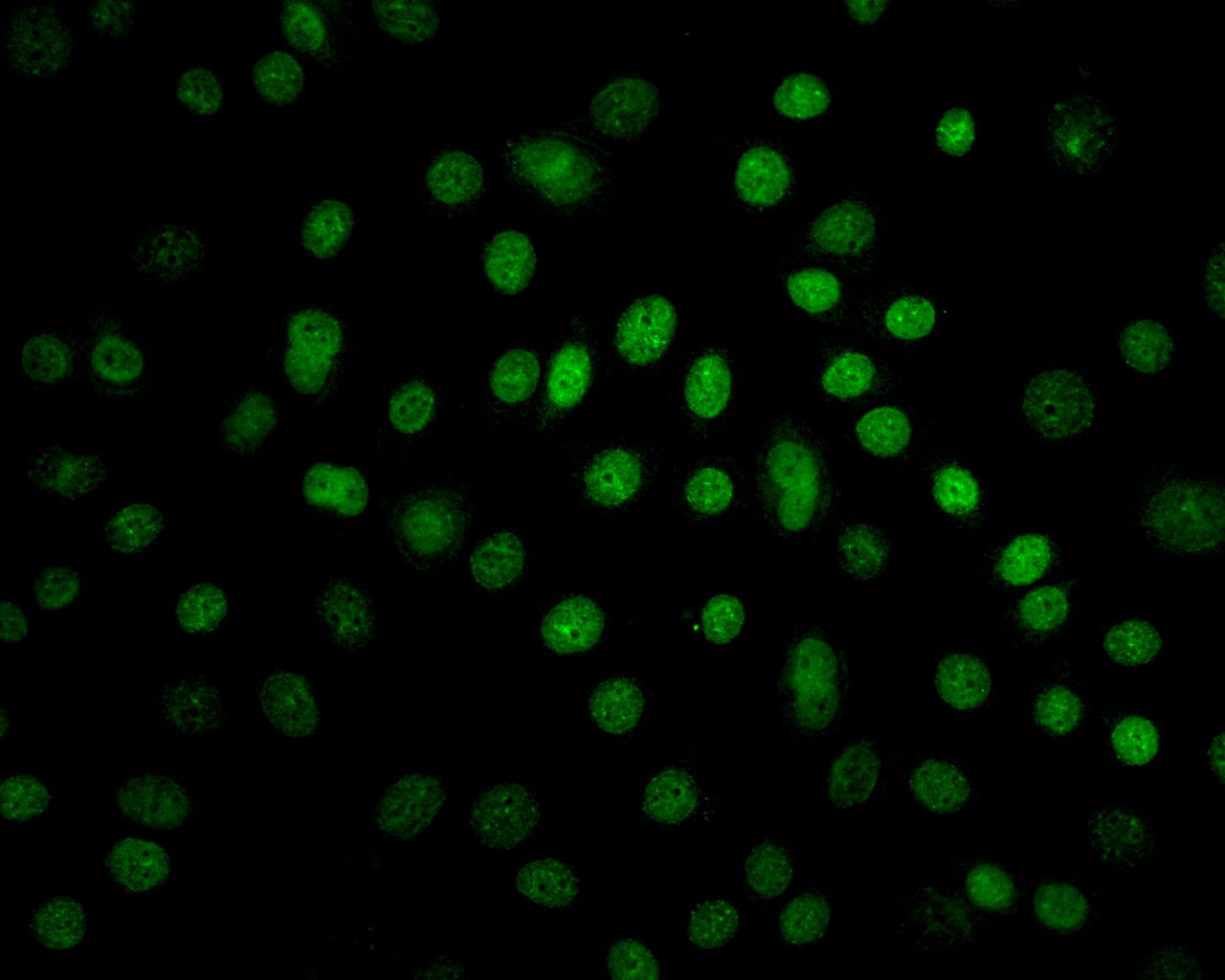

Supplement: Supplementary file 4 [file Data_Sheet_2.ZIP › raw data 1/figure3B.jpg]

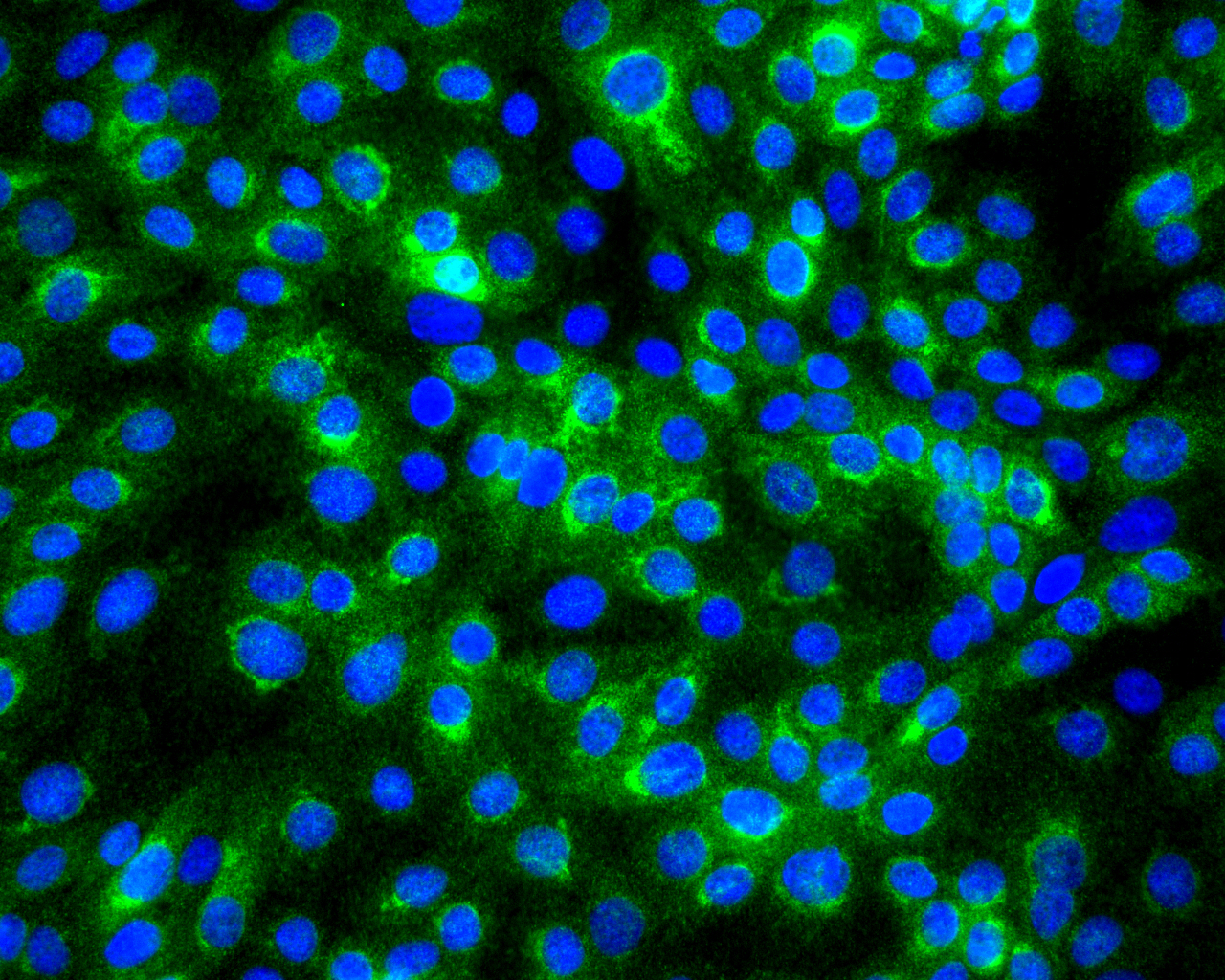

Supplement: Supplementary file 4 [file Data_Sheet_2.ZIP › raw data 1/figure3C.jpg]

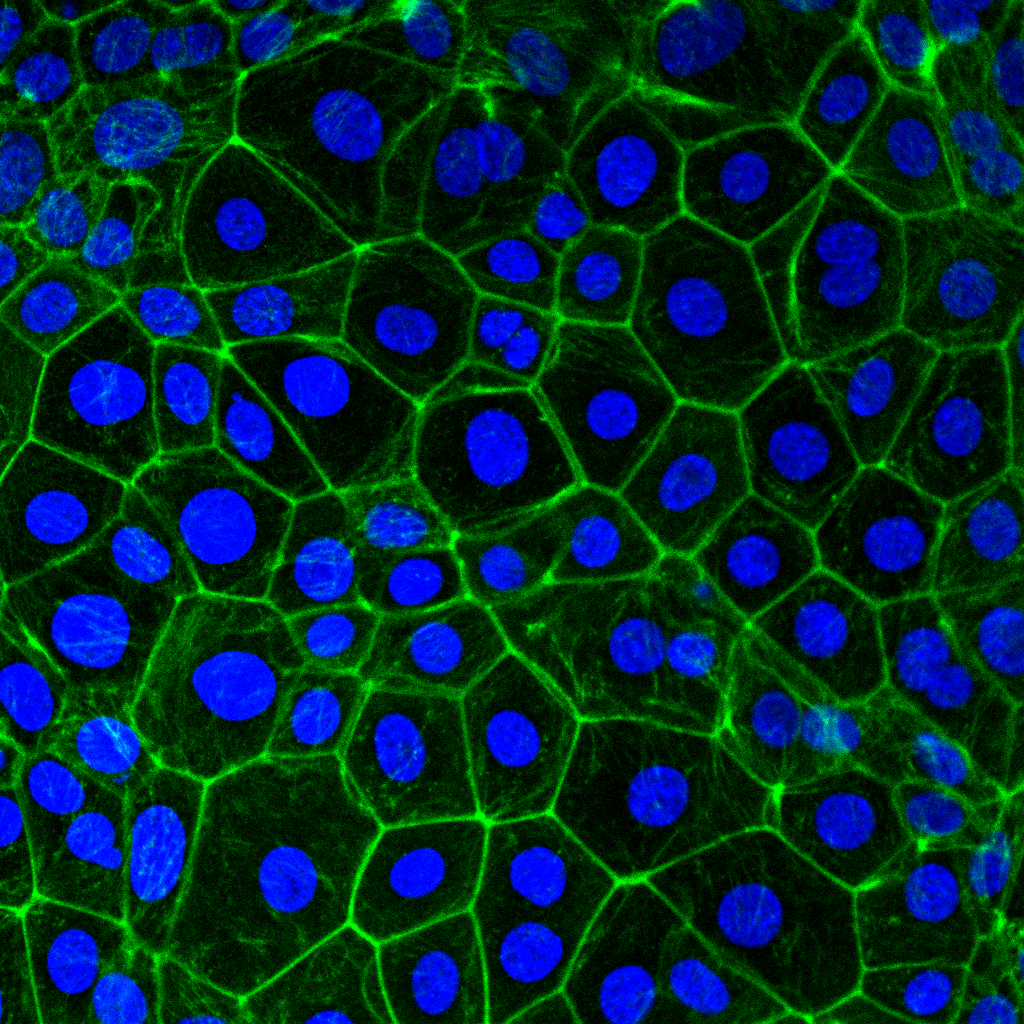

Supplement: Supplementary file 4 [file Data_Sheet_2.ZIP › raw data 1/figure3D.tif]

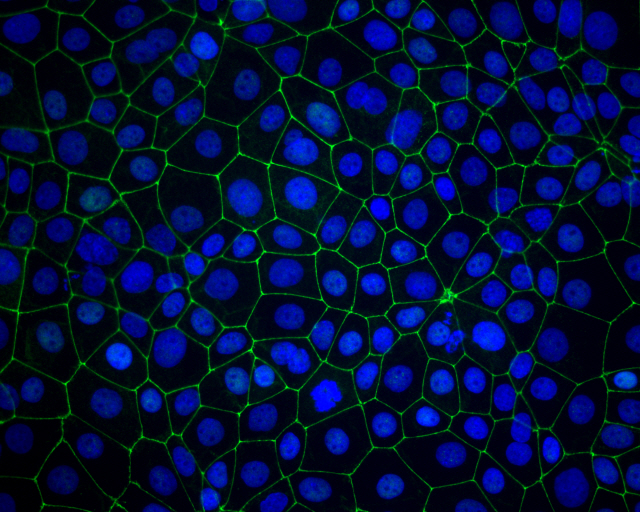

Supplement: Supplementary file 4 [file Data_Sheet_2.ZIP › raw data 1/figure3E.jpg]

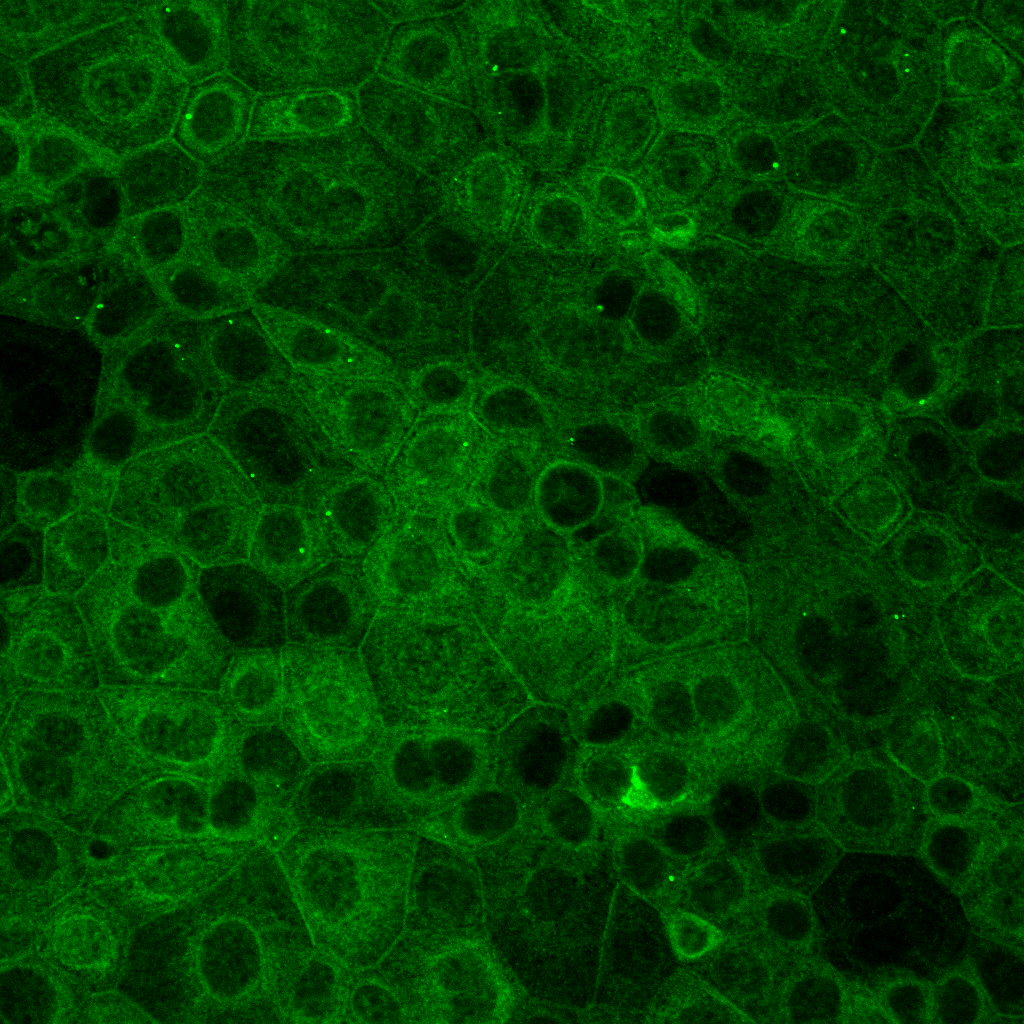

Supplement: Supplementary file 4 [file Data_Sheet_2.ZIP › raw data 1/figure3F.tif]

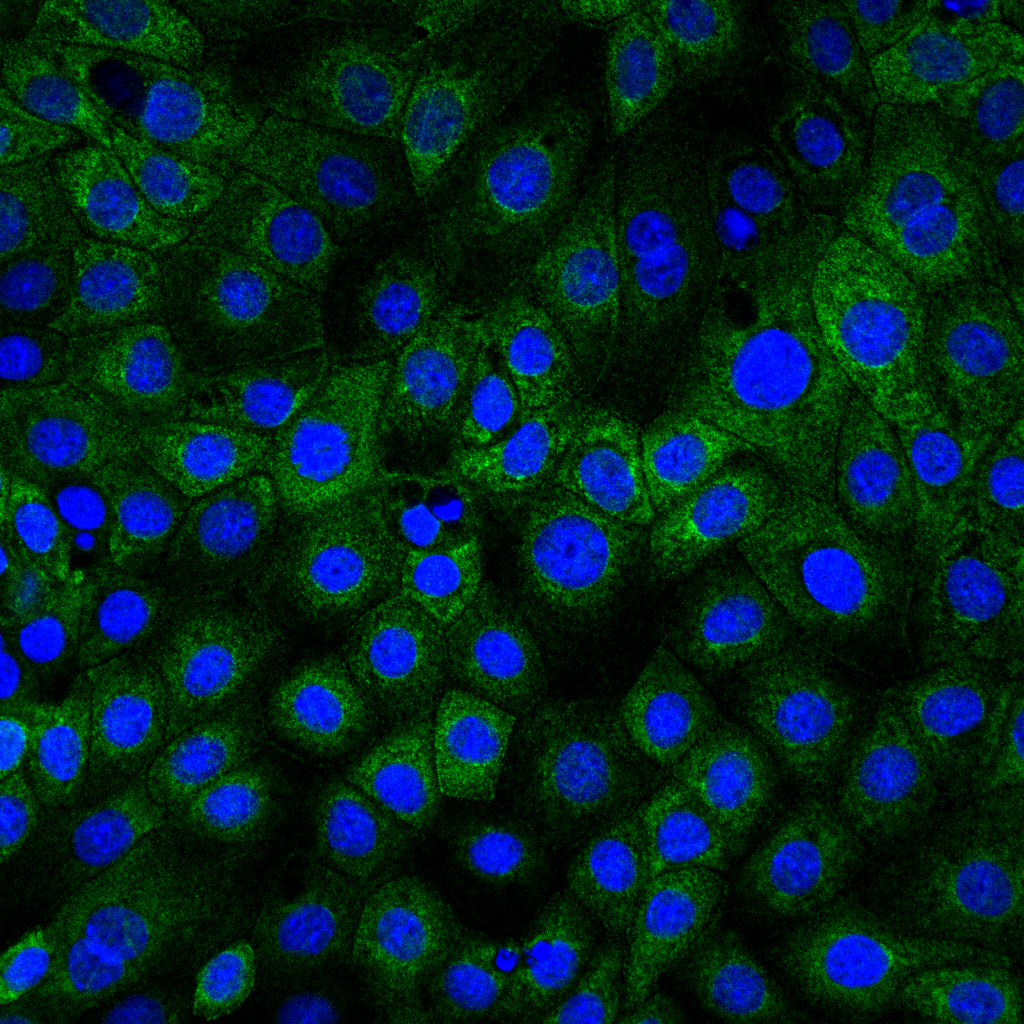

Supplement: Supplementary file 4 [file Data_Sheet_2.ZIP › raw data 1/figure3G.tif]

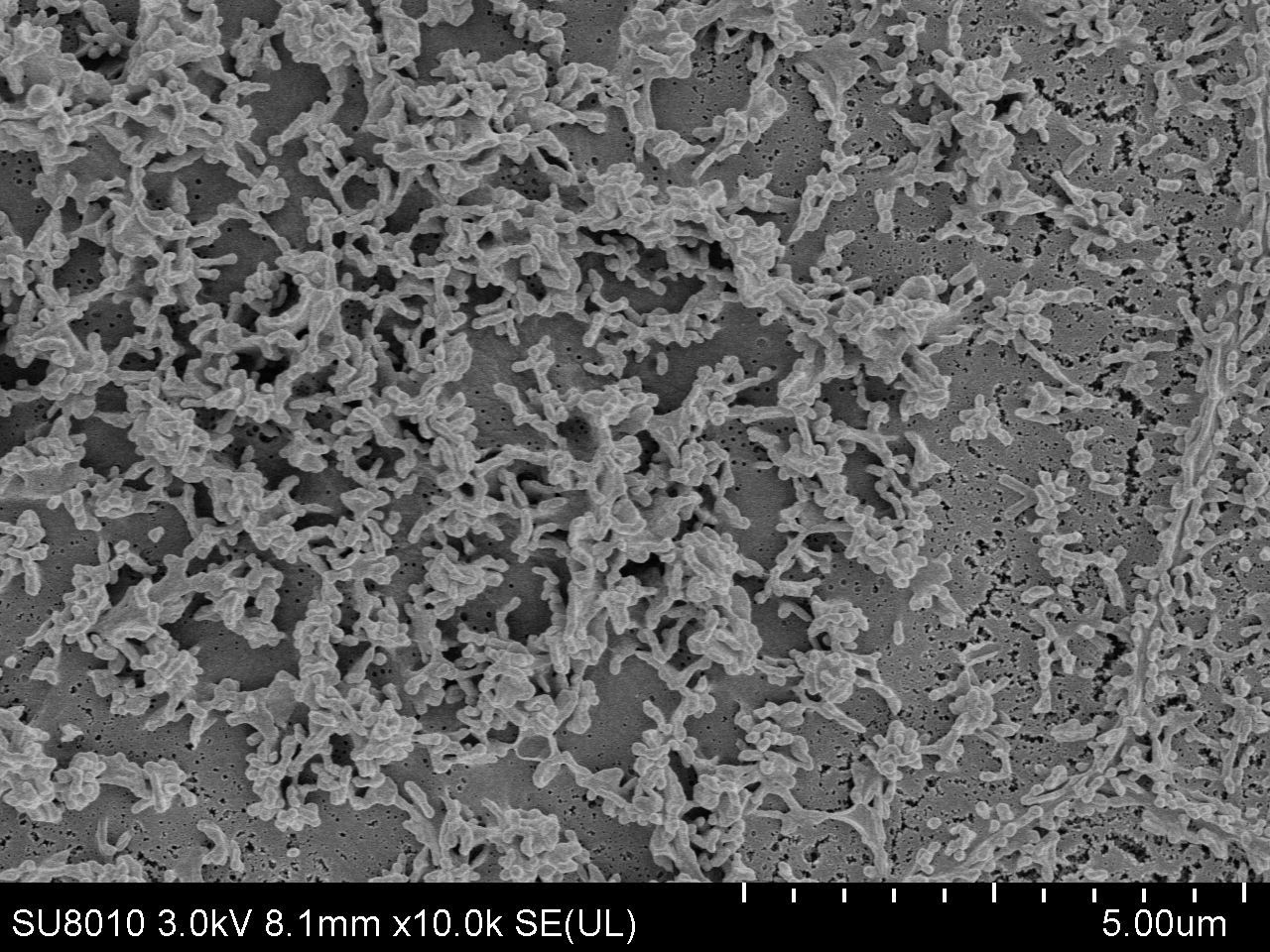

Supplement: Supplementary file 4 [file Data_Sheet_2.ZIP › raw data 1/figure3I.tif]

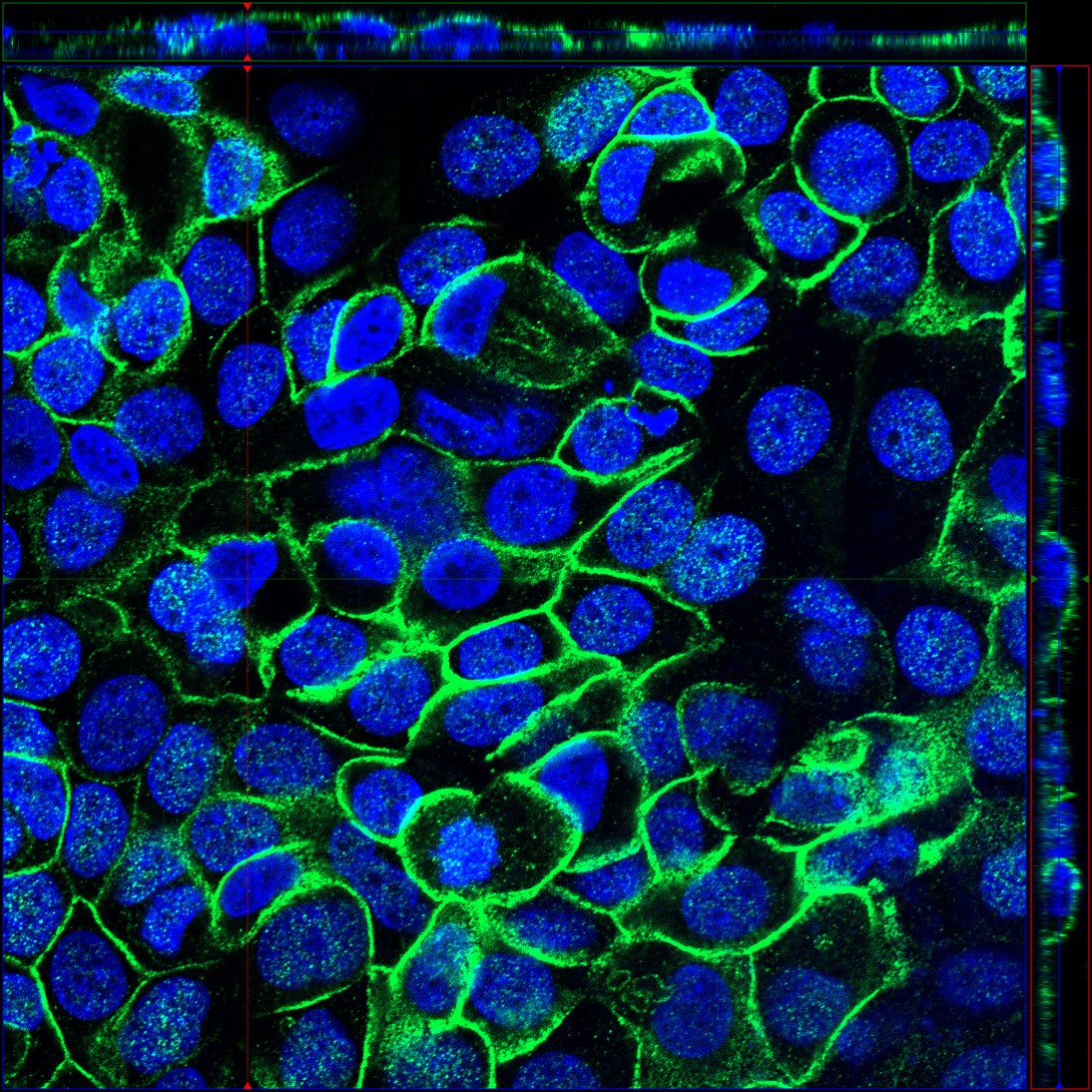

Supplement: Supplementary file 4 [file Data_Sheet_2.ZIP › raw data 1/figure3L.jpg]

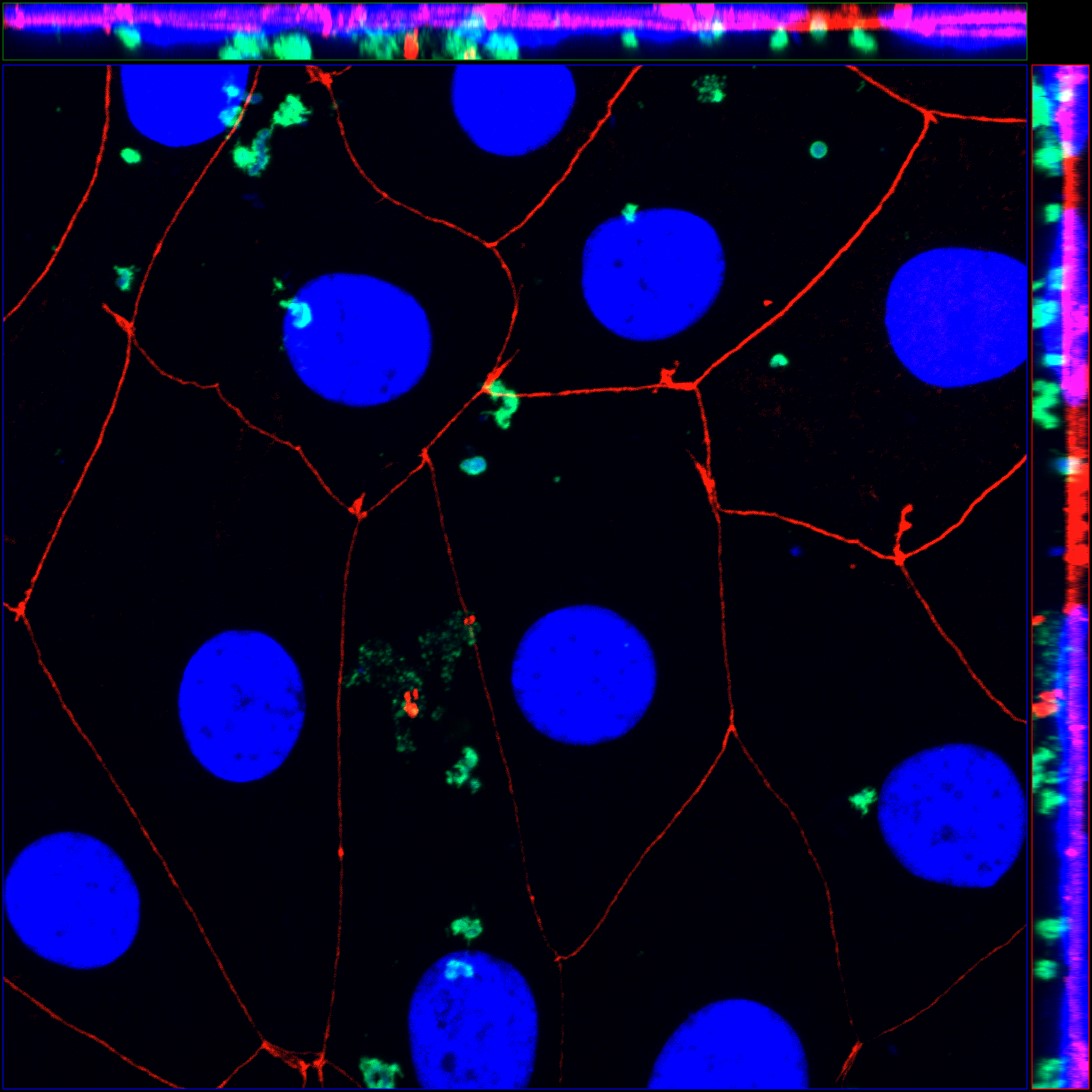

Supplement: Supplementary file 4 [file Data_Sheet_2.ZIP › raw data 1/figure3M.jpg]

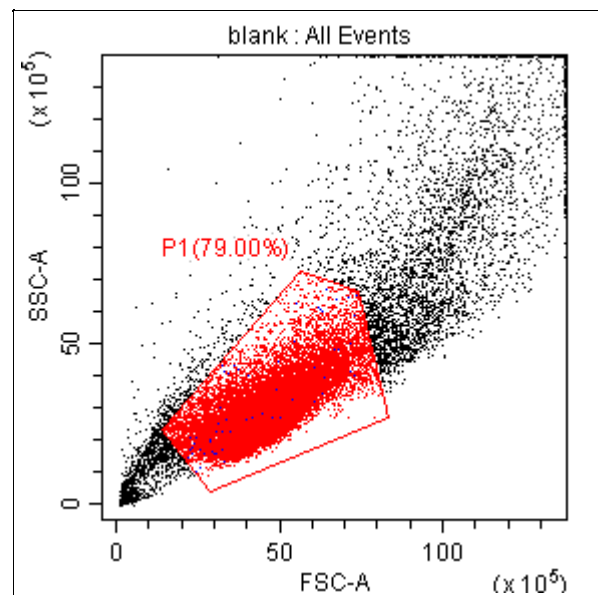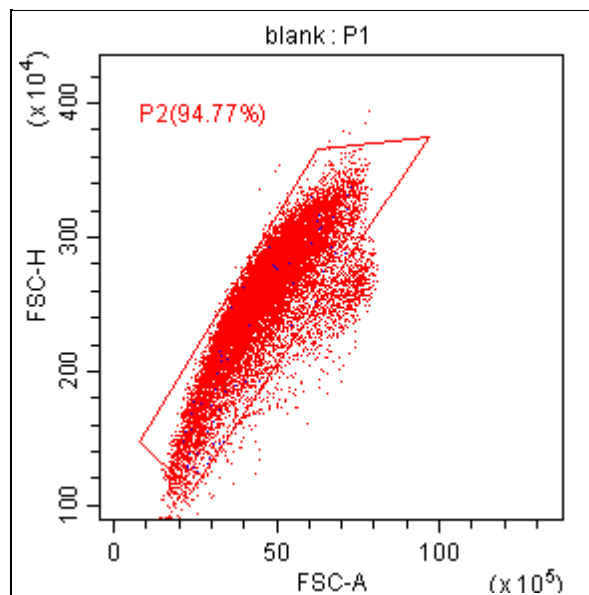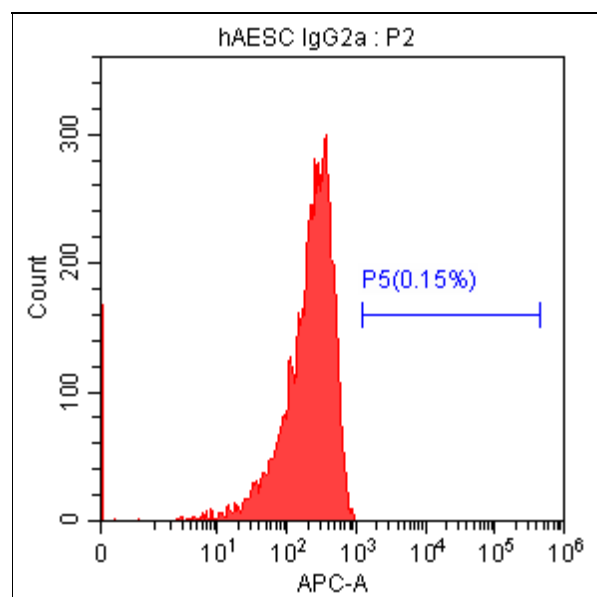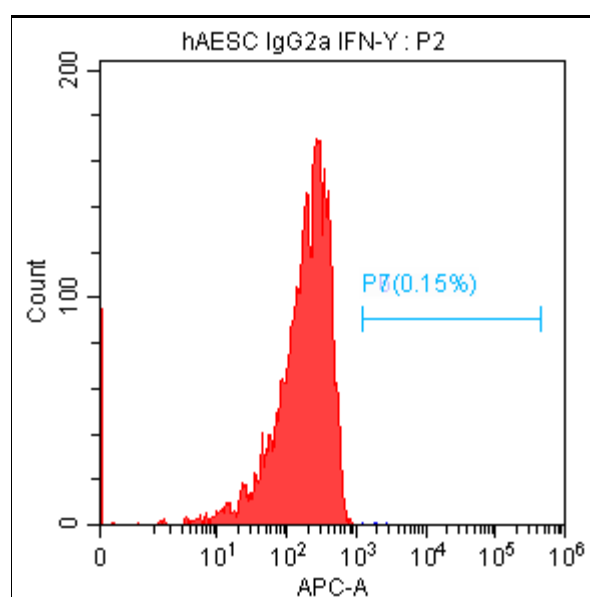

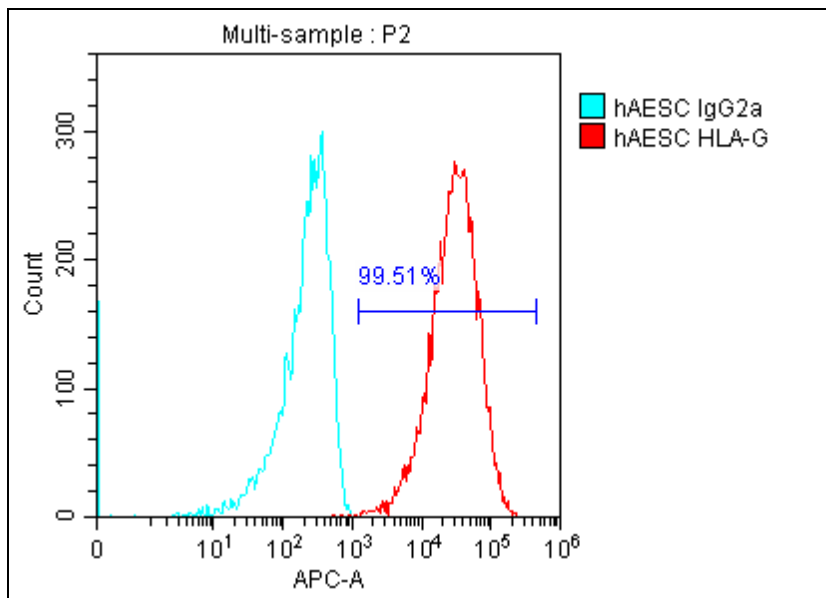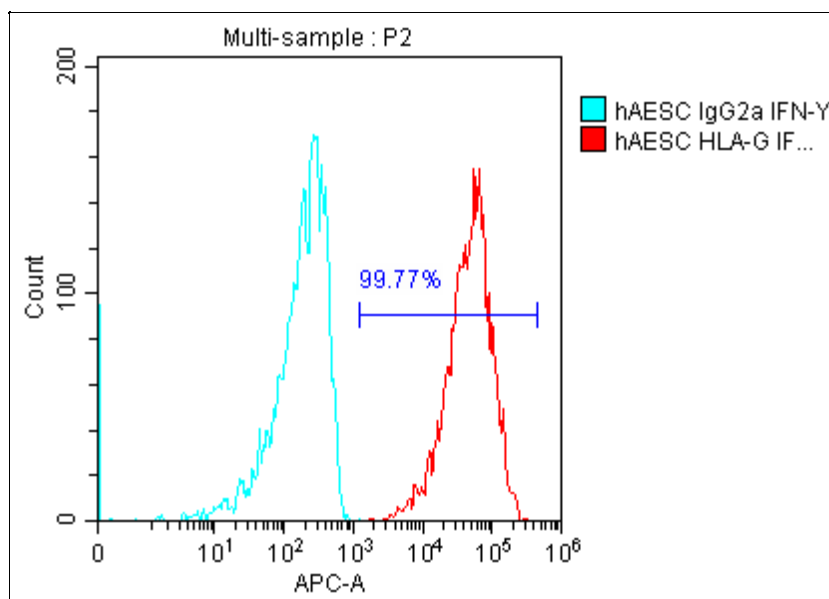

Supplement: Supplementary file 4 [file Data_Sheet_2.ZIP › raw data 1/figure4 E HLA-G.pdf]

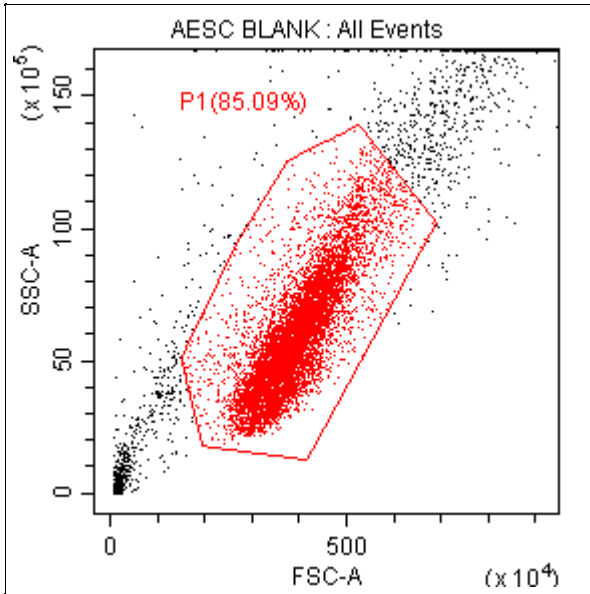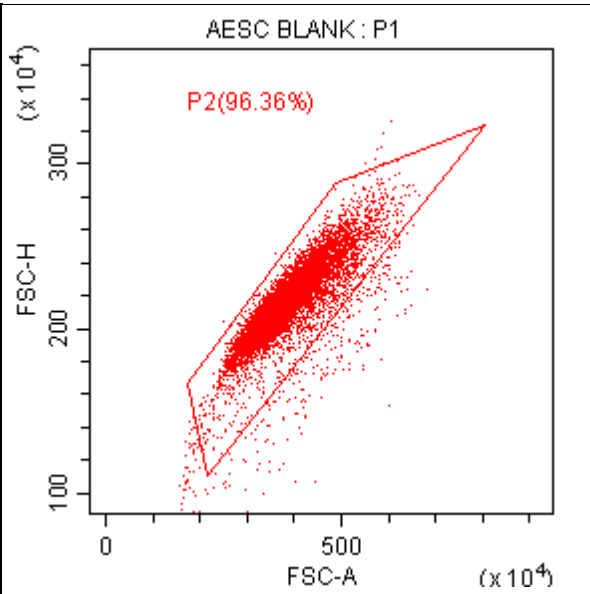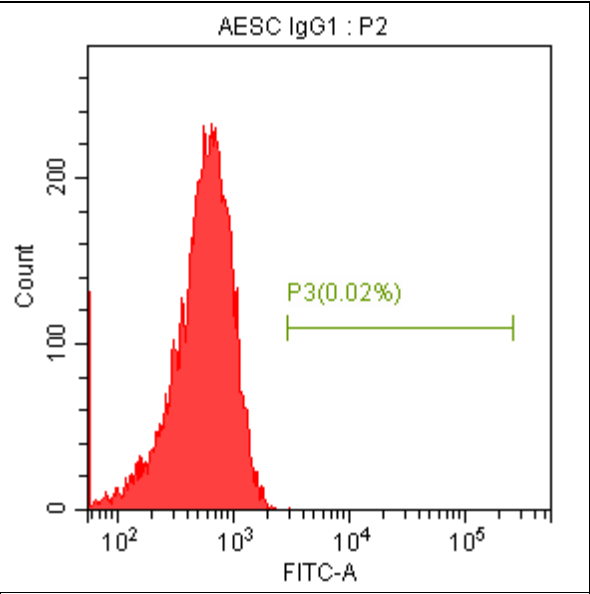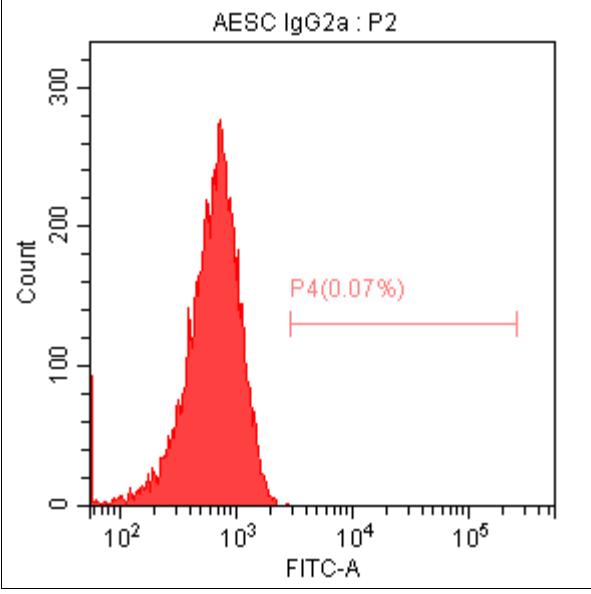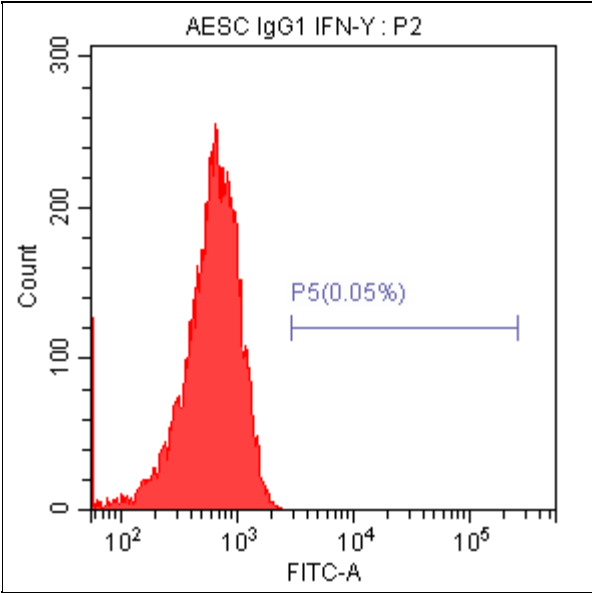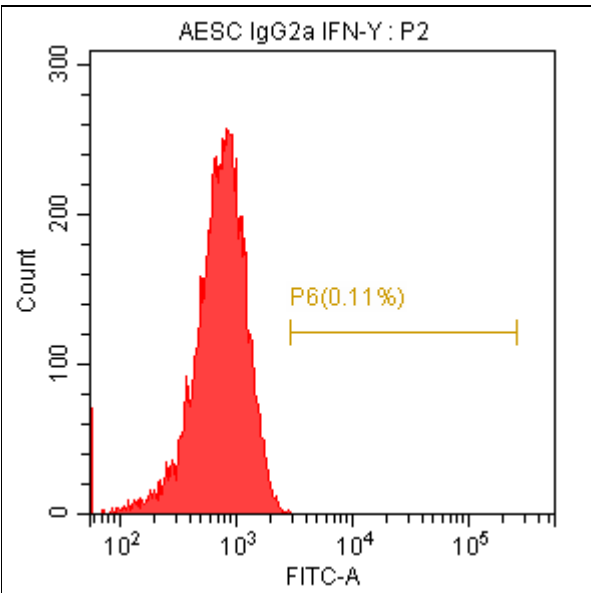

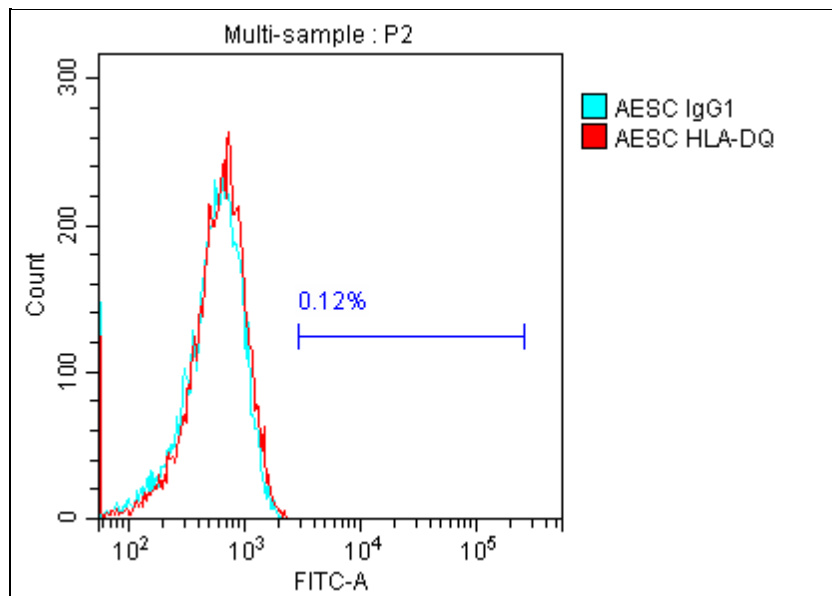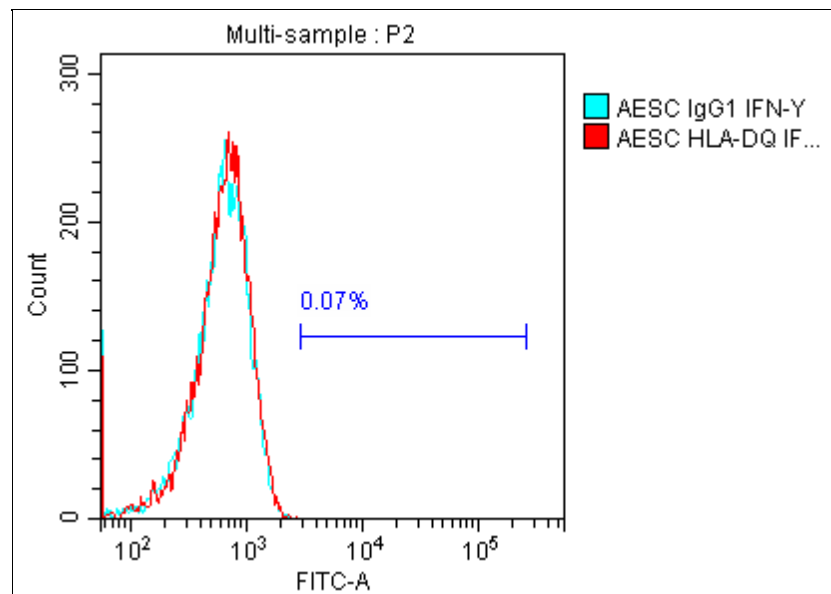

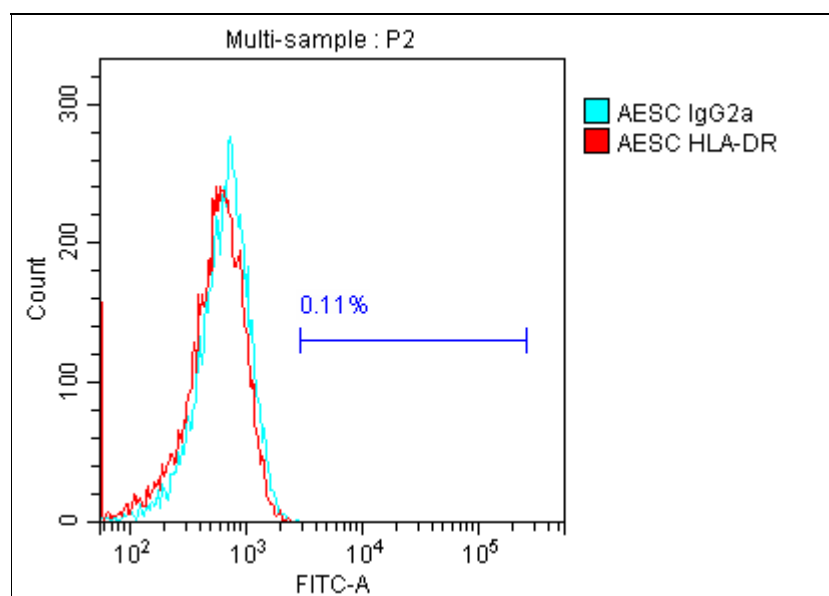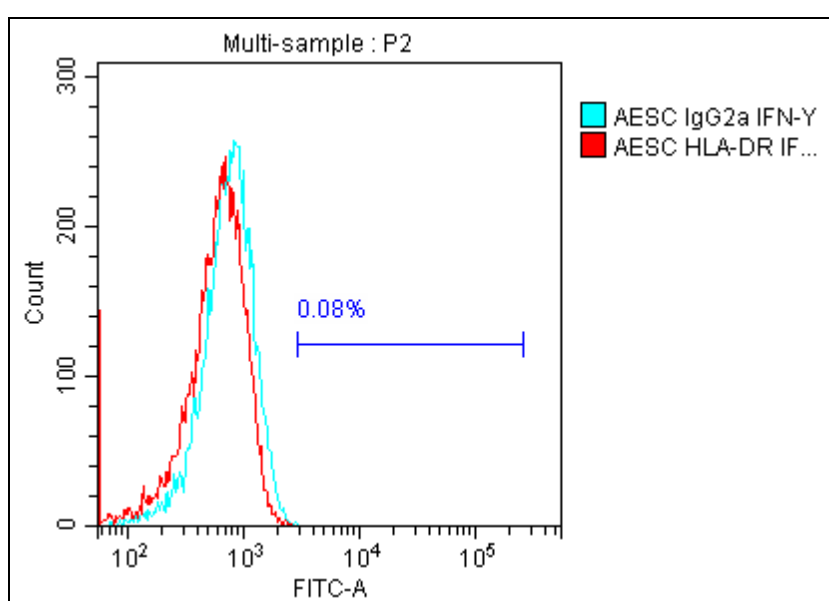

Supplement: Supplementary file 4 [file Data_Sheet_2.ZIP › raw data 1/figure4 E HLADR HLADQ.pdf]

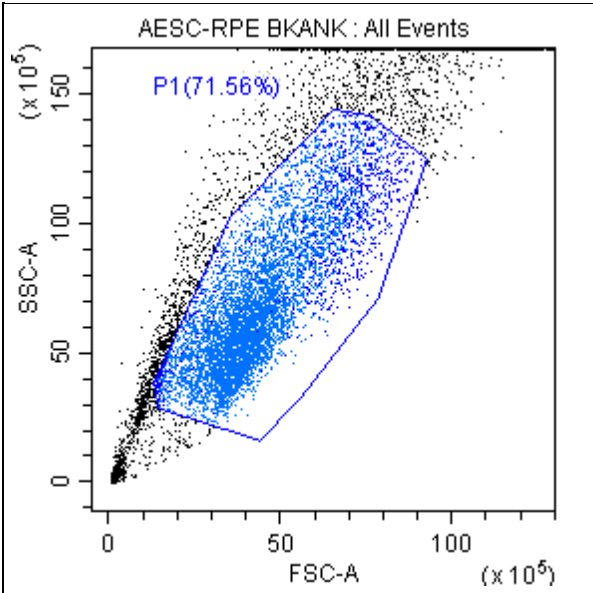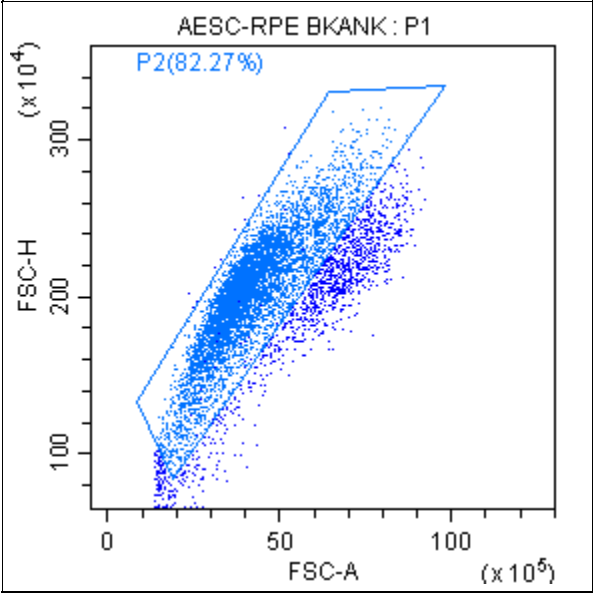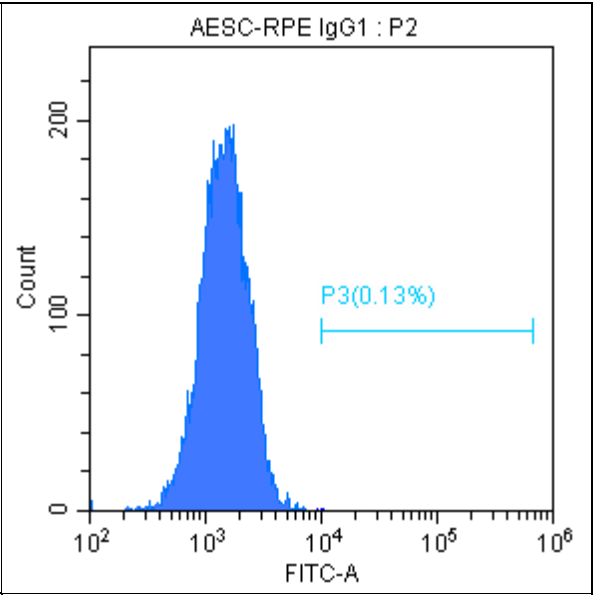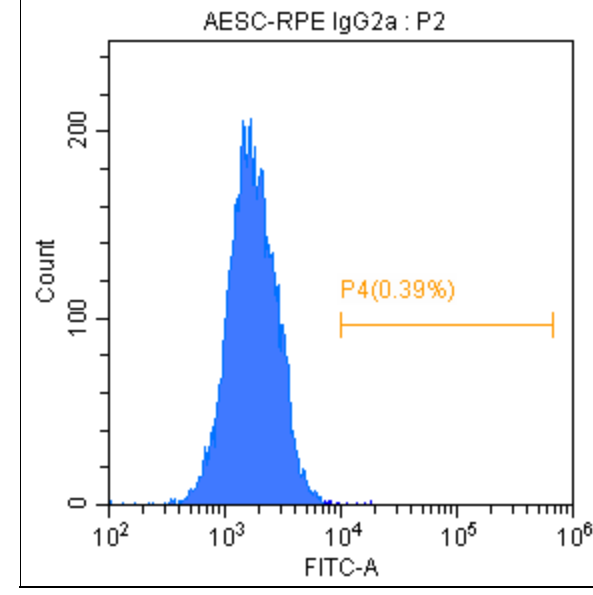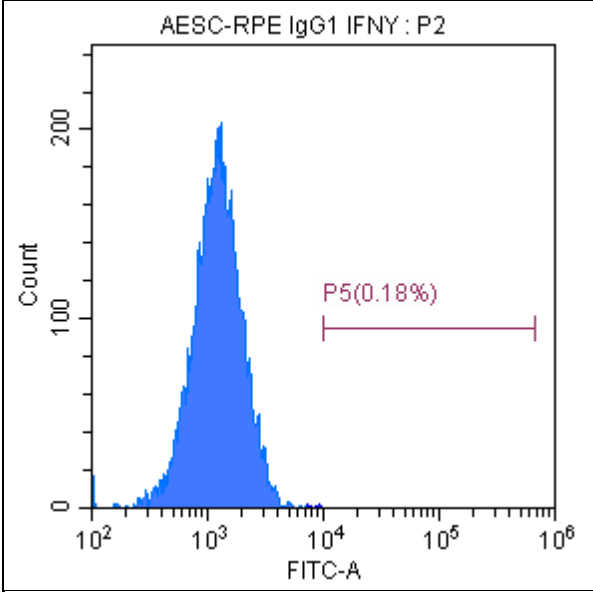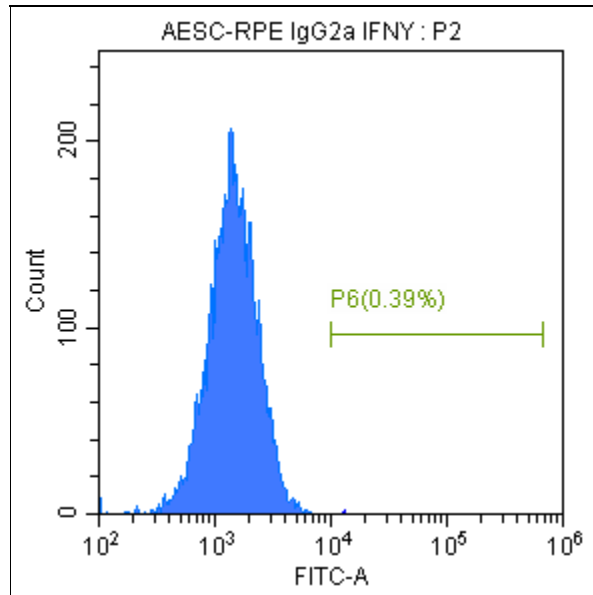

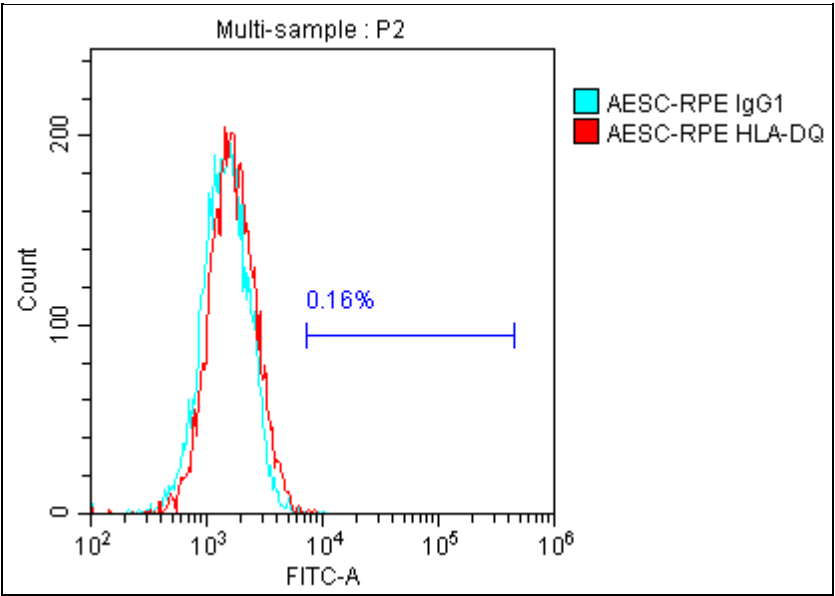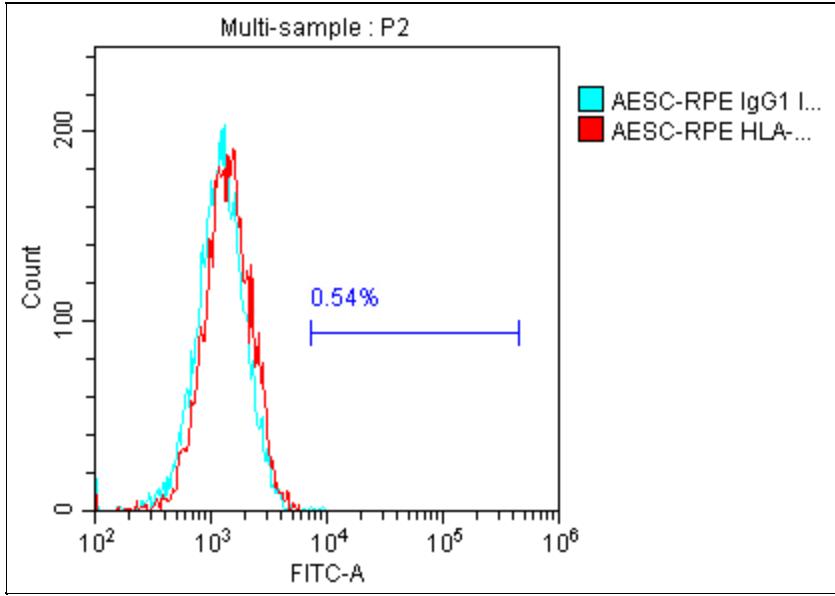

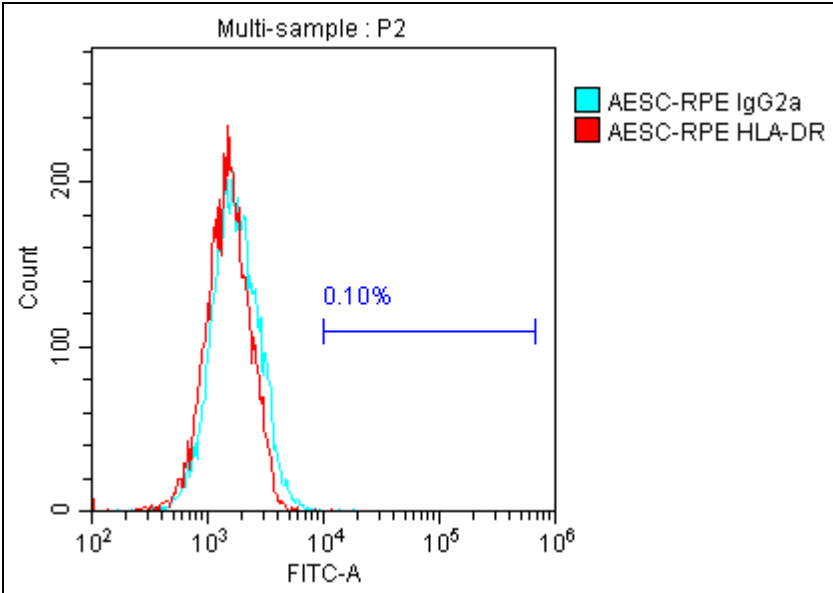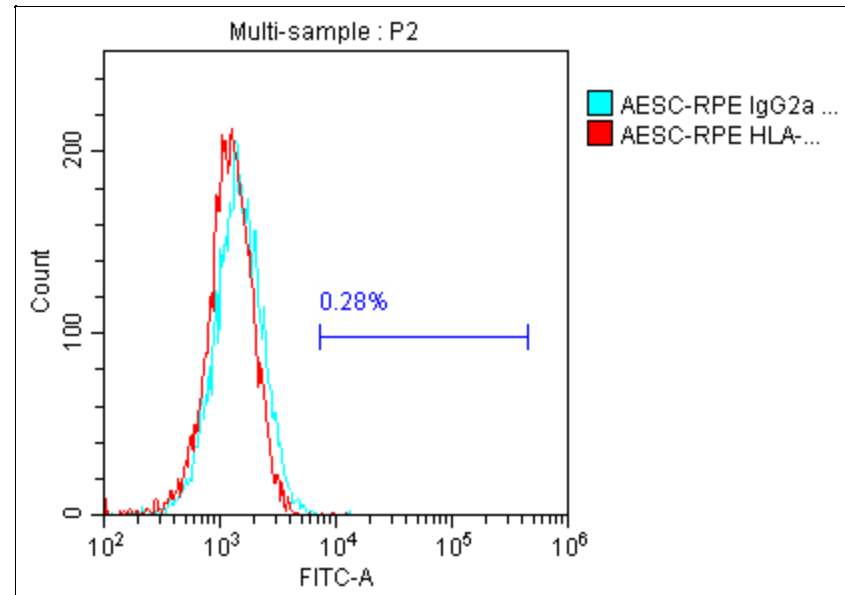

Supplement: Supplementary file 4 [file Data_Sheet_2.ZIP › raw data 1/figure4 G HLA-DQ DR.pdf]

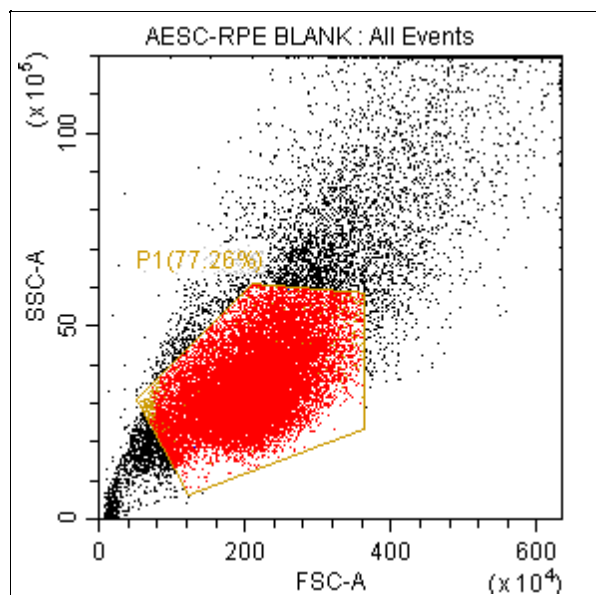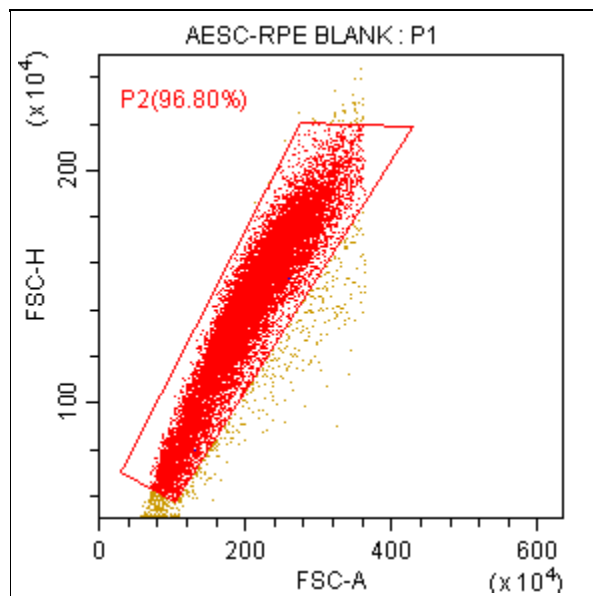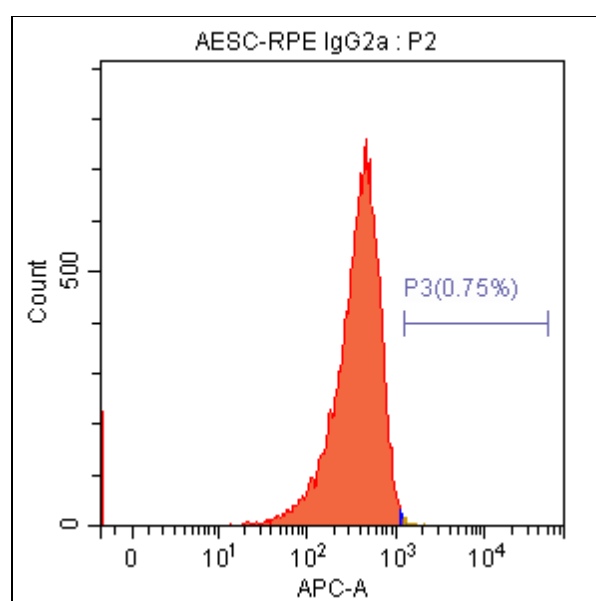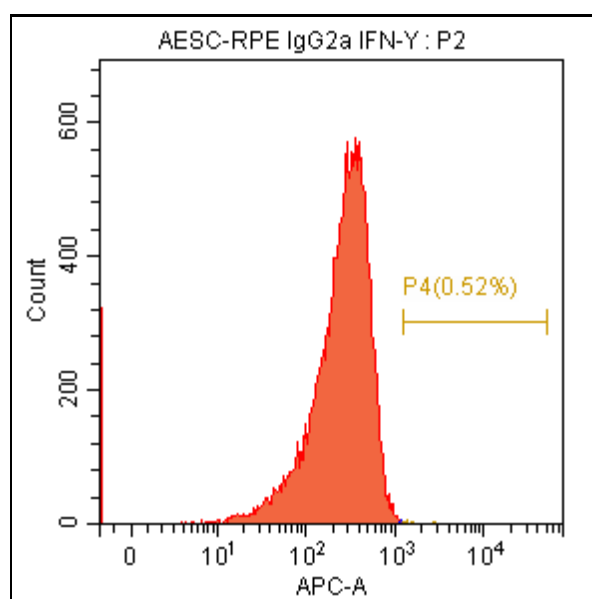

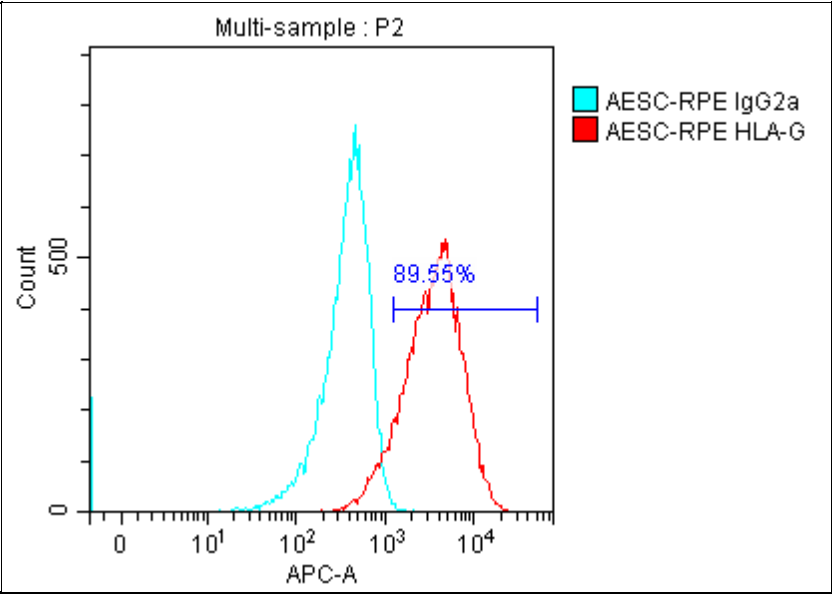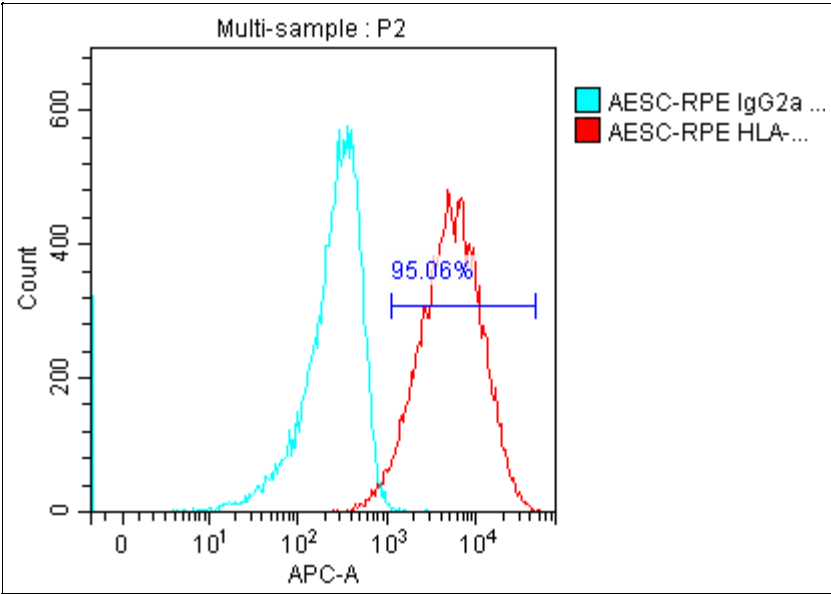

Supplement: Supplementary file 4 [file Data_Sheet_2.ZIP › raw data 1/figure4 G HLA-G.pdf]

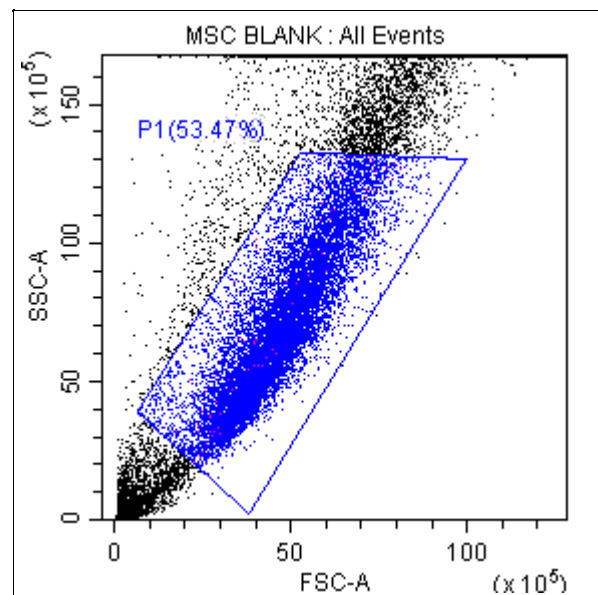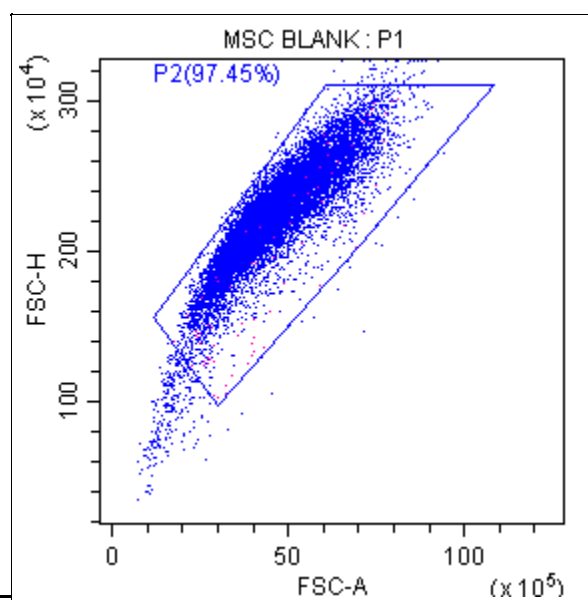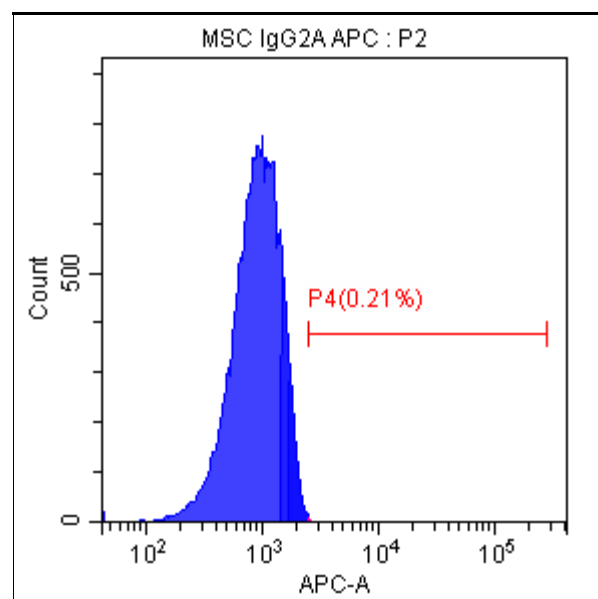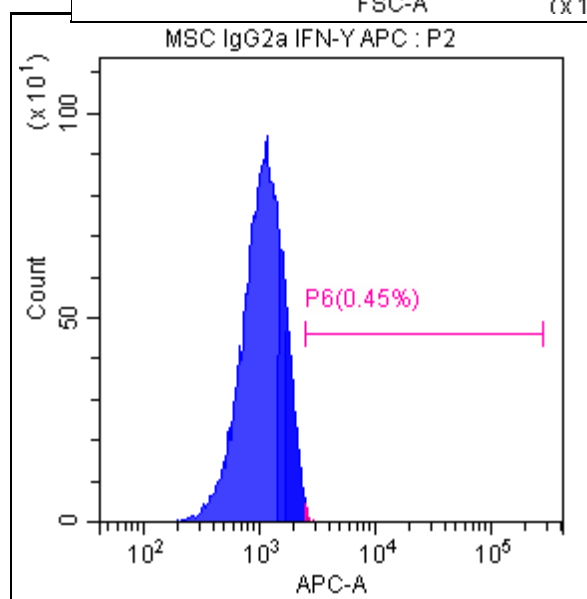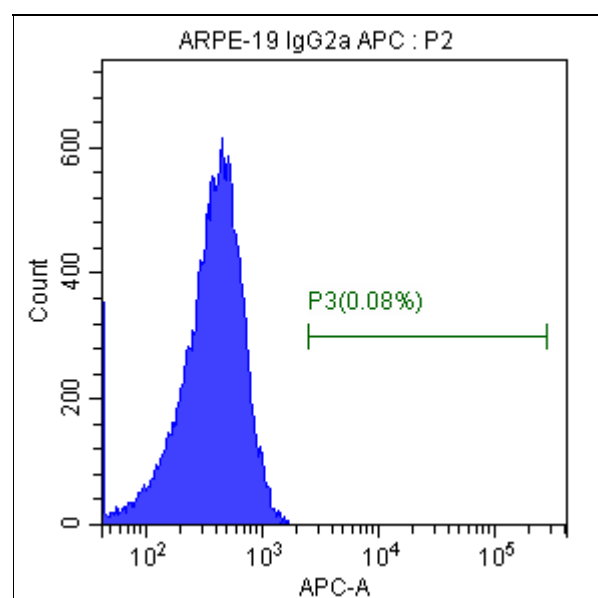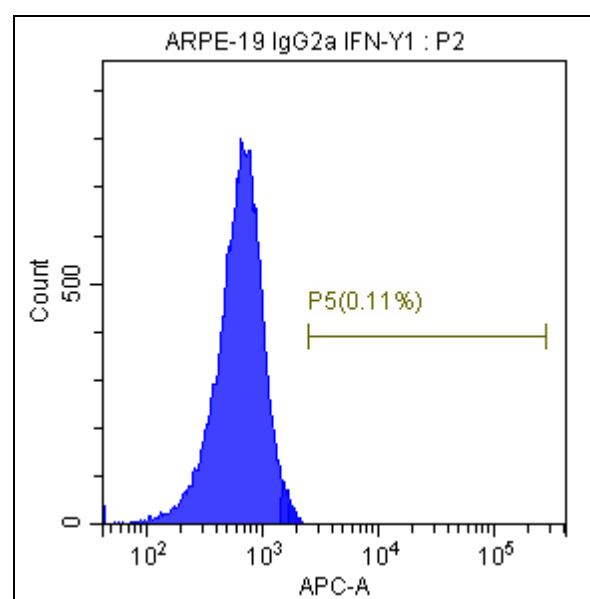

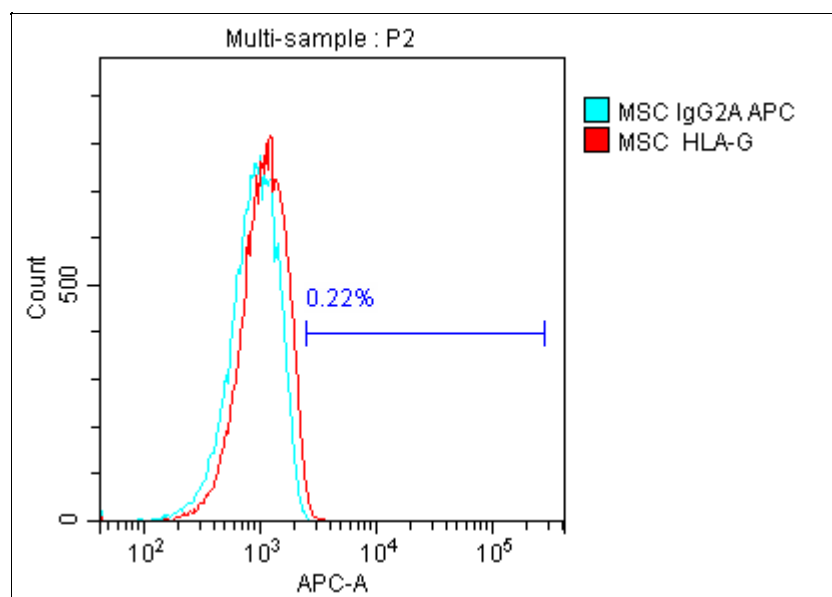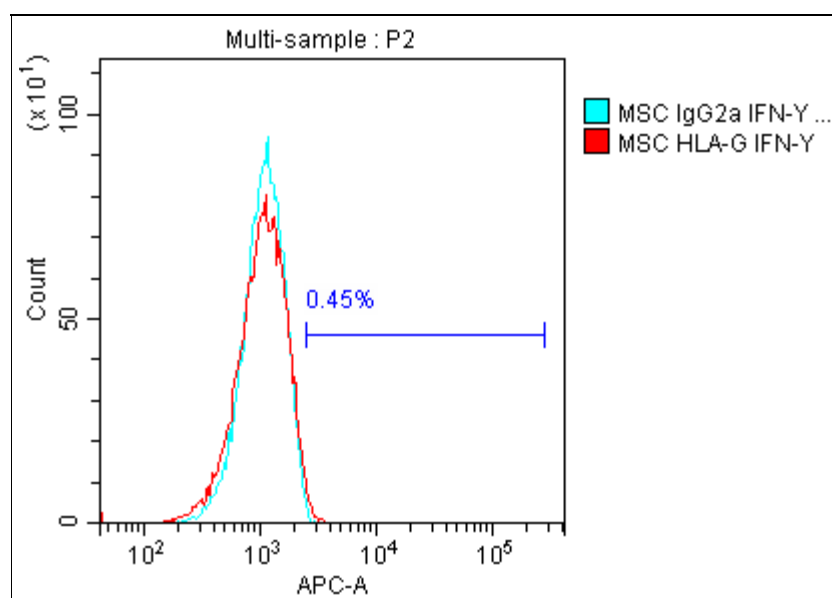

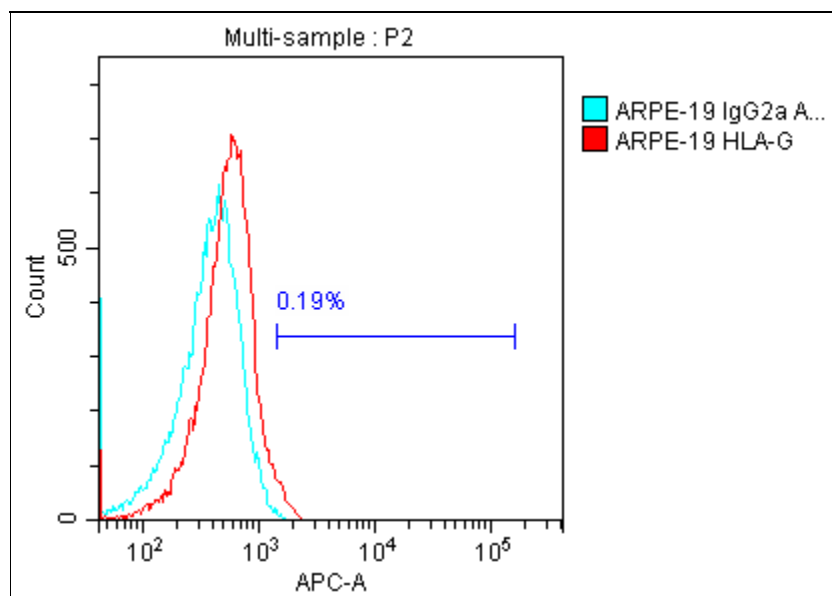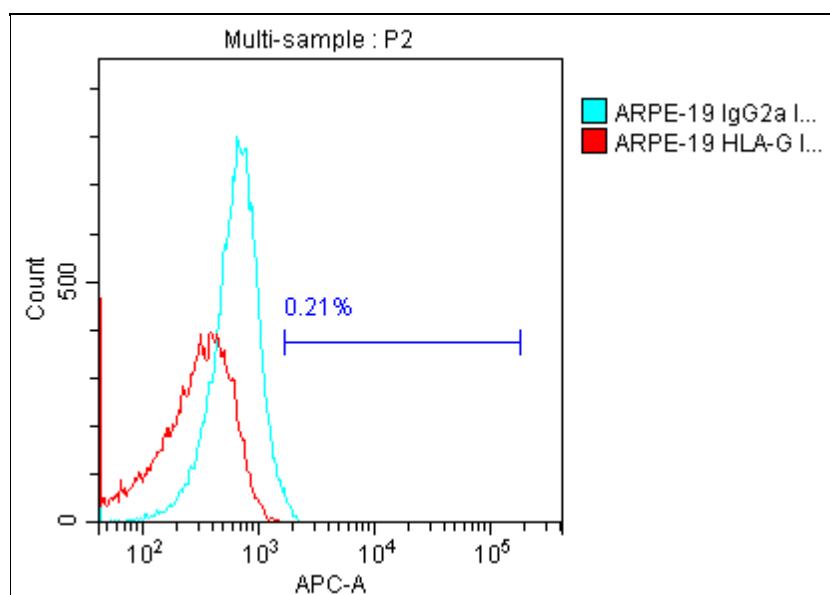

Supplement: Supplementary file 4 [file Data_Sheet_2.ZIP › raw data 1/figure4A HLA-G.pdf]

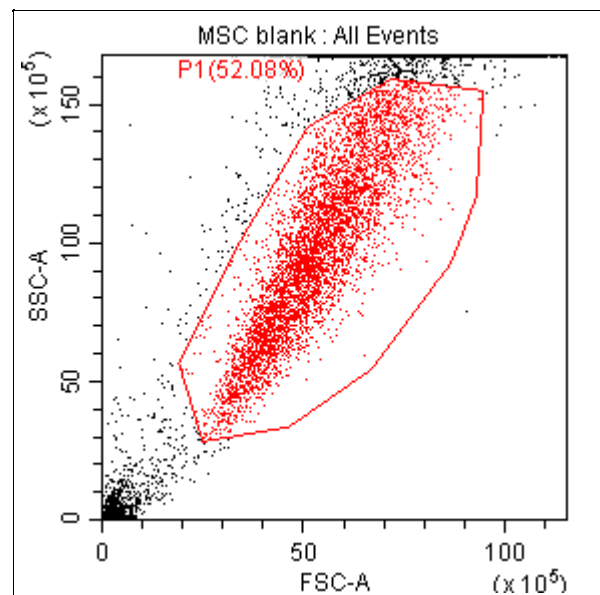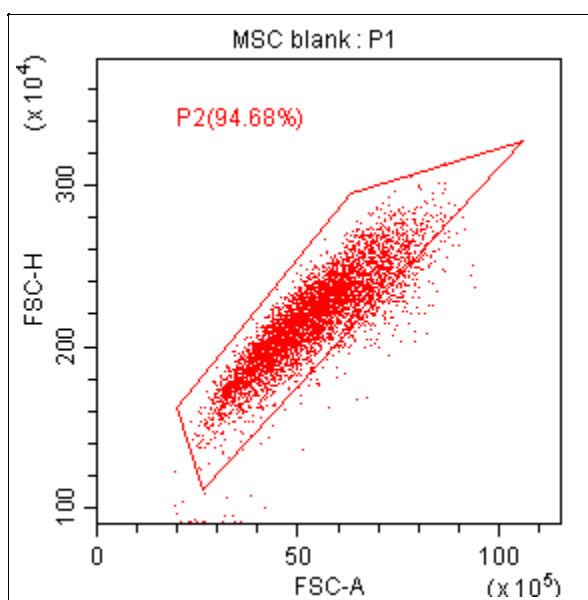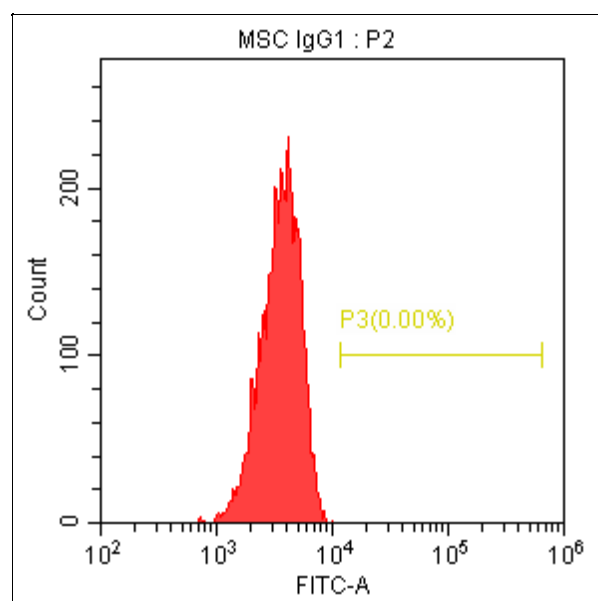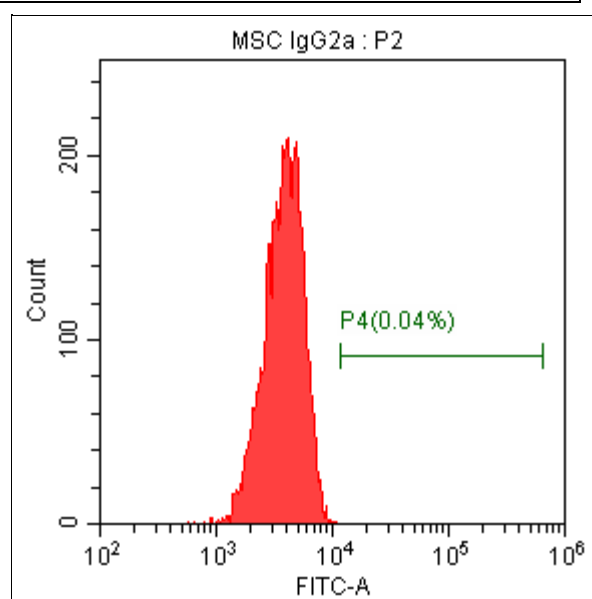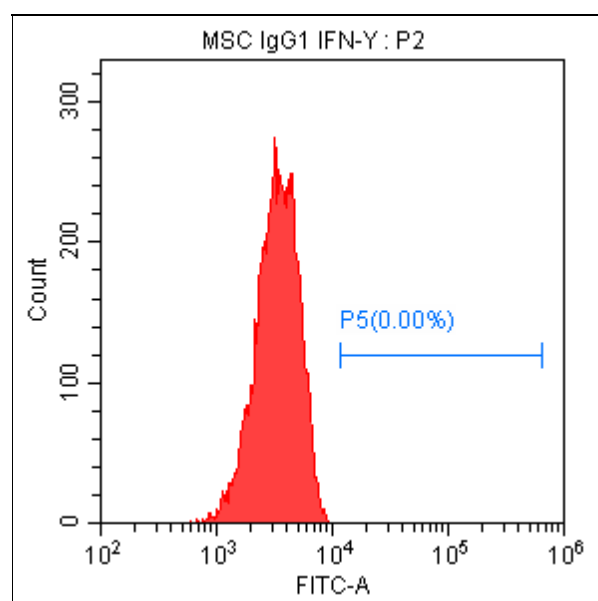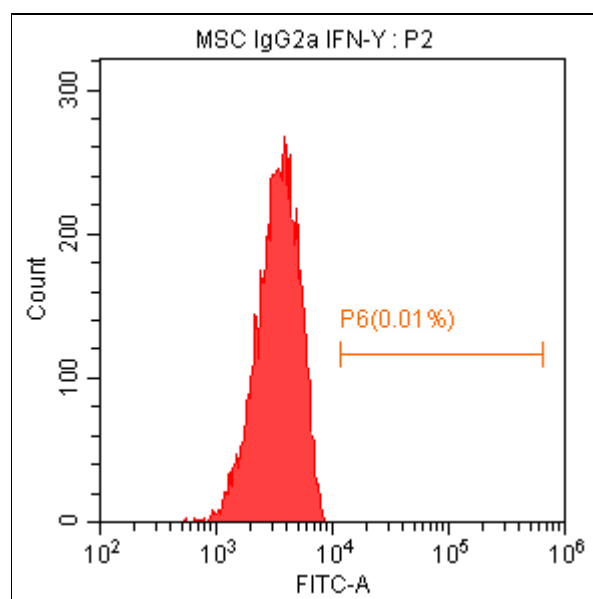

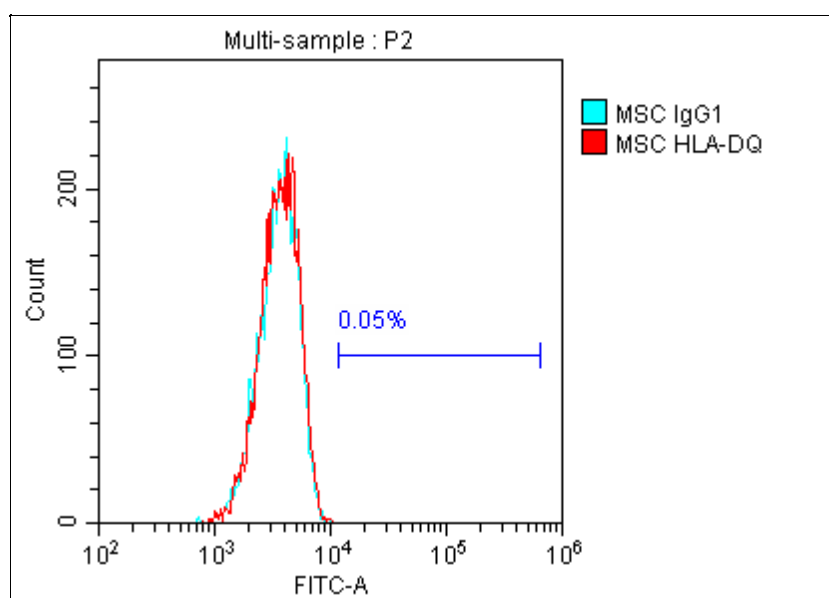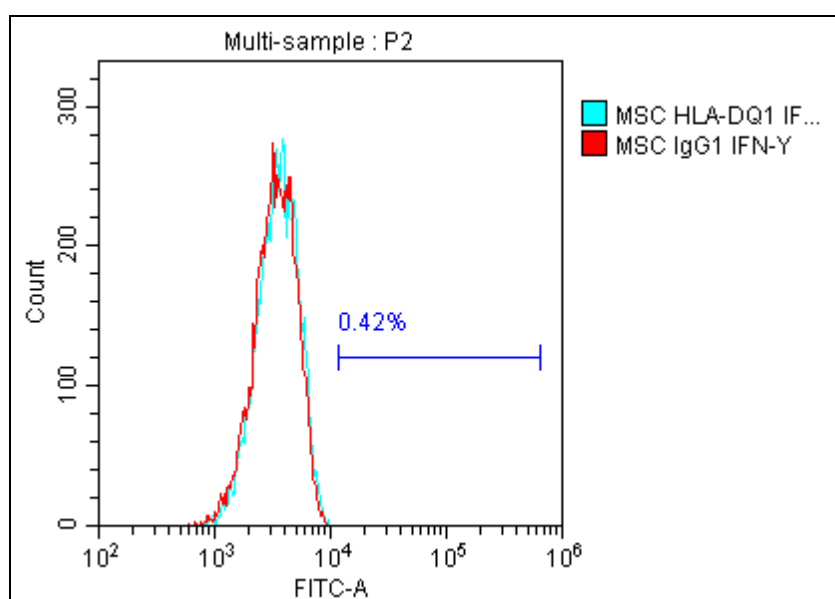

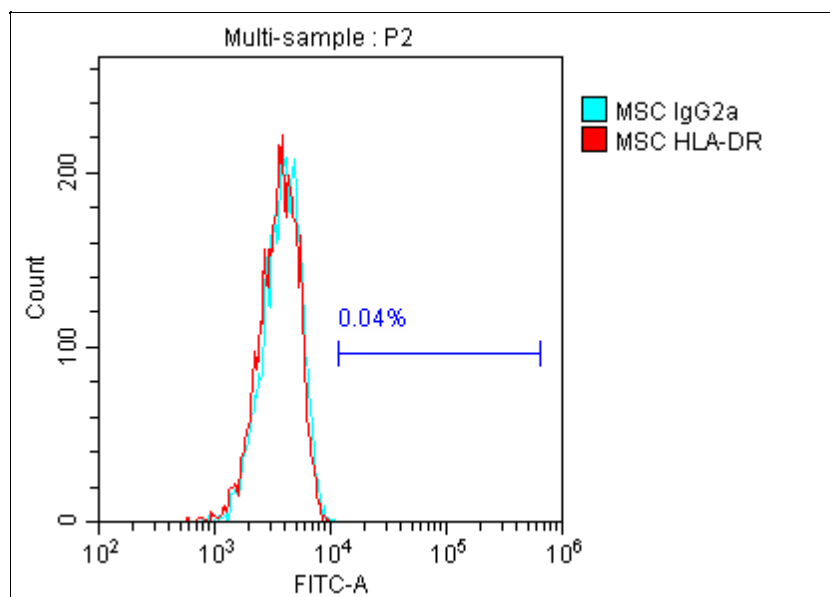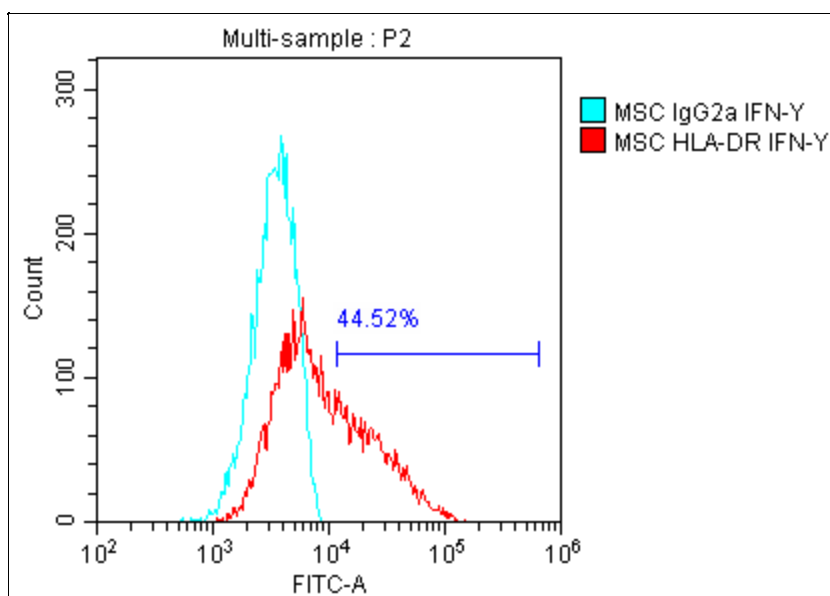

Supplement: Supplementary file 4 [file Data_Sheet_2.ZIP › raw data 1/figure4A HLADR HLADQ.pdf]

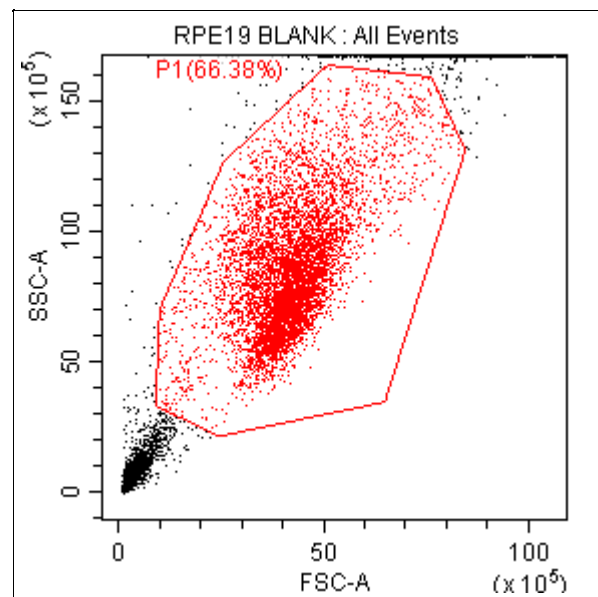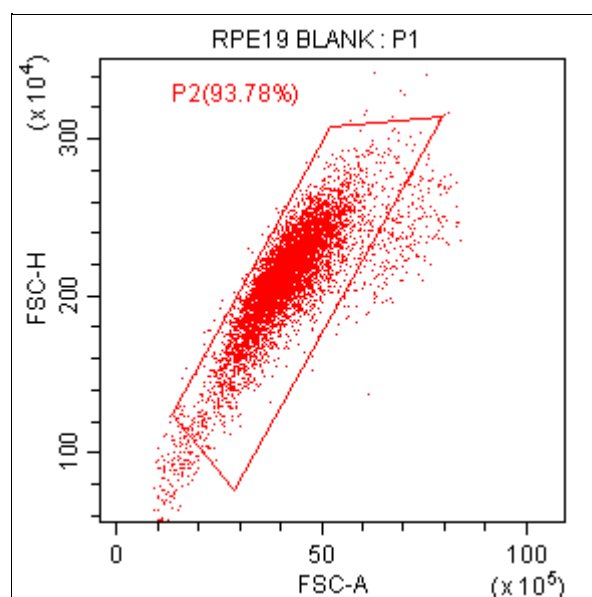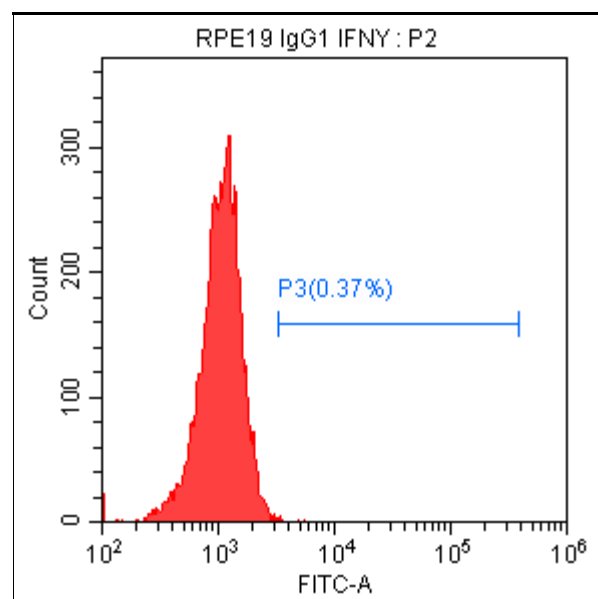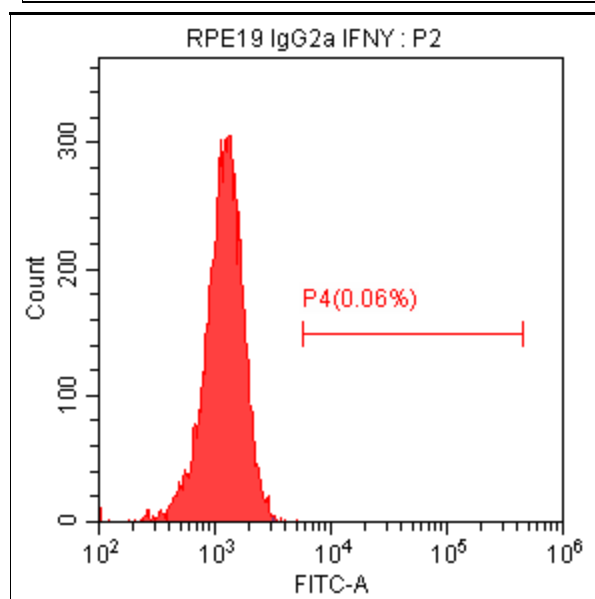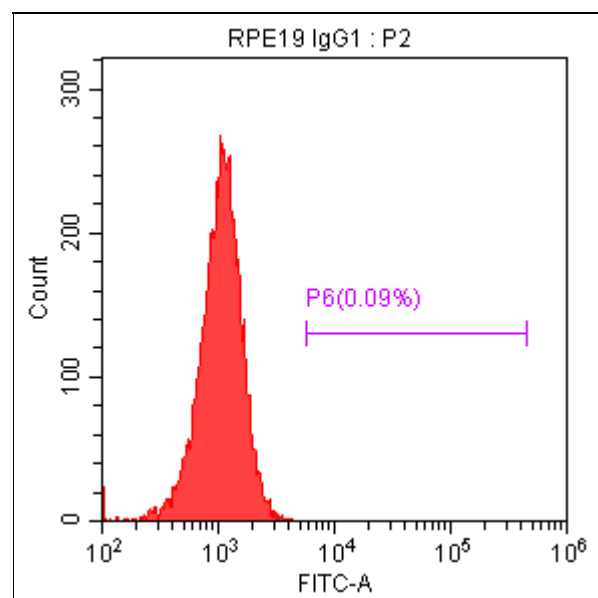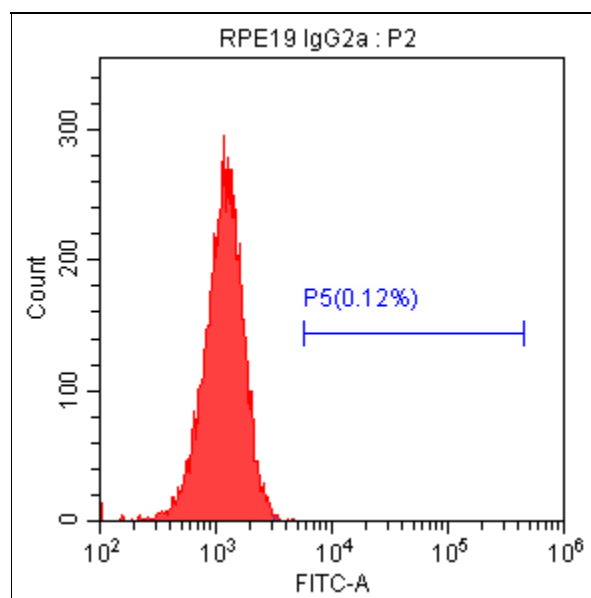

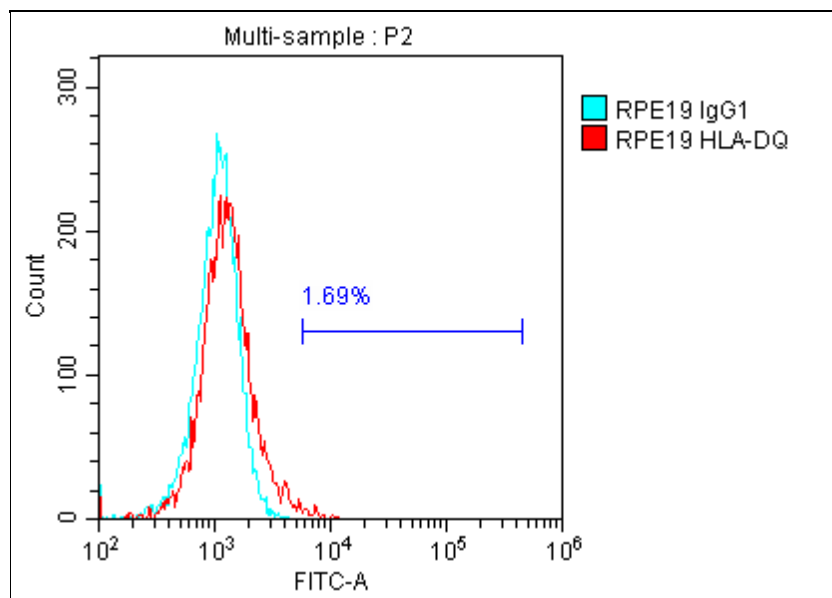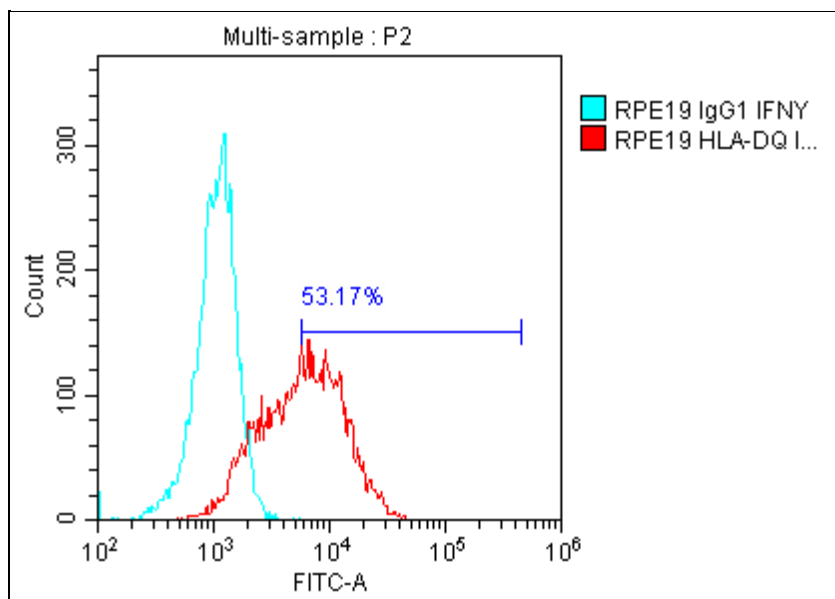

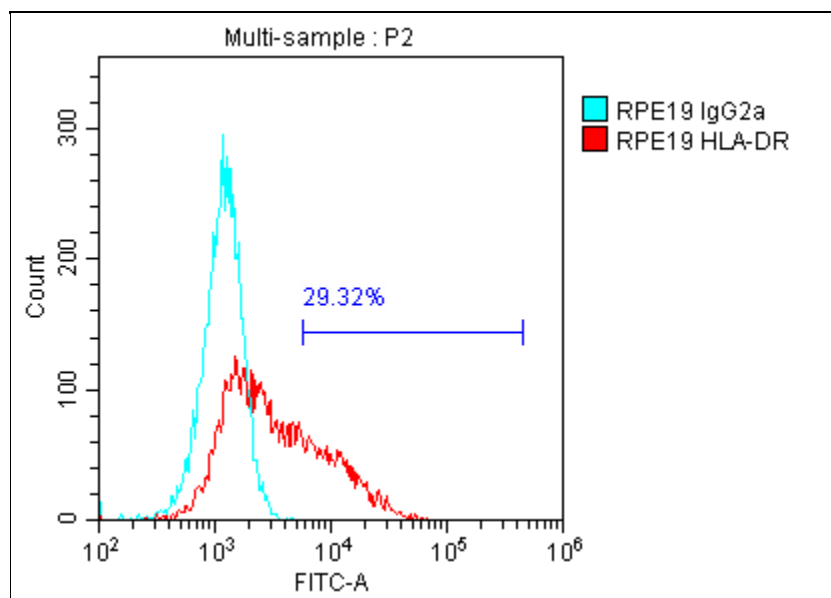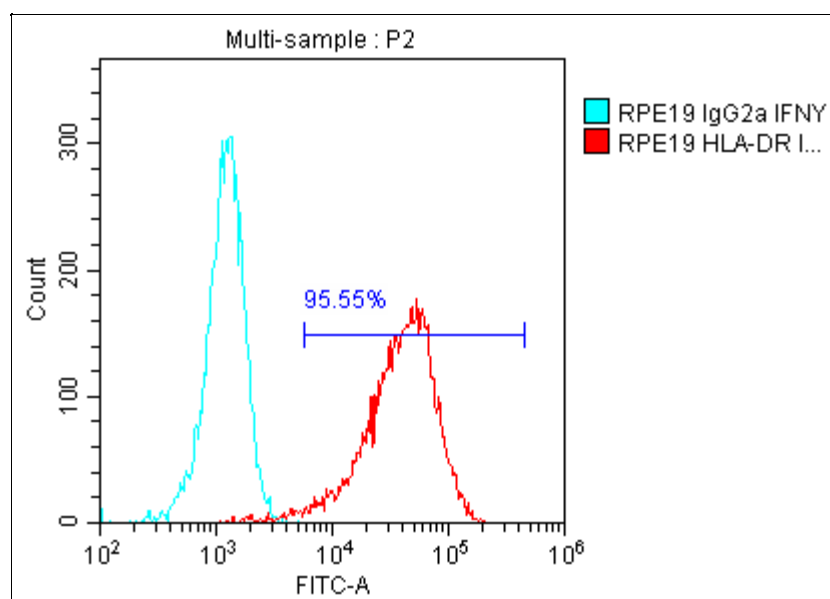

Supplement: Supplementary file 4 [file Data_Sheet_2.ZIP › raw data 1/figure4C HLADQ.pdf]
