# Supplementary material for: Human Amniotic Epithelial Stem Cell-Derived Retinal Pigment Epithelium Cells Repair Retinal Degeneration
Source: Front Cell Dev Biol. 2021 Sep 28;9:737242. doi: 10.3389/fcell.2021.737242 (PMC8505778; doi:10.3389/fcell.2021.737242)
Supplement: Supplementary file 4 [file Data_Sheet_2.ZIP › raw data 1/figure 1 C-F K.pdf]

	Sample Name	Subset Name	Count
	lijinying 20170920 00015602.LMD	*Lymphocytes*	
	lijinying 20170920 00015603.LMD	Lymphocytes	

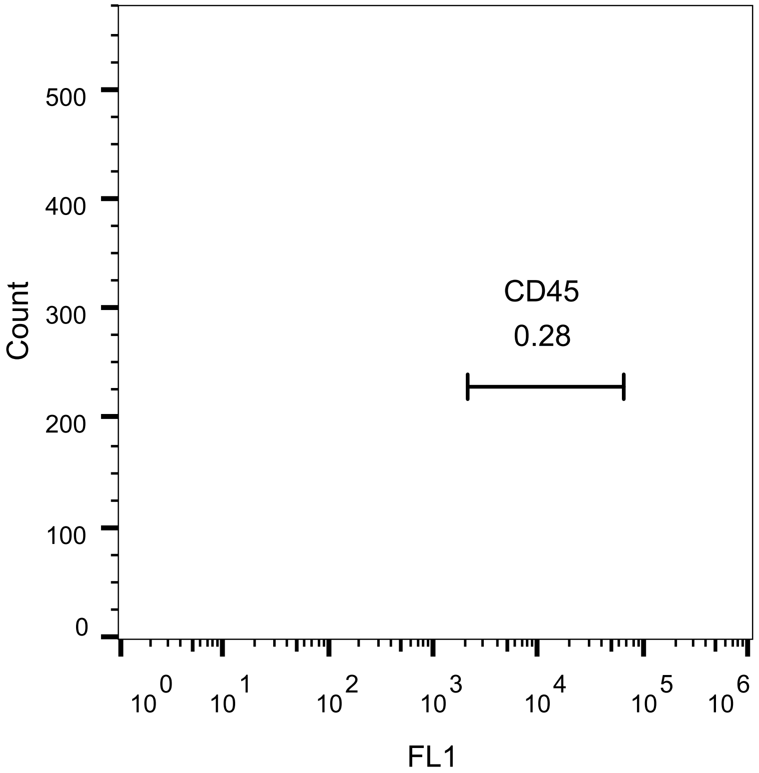

	Sample Name	Subset Name	Cour
	lijinying 20170920 00015614.LMD	Lymphocytes	
	lijinying 20170920 00015615.LMD	Lymphocytes	

8970

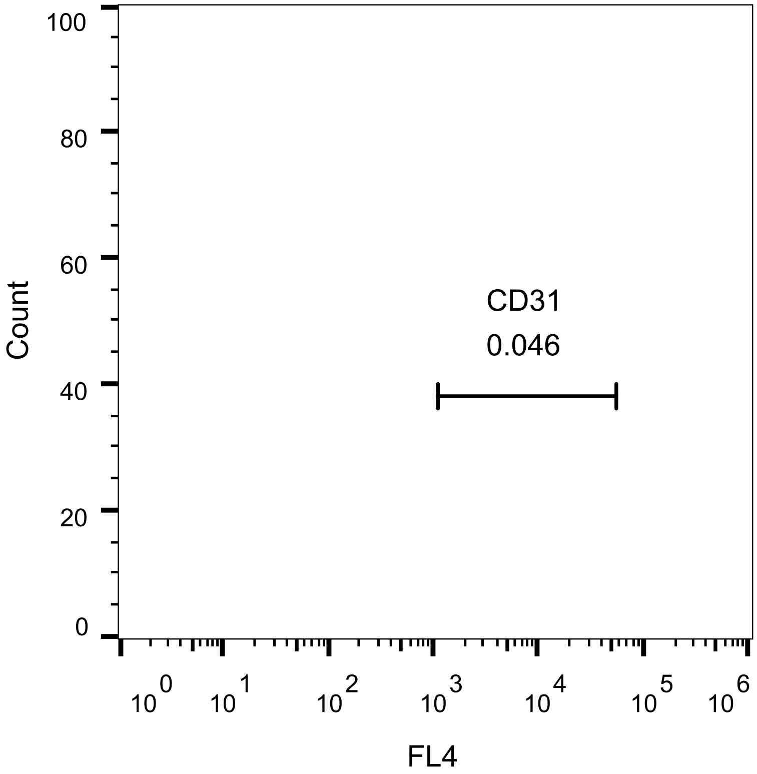

nt
8660
8734

	Sample Name	Subset Name	
	lijinying 20170920 00015602.LMD	Lymphocytes	
	lijinying 20170920 00015604.LMD	Lymphocytes	

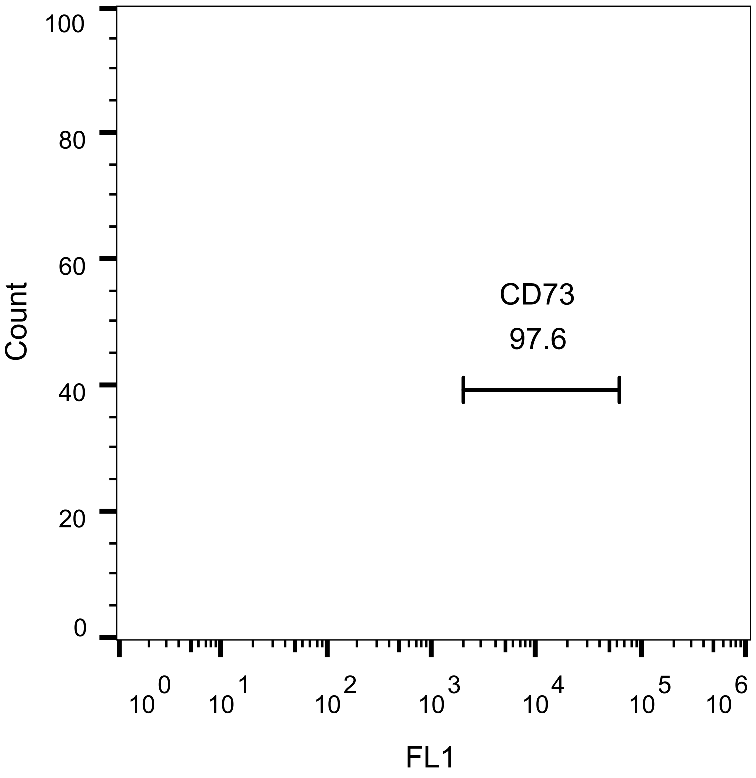

Count

	Sample Name	Subset Name
☐	yuanweixin 20150519 00045050.LMD	Lymphocytes
☐	yuanweixin 20150519 00045052.LMD	Lymphocytes

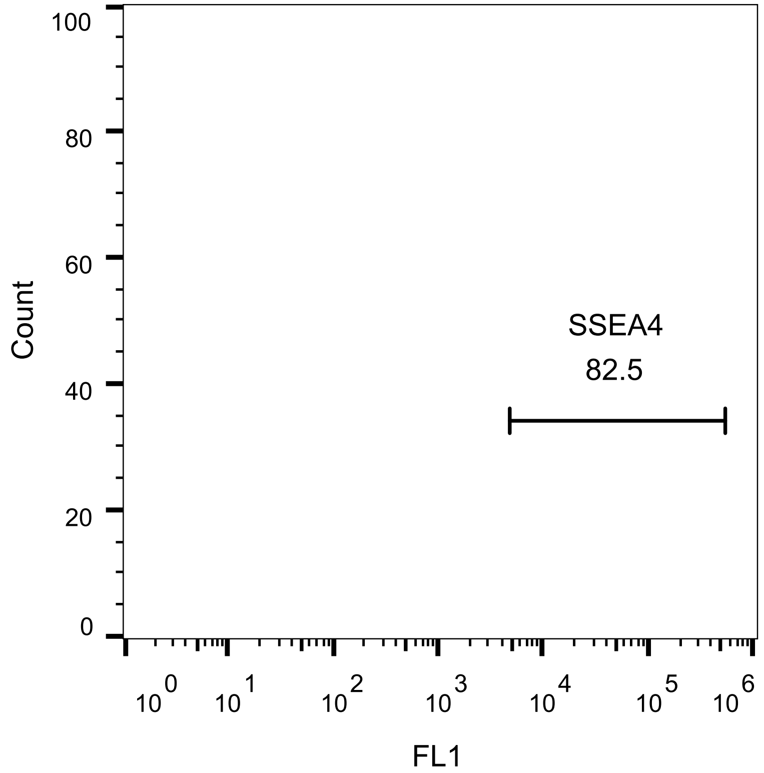

	Count
	7309
	6654

Sample Name	Subset Name	Count
nying 20170920 00015611.LMD	Lymphocytes	89
nying 20170920 00015612.LMD	Lymphocytes	91

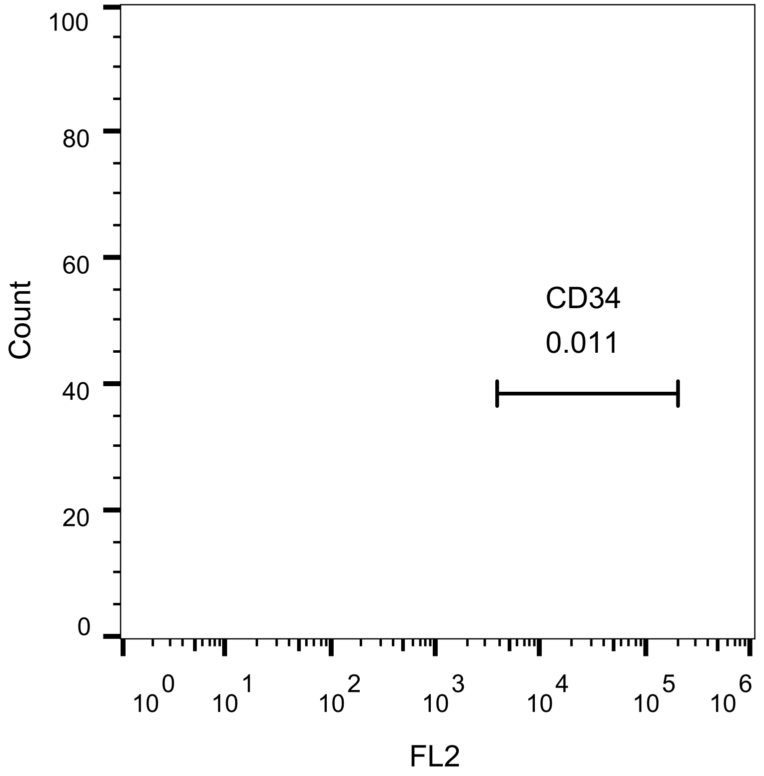

94
89
