# Supplementary material for: Human Amniotic Epithelial Stem Cell-Derived Retinal Pigment Epithelium Cells Repair Retinal Degeneration
Source: Front Cell Dev Biol. 2021 Sep 28;9:737242. doi: 10.3389/fcell.2021.737242 (PMC8505778; doi:10.3389/fcell.2021.737242)
Supplement: Supplementary file 4 [file Data_Sheet_2.ZIP › raw data 1/figure 1G-H CD90 CD105.pdf]

	Sample Name	Subset Name	Cou
	A06 SHYAH0098C0 IgG1 PE .fcs	Lymphocytes	
	A07 SHYAH0098C0 CD90.fcs	Lymphocytes	

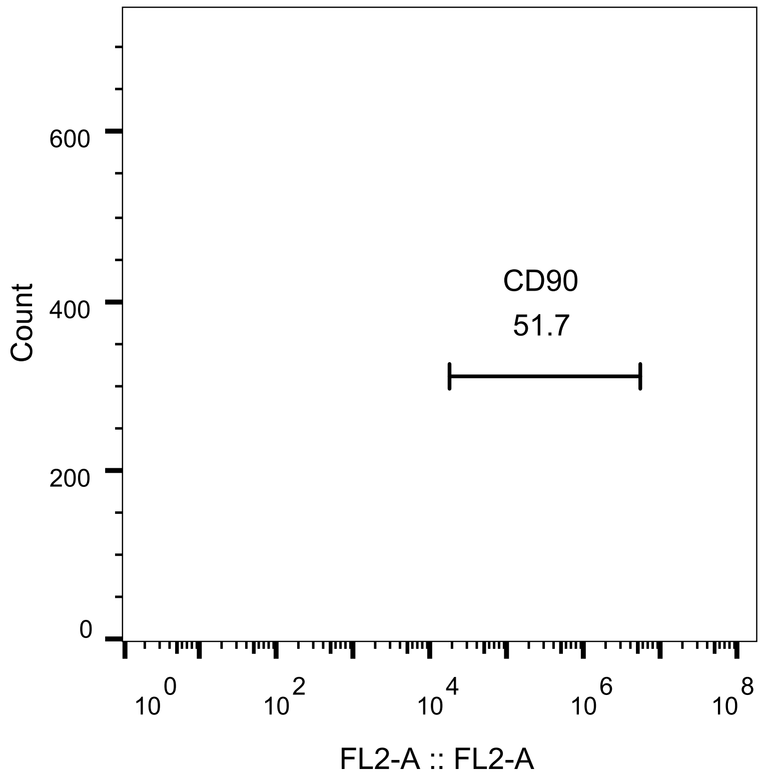

nt
14824
14869

	Sample Name	Subset Name	
	A06 SHYAH0098C0 IgG1 PE .fcs	Lymphocytes	
	A08 SHYAH0098C0 CD105.fcs	Lymphocytes	

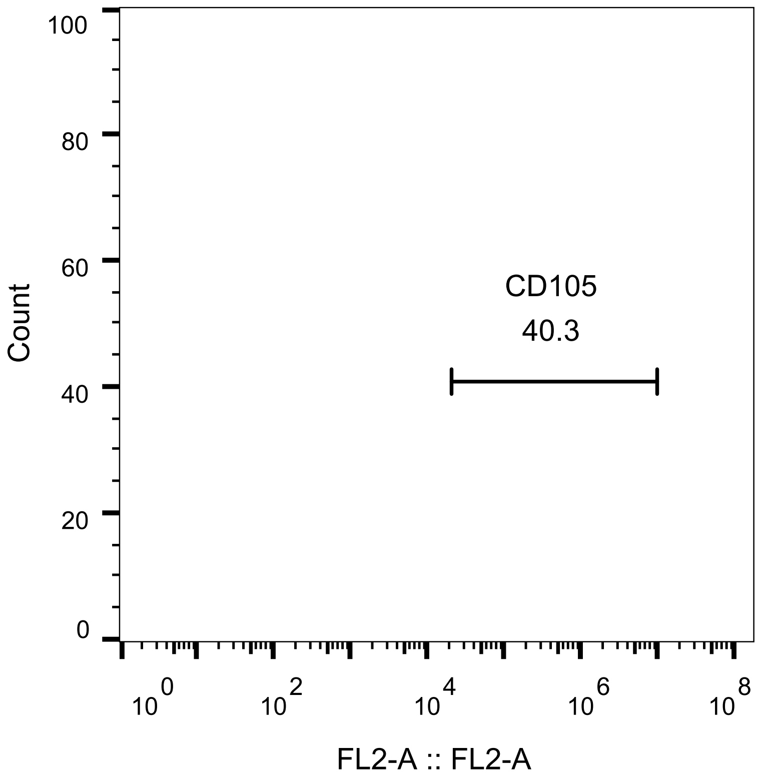

Count
14824
13922
